# Comprehensive Insights into Sulfaguanidine in the Solid State: An Experimental and Computational Study

**DOI:** 10.1021/acs.cgd.3c01384

**Published:** 2024-01-17

**Authors:** Alexandre Widauer, Tom L. Petrick, Doris E. Braun

**Affiliations:** Institute of Pharmacy, University of Innsbruck, Innrain 52c, 6020 Innsbruck, Austria

## Abstract

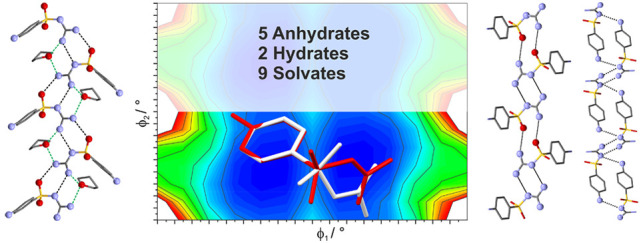

A thorough re-examination of sulfaguanidine’s (SGD) solid-state behavior was conducted, 65 years after the initial report on SGD polymorphism. This investigation focuses on the polymorphic nature of the compound, the formation of hydrates and solvates, and the pivotal role of experimental and computational methods in screening, assessing stability, and understanding transformation processes. The findings confirm the presence of five anhydrates (**AH-I**–**V**), two monohydrate polymorphs (**Hy1-I** and **Hy1-II**), and nine solvates (with *tetrahydrofuran*, *methanol*, *ethanol*, *t-butanol*, *acetone*, *cyclohexanone*, *dimethyl sulfoxide*, *dimethyl formamide*, and *dimethyl acetamide*). Notably, nine novel structures–two anhydrates and seven solvates–are reported, solved from powder X-ray diffraction data. Calorimetric measurements have revealed that **AH-II** is the thermodynamically stable polymorph at room and low temperatures. In contrast, **AH-I** emerges as the stable polymorph at higher temperatures, yet it exhibits remarkable kinetic stability at RT and demonstrates greater stability in terms of hydration. The anhydrate forms exhibit distinctive packing arrangements, while the two hydrates share a close structural resemblance. Among the seven structurally characterized solvates, only the tetrahydrofuran and dimethyl sulfoxide solvates are isostructural. Controlled desolvation experiments enabled the formation of **AH-I**, **AH-II**, and, notably, **AH-V** for the first time. The anhydrate and monohydrate crystal structure prediction studies reveal that the computed lowest-energy structures correspond to experimentally observed forms and propose models for the elusive **AH-IV** structure. Overall, the exploration of SGD’s solid-state landscape confirms a rich array of highly stable H-bonding motifs and packing arrangements, positioning this study as an ideal model for complex solid-state systems and shedding light on its intricate solid-state nature.

## Abstract 2

This study explores the solid-state forms of the antibiotic drug sulfaguanidine, unveiling nine previously unknown forms. Sulfaguanidine’s tendency to transform into a hydrate poses challenges in identifying and characterizing the solvates and anhydrates. Nonetheless, these (unstable) solvates and stable hydrate forms play a pivotal role as intermediates in the formation of anhydrous polymorphs, provided that carefully controlled desolvation conditions are employed.

## 1 Introduction

The solid-state forms of pharmaceutical compounds have long been a subject of intense research, owing to their profound influence on drug performance and efficacy.^1−4^ Polymorphs represent different crystalline arrangements of the same compound, offering distinct physical and chemical properties.^5−7^ Understanding and characterizing these polymorphic forms is not merely an academic pursuit, but a crucial step in pharmaceutical development. Hydrates and solvates introduce an additional layer of complexity to the study of solid forms.^8,9^ The inclusion of water or other solvent molecules within the crystalline lattice can drastically alter its physical and chemical properties.^10,11^ Moreover, understanding the desolvation of these forms is paramount for ensuring product stability.^12,13^

The significance of polymorph screening becomes evident when we consider the abrupt emergence^14,15^ or disappearance^16,17^ of a polymorphic form during the manufacturing and storage of a product. These transformations can result in serious consequences, including risks to patient safety, financial implications, *etc*. The likelihood of encountering polymorphs of a compound depends on conformational flexibility^18−20^ and the diversity in potential crystal packing arrangements.^21−23^ In accordance with Kuhnert-Brandstätter,^24^ “Probably every substance is potentially polymorphous. The only question is, whether it is possible to adjust the external conditions in such a way that polymorphism can be realized or not”, the probability of discovering polymorphs of a compound is influenced by time and resources allocated to research on that particular compound. This assertion is exemplified by recent findings of new polymorphs of substances like dapsone,^25^ thymine,^26^ indomethacine,^27^ ritonavir,^28^ etc.

Active pharmaceutical ingredients (APIs) frequently encounter solvents or their vapors during various stages of industrial processes, including recrystallization, wet granulation, and spray-drying. An inadvertent generation of solvates^29^ can result in significant alterations in the characteristics of the compounds, potentially leading to challenges in drug development.^30,31^ However, there are instances where a specific polymorph or a high-purity product can only be achieved through the formation of a specific solvate.^12,13^ These solid forms may remain beyond the reach of conventional crystallization techniques, making it essential to give careful consideration to a precisely controlled solvate formation process.

Various experimental techniques are employed to generate solvates,^32^ including methods such as slurry bridging, evaporation, cooling, antisolvent addition, and solvent vapor deposition.^33^ However, the process of selecting appropriate solvents to obtain desired solvates is often time-consuming and costly due to the lack of a clear guidance.^34^ Therefore, the ability to predict which solvents are likely to form solvates with a given compound has become increasingly important.^35^ Currently, several methods are utilized for predicting solvate formation propensity, e.g., machine learning and knowledge-based models,^36,37^ the COSMO-RS approach,^38^ and approaches based on the CSD data.^39−43^ Consequently, exploring the primary driving forces behind solvate formation is crucial, as it can offer valuable insights for selecting appropriate solvents to either encourage or inhibit the formation of specific solvates. However, understanding of the mechanisms underlying solvate formation remains elusive.^44^

**Figure 1:**
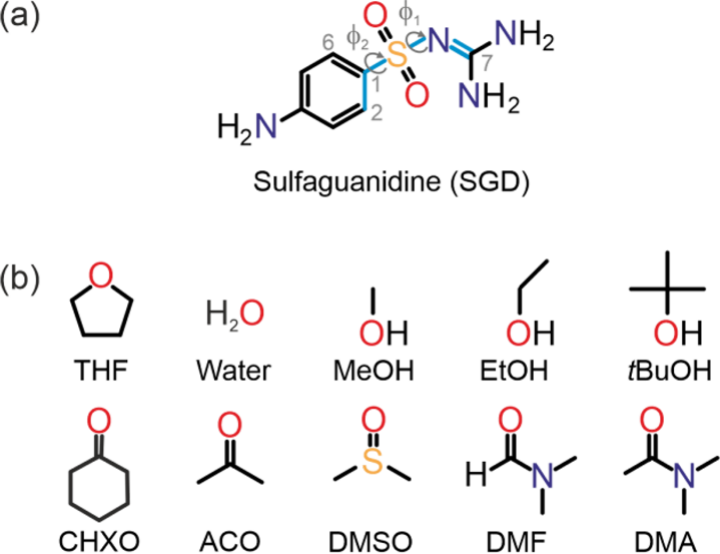
(a) Sulfaguanidine (SGD), with key torsions highlighted (φ_1_ and φ_2_). (b) Solvent molecules forming solvates/hydrates with SGD.

The objective of this study is to elucidate the underlying principles governing the formation and stability of polymorphs and solvates, employing both experimental and computational methods. The model compound, 4-amino-*N*-(aminoiminomethyl)benzenesulfonamide, better known as sulfaguanidine (SGD, Figure 1), is an API belonging to the group of sulfonamides. Sulfonamides were among the first classes of antibiotics to be discovered, and they were initially employed as systemic medicines with selective antibacterial effects.^45^ Due to its poor absorption in the intestines,^46^ SGD was initially used as the first antimicrobial agent in the sulfonamide group for the localized treatment of intestinal infections such as bacillary dysentery.^47^ In the past, sulfonamides were widely used in treating various bacterial infections. However, in recent times, their clinical application has been greatly restricted due to the emergence of strong antibiotic resistance and the development of more effective medications.^48^ Presently, SGD finds its use primarily in veterinary medicine.^49,50^

SGD possesses a moderate level of conformational flexibility and exhibits multiple H-bonding donor and acceptor groups. These characteristics have the potential to give rise to a diverse range of noncovalent interactions and packing arrangements, thereby exerting a significant influence on the variety of solid-state forms that can be generated. To date, three to four polymorphs [Kuhnert-Brandstätter et al.: I–III,^51−53^ Alberola et al.: I–III,^54^ Mesley and Houghton: B-D,^55^ Yang and Guillory: II–V^56^ (IV and V show identical IR spectra, according to the authors)], two monohydrates,^53−58^ an acetone solvate,^54^ and an amorphous phase^55^ have been reported. Note that the nomenclature for the forms is not consistent, and one of the two hydrates was solved as two different tautomers (amino and imino). Furthermore, cocrystals of SGD with thiobarbituric acid^47^ (anhydrate and hydrate), 1,10-phenanthroline,^47^ antipyrine,^59^ 4-nitrobenzoic acid^60^ (anhydrate and acetonitrile solvate), 3-nitrobenzoic acid,^60^ phenazine,^60^ and 1,2-di(pyridyl)ethylene^60^ have been reported.

In this study, we discovered eight additional solvates and a new anhydrate polymorph of SGD, hereafter referred to as **AH-V**. The crystal structures of seven of the solvates and two anhydrates (**AH-I** and **AH-V**) were determined using powder X-ray diffraction (PXRD). Furthermore, all solid-state forms underwent comprehensive characterization using PXRD, hot-stage microscopy (HSM), differential scanning calorimetry (DSC), thermogravimetric analysis (TGA), Fourier transform infrared (FT-IR) spectroscopy, and gravimetric moisture (de)sorption analysis. Additionally, we investigated the stability of the solvates during storage. The experimental investigations were complemented by generating the crystal energy landscapes for both the anhydrate and monohydrate forms. These predicted structures, in conjunction with the experimental ones, served as the basis for elucidating the principal driving forces behind polymorphism and solvate (hydrate) formation of SGD.

## 2 Materials and Methods

### 2.1 Materials

SGD was obtained from Apoka (purity >99%) and consisted of the monohydrate (**Hy1-I**). Organic solvents of analytical grade were purchased from various commercial suppliers and used without further purification.

### 2.2 Experimental Solid Form Screening

#### 2.2.1 Recrystallization from the Melt

The melt of SGD (starting from **AH-I**) was placed onto a hot-bench at specified temperatures ranging from 70 to 130 °C. Subsequently, the crystallization products were transferred to a hot-stage microscope, and the resulting phases were identified based on the phase transition behavior and melting points.^51−53^

#### 2.2.2 Solvent Evaporation Experiments

Saturated solutions of **AH-I** (15 mg) were prepared at room temperature (RT) in 30 different solvents. The solutions were subsequently filtered, transferred onto a watch glass, and left to evaporate under ambient conditions. The resulting crystalline products were analyzed using PXRD. For more details, please refer to ESI, section 3.1.

#### 2.2.3 Cooling Crystallization Experiments

In the pursuit of obtaining solubility data, cooling crystallization experiments were conducted. The temperature-dependent solubility of **AH-I** was determined in 29 different solvents using the Crystal16 crystallization system (Technobis, Alkmaar, Netherlands). The samples were subjected to a precisely defined temperature program. The lower temperature limit was always set at 5 °C, while the upper limit was approximately 10 °C below the boiling point of the respective solvent. Heating and cooling rates of 0.2 °C min^–1^ were used. For more details please see ESI, section 3.4.

#### 2.2.4 Liquid-Assisted Grinding Experiments

Eight drops of solvent (or two drops in the case of dimethyl acetamide and diethylene glycol) were added to 80 mg of **AH-I** (41 different solvents used). The resulting mixture was then subjected to milling in stainless steel vessels containing three 0.5 cm balls of the same material. The milling process was conducted using a Retsch ball mill MM 500 (Haan, Germany) at a frequency of 15 Hz for a duration of 30 min. Subsequently, the resulting samples were analyzed using PXRD. For more details, please refer to ESI, section 3.2.

#### 2.2.5 Slurry Experiments in Organic Solvents

Approx. 0.1 to 0.3 mL of solvent was introduced into small vials containing 100 mg of SGD **AH-I**, **AH-II**, or **Hy1-I**, equipped with a stirring bar. The sealed vials were then placed in a temperature-controlled water bath and stirred for approximately 2 weeks within the temperature range of 10 to 30 °C (cycling). At regular intervals samples were withdrawn and examined using PXRD to detect the formation of any new solid-state forms. Slurry experiments were conducted using 34 different solvents. For details, see ESI, section 3.3.

#### 2.2.6 Desolvation Studies

The studies were conducted on **Hy1-I** and eight solvates. The samples were dispensed onto well plates and stored under five different conditions: (1) 25 °C/0% RH; (2) 25 °C/50% RH (room conditions); (3) 25 °C/100% RH; (4) 40 °C/75% RH; (5) 60 °C/12% RH. Subsequently, the solid-state forms were periodically examined using PXRD.

### 2.3 Preparation of the SGD Solid-State Forms

#### 2.3.1 Monohydrate **Hy1-I**

The hydrate was obtained from cooling crystallization experiments using water, alcohols, 1,4-dioxane, and acetonitrile.

#### 2.3.2 Anhydrate **AH-I**

**Hy1-I** was dried to a constant weight using an IR drying balance (HX204 Moisture Analyzer, Mettler Toledo, Vienna, Austria) at 150 °C.

#### 2.3.3 Anhydrate **AH-II**

**AH-II** was obtained by storing **Hy1-I** at 25 °C and 0% RH (using P_2_O_5_) for a period of 3–5 days.

#### 2.3.4 Anhydrate **AH-III**

**AH-III** was prepared by adapting the method described by Kuhnert-Brandstätter,^52^ which involves annealing the melt of **AH-I** at approximately 70 °C.

#### 2.3.5 Anhydrate **AH-V**

**AH-V** was produced by storing the THF solvate at 25 °C and 0% RH (using P_2_O_5_) for 1 day.

#### 2.3.6 Solvates

Approximately 500 mg of **AH-I** (or **Hy1-I** in case of **S-MeOH** and **S-EtOH**) were transferred into stirring cylinders, equipped with a magnetic stirrer, and mixed with a solvent volume ranging from 0.3 to 1.5 mL, depending on the solubility of the anhydrate. The suspensions were placed in a temperature-controlled water bath, with cyclic temperature variations between 10 and 30 °C, and stirred for 1 to 2 days.

### 2.4 Computational Solid Form Screening and Interaction Energy Calculations

#### 2.4.1 Potential Energy Surface Scans

PES scans were carried out using Gaussian 09^61^ at the PBE0/6-31G(d,p) level of theory. The dihedral angles, as indicated in Figure 1, were systematically scanned in increments of 30°.

#### 2.4.2 Computational Generation of the Anhydrate and Monohydrate Crystal Energy Landscapes

Crystal structure prediction (CSP) studies were performed for SGD anhydrates (*Z*′ = 1 and 2) and monohydrates (*Z*′ = 1). Rigid-body CrystalPredictor v2^62−64^ searches were conducted across the 59 most common space groups, starting from four SGD conformations (ESI, Figure S2). 2.5 × 10^6^ anhydrate and 1.0 × 10^6^ monohydrate structures were generated. Structures within the 25 kJ mol^–1^ range of the global minimum structure were reoptimized first with DMACRYS^65^ and subsequently with CrystalOptimizer v2.4.8^66^ using a distributed multipole representation of the charge density^67^ and considering all dihedral angles involving heteroatoms. Conformational energies and distributed multipoles were calculated at the PBE0/6-31G(d,p) level using Gaussian 09.^61^ All other intermolecular forces were modeled using an atom–atom *exp-6* form with the FIT potential.^65,68^

The lattice energies of the structures were then recalculated using CASTEP v20.11^69^ (single-point energy calculations). This involved employing the PBE generalized gradient approximation exchange-correlation density functional,^70^ ultrasoft pseudopotentials,^71^ and the inclusion of the MBD* dispersion correction.^72^ The most stable structures (15 kJ mol^–1^ range) were then reoptimized using the same settings as described for the single-point calculations. The number of *k*-points was determined to provide a maximum spacing of 2π × 0.07 Å^–1^, and a basis set cutoff of 780 eV was applied. Convergence criteria were set as follows: <2 × 10^–5^ eV per atom, atomic displacements <1 × 10^–3^ Å, maximum forces <5 × 10^–2^ eV Å^–1^, and maximum stresses <0.1 GPa.

#### 2.4.3 Multicomponent Hydrogen-Bond Propensity Calculations

A selection of 41 potential solvents (ESI, Table S5), the same as used experimentally, was chosen and evaluated based on their MCHB scores, which were computed using Mercury.^73^ The propensity for the most favorable heteromeric interaction between SGD and a solvent (SGD–S) was compared to the most favorable homomeric interaction, either SGD–SGD or solvent–solvent (S–S). To estimate the likelihood of solvate formation, the difference was calculated, Δ_HBP_ = *P*_SGD–S_ – [max(*P*_SGD–SGD_, *P*_S–S_)], where higher values signify a greater likelihood of solvate formation.

#### 2.4.4 Pairwise Intermolecular Energy Calculations

To compute the pairwise intermolecular energy,^74−76^ CrystalExplorer V17^77^ was used, along with the PBE-MBD*^78,79^ optimized structures, applying a 3.8 Å radius. Gaussian 16^80^ was employed to compute B3LYP/6-31G(d,p) molecular wave functions. From these wave functions, the densities of unperturbed monomers were derived to extract four distinct energy components: electrostatic (*E*_E_), polarization (*E*_P_), dispersion (*E*_D_), and exchange-repulsion (*E*_R_).

### 2.5 Powder X-ray Diffraction (PXRD) and Structure Solution from PXRD

An X’Pert Pro diffractometer (PANalytical in Almelo, Netherlands) was employed for the study. This diffractometer was equipped with a PIXcel1D detector and operated with a Cu Kα_1,2_ radiation source at 40 kV and 40 mA. The instrument was configured on a θ/θ coupled goniometer in a transmission geometry setup. The measurements encompassed a 2θ range spanning from 2° to 40°, utilizing a step size of 0.013°, with a duration of 40 s per step. Alternatively, for structure solution, measurements were conducted from 2° to 70°, using steps sizes of 0.013° or 0.007°, and each step lasted for 800 or 1600 s (multichannel mixed-signal).

The diffraction patterns of **AH-I**, **AH-V**, **S-THF**, **S-MeOH**, **S-*t*BuOH**, **S-ACO**, **S-DMSO**, **S-DMF**, and **S-DMA** were indexed using DICVOL. The space groups were determined based on a statistical assessment of systematic absences,^81^ using DASH.^82^ From the cell volume analysis, it was determined that **AH-I** has a *Z*′ value of 3, while **AH-V** is *Z*′ = 1. All solvates, except for **S-ACO**, exhibit a 1:1 ratio. In contrast, **S-ACO** shows a 2:1 ratio (SGD/ACO). **S-DMA** is a *Z*′ = 2 structure, while the other solvates are *Z*′ = 1. Pawley refinement^83^ was employed to extract the intensities and correlations. Simulated annealing was used to optimize the models based on the diffraction data set in direct space. The internal coordinate descriptions were derived from the PBE0/6-31G(d,p) gas phase global conformational minima, with O–H and N–H distances normalized to 0.9 Å and C–H to 0.95 Å. Each of the structures was solved using 100 simulated annealing runs, each consisting of 2 × 10^8^ moves. Each SGD molecule was allowed 6 external and 2 internal degrees of freedom (φ_1_ and φ_2_, Figure 1), and solvent molecules were allowed 6 external and 0–1 internal degrees of freedom. All chosen structure solutions had refined to a χ^2^ ratio of <4.9 (profile χ^2^/ pawley χ^2^). The optimized structures (PBE-MBD*), with O–H and N–H distances normalized to 0.9 Å and C–H distances normalized to 0.95 Å, were then used as the starting point for rigid-body Rietveld refinements^84^ using TOPAS V7.12.^85^ The background was modeled with Chebyshev polynomials. Additional details can be found in ESI, Tables S10 and S11.

### 2.6 Thermal Analysis

#### 2.6.1 Hot-Stage Microscopy (HSM)

An Olympus BH2 polarization microscope (Olympus Optical GmbH, Austria), equipped with a Kofler hot stage from Reichert Thermovar (Vienna, Austria) was used in conjunction with an Olympus DP71 digital camera.

#### 2.6.2 Differential Scanning Calorimetry (DSC)

The analyses were carried out using a DSC7 instrument (PerkinElmer, Norwalk, Connecticut, USA) in combination with the Pyris 8.0 Software. Precise sample weighing was performed with a UM3 ultramicrobalance (Mettler, Greifensee, Switzerland). Approximately 2–5 mg of each sample was utilized, and the analyses involved heating rates of 1, 2, 5, or 10 °C min^–1^, with a nitrogen purge gas flow of 20 mL min^–1^. Temperature (onset) and enthalpy values were determined based on a minimum of three measurements, and error estimation was conducted with a 95% confidence interval. The calorimeter was calibrated using benzophenone (48.0 °C) and caffeine (236.2 °C) for temperature calibration and indium (28.45 J g^–1^) for enthalpy calibration.

#### 2.6.3 Thermogravimetric Analysis (TGA)

The measurements were conducted using a TGA7 system (PerkinElmer, Norwalk, CT, USA) and the Pyris 8.0 Software. Approximately 3 mg of the sample were carefully weighed into a platinum pan. Temperature calibration was performed using two-point calibration with ferromagnetic materials (Alumel and Ni, Curie-point standards, PerkinElmer). Various heating rates, including 2, 5, and 10 °C min^–1^, were applied, and dry nitrogen served as the purge gas (sample purge: 20 mL min^–1^, balance purge: 40 mL min^–1^).

### 2.7 Fourier-Transform Infrared Spectroscopy

Infrared spectra were recorded using a Bruker Vertex 70 FTIR spectrometer (Bruker Analytische Messtechnik GmbH, Germany) with a temperature-controlled diamond ATR (PIKE GaldiATR, Madison, US) crystal. The spectral range covered 4000 to 400 cm^–1^ with an instrument resolution of 2 cm^–1^ (32 scans per spectrum). Heating experiments were performed at a heating rate of 2 °C min^–1^.

### 2.8 Gravimetric Moisture (De)sorption Experiments

Moisture (de)sorption investigations were carried out using the automatic multisample gravimetric moisture sorption analyzer SPS23 (ProUmid, Ulm, Germany). The instrument underwent calibration using saturated salt solutions following the supplier’s guidelines. Each analysis employed approximately 100–150 mg of the sample.

The measurement cycles commenced at 0% relative humidity (RH) and followed an initial stepwise sorption process until reaching 95% RH. This was followed by a desorption cycle down to 0% RH. RH changes were programmed at 5%, and equilibrium conditions for each step were defined as a mass constancy within ±0.001% over 48 min, with a minimum time limit of 2 h and maximum time limit of 2 days per step.

## 3 Results and Discussion

### 3.1 Molecular Features of SGD and Potential Energy Surface Scans

**Figure 2:**
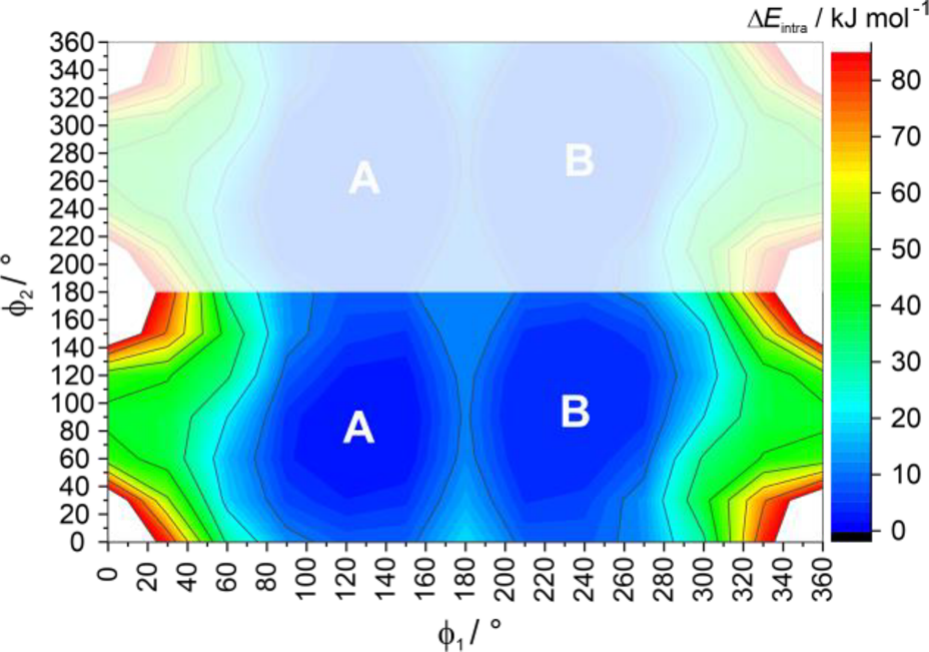
Potential energy surface scan of SGD (dihedral angles depicted in Figure 1).

SGD (Figure 1) exhibits conformational flexibility, features numerous H-bond donor and acceptor groups, and has the potential for forming aromatic interactions. Furthermore, tautomerism (amino/imino) is possible. The potential energy scan of φ_1_ and φ_2_ reveals four low-energy minima. If the −NH_2_ groups were essentially planar, these minima could be reduced to two minima, which are related by inversion symmetry (φ_2_ = C2–C1–S–N = C6–C1–S–N, Figure 1) and are labeled as A and B in Figure 2. In experimental structures, due to the capacity of forming H-bonding interactions, different orientations of the −NH_2_ groups and pyramidalization thereof are expected. As a result, the potential energy scan is not entirely symmetrical. A notable aspect of SGD’s conformational flexibility is the significant rotation potential of the guanidinium moiety (φ_1_) at a relatively low cost in terms of intramolecular energy. This penalty is even lower for the aniline group (φ_2_). Therefore, it can be anticipated that the SGD molecule has the flexibility to adopt conformations within the φ_1_ range of 60–300° and the φ_2_ range of 0–360°.

### 3.2 Experimental Screen for SGD Solid-State Forms

The experimental screen encompassed solvent evaporation, cooling crystallization, liquid-assisted grinding, slurry experiments in solvents, annealing of the melt, as well as systematic desolvation studies and reproduced the **AH-I**, **AH-II**, **AH-III**, one of the monohydrate polymorphs (**Hy1-I**), and the acetone solvate (**S-ACO**). Furthermore, novel solid forms were identified and produced phase pure: one anhydrate (**AH-V**) and seven solvates (**S-DMSO**, **S-DMF**, **S-DMA**, **S-THF**, **S-*t*BuOH**, **S-MeOH**, and **S-CHXO**). An **S-EtOH** solvate was also identified (confirmed by HSM), although this phase was only seen concomitantly with one of the anhydrate forms or **Hy1-I**.

#### 3.2.1 Slurry Experiments and Liquid-Assisted Grinding

The most effective method for discovering SGD solvates involved a process of slurring SGD in a solvent capable of forming solvates within a temperature range of 10 to 30 °C (cycling). In the case of water, **Hy1-I** formed. Remarkably, within just 1–2 days, nine solvates could be obtained phase pure. In the case of experiments involving liquid-assisted grinding, it was feasible to produce **Hy1-I** and four of the solvates (**S-DMSO**, **S-DMF**, **S-DMA**, and **S-*t*BuOH**) phase pure, while **S-THF** and **S-CHXO** were produced as a mixture with **AH-I** and **Hy1-I**. Therefore, it is possible that the increase in temperature resulting from the grinding process and the presence of humidity (experiments were performed at approximately 50% RH) may have adversely affected solvate formation. Moreover, the varying time scales of the slurry and grinding experiments may have influenced the outcomes. Notably, in the case of **S-ACO**, **S-MeOH**, and **S-EtOH**, no solvate formation was observed in the liquid-assisted grinding experiments. In terms of hydrate stability, both slurry and grinding experiments indicate that **Hy1-I** is the stable hydrate polymorph at RT. It is important to mention that the preferred method for producing **S-MeOH** and **S-EtOH** involved using **Hy1-I** as the starting material rather than **AH-I**. For the other solvates, either **AH-I** or **Hy1-I** as the starting material proved effective.

#### 3.2.2 Cooling Crystallization and Solvent Evaporation Experiments

The limited selection of solvents used in successful solvent evaporation and cooling crystallization experiments was primarily due to the low solubility of SGD in several solvents, as elaborated in section 3.4 of the ESI. In both cooling crystallization and solvent evaporation experiments, the predominant outcome was **Hy1-I**. This may be related to the fact that no precautions were taken to ensure working under dry conditions.

Regarding the polymorphism of SGD, only the solvent evaporation experiment conducted with THF yielded a different form, namely, **AH-V**. Microscopic examinations of the crystallization product revealed pseudomorphosis,^86^ indicating that **AH-V** formed via an intermediate phase, namely, **S-THF**.

Earlier studies had documented the formation of various SGD anhydrate polymorphs through solution-based methods.^54−56^ However, attempts to reproduce these procedures did not yield any of the previously reported anhydrate forms. Upon close examination of the literature data, it became evident that there might have been mixed phases present. The methods reported for obtaining other polymorphs relied solely on the use of alcohols or acetone, and some of these combinations resulted in the formation of solvates in our study. The process of desolvating these solvates under uncontrolled conditions frequently led to the presence of phase mixtures and partial hydration. In light of these findings and to continue with the in-house nomenclature started by Kuhnert-Brandstätter (**AH-I** to **AH-III**), we renamed the Alberola form III^54^ to **AH-IV** and designated our newly discovered polymorph as **AH-V**.

#### 3.2.3 Systematic Desolvation Studies

The fact that SGD can form solvates and hydrates opens up new routes for exploring solid-form screening conditions, particularly in the context of systematic desolvation studies. This approach has already yielded promising results in generating novel forms in several of our previous studies.^12,13^ Eight solvate forms and **Hy1-I** were stored under carefully controlled temperature and RH conditions, spanning from RT to 60 °C and from 0% to 100% RH (as detailed in Table 1). When solvated forms were stored at ambient conditions (RT and 50% RH), we observed a transformation into **Hy1-I** within 1 day for all solvates, except for **S-DMF** and **S-DMA**. After 1 week, **S-DMF** had completely transformed, while **S-DMA** still coexisted in a mixture (solvate and hydrate). Increasing the RH from 50% to 75% (or 100%) induced the transformation into **Hy1-I** for all solvates, regardless of whether they were stored at RT or 40 °C. Notably, **Hy1-I** itself did not undergo any phase changes when exposed to moisture at either RT or 40 °C.

**Table 1:** Systematic Desolvation Studies of SGD Solvates and **Hy1-I** after Storing the Forms for a Duration of 2 Weeks^a^

	conditions (*T* [°C]/RH [%])				
starting form	25/0	25/50	25/100	40/75	60/12
**Hy1-I**	AH-II	Hy1-I	Hy1-I	Hy1-I	AH-I + AH-II
**S-ACO**	AH-II	Hy1-I	Hy1-I	Hy1-I	AH-I + AH-II
**S-THF**	AH-V	Hy1-I	Hy1-I	Hy1-I	AH-I
**S-*t*BuOH**	AH-V	Hy1-I	Hy1-I	Hy1-I	AH-I
**S-MeOH**	AH-V	Hy1-I	Hy1-I	Hy1-I	AH-I
**S-CHXO**	S-CHXO	Hy1-I	Hy1-I	Hy1-I	AH-I + AH-II
**S-DMSO**	S-DMSO	Hy1-I	Hy1-I	Hy1-I	S-DMSO + AH-I
**S-DMF**	S-DMF	Hy1-I	Hy1-I	Hy1-I	AH-I
**S-DMA**	S-DMA	S-DMA + Hy1-I	Hy1-I	Hy1-I	AH-I + AH-II

a AH, anhydrate; Hy1-I, monohydrate I; S, solvate.

Storing the several solvates under the driest conditions possible (0% RH, over P_2_O_5_) yielded in **AH-II** or **AH-V**. **AH-II** was obtained through the desolvation of **Hy1-I** and **S-ACO**, while **AH-V** resulted from **S-THF**, **S-MeOH**, and **S-*t*BuOH**. The remaining four solvates (**S-CHXO**, **S-DMSO**, **S-DMF**, and **S-DMA**) proved highly stable at 0% RH and RT for up to 6 weeks, showing no transformation during this period (end of investigation time). However, when the temperature was raised from 25 to 60 °C, albeit also increasing the RH, it accelerated the desolvation process and led to different outcomes. The measurements taken after 1 day showed that no **AH-V** was present. Instead, all solvates had, to varying extents, undergone transition into either **AH-I** or a mixture of **AH-I** and **AH-II**. Extended storage of these mixtures of **AH-I** and **AH-II** at 60 °C did not alter the phase composition.

#### 3.2.4 Recrystallization from the Melt

**Figure 3:**
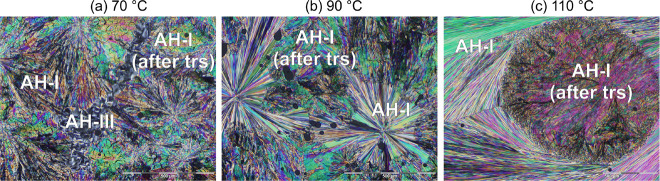
Melt annealing experiments of SGD at various temperatures, revealing the morphological differences between **AH-I** obtained directly from the melt and after phase transformation (trs) of **AH-III**/**AH-II** → **AH-I**.

To ensure the absence of any residual seed crystals of the anhydrate, **AH-I** was melted on a hot-bench and maintained at 210 °C for 30 s. Subsequently, the melt underwent annealing at temperatures of 70, 90, 110, and 130 °C. When the SGD melt was annealed for 60 min at 70 °C, three distinct crystal forms emerged: **AH-I**, **AH-II**, and **AH-III**, with melting points of 143–145 °C (**AH-III**), 175–176 °C (**AH-II**), and 189–190 °C (**AH-I**). Figure 3a shows a microscopic image of **AH-III** and **AH-I** obtained from the melt or after **AH-III** to **AH-I** phase transformation. Increasing the annealing temperature to 90 or 110 °C also led to the formation of all three polymorphs; **AH-III** was observed but quickly transformed into **AH-I** (Figure 3b,c). Further increasing the annealing temperature to 130 °C resulted predominantly in the formation of **AH-II** and **AH-I**, with **AH-II** transforming into **AH-I** at temperatures >155 °C.

### 3.3 SGD Monohydrates

#### 3.3.1 Structure Comparison and Pairwise Energy Calculations

Three distinct hydrate structures are reported in the Cambridge Structural Database (CSD),^87^ comprising one *P*2_1_ and two *P*2_1_/*c* structures. The latter two among these, namely, SOGUAN01^58^ and SOGUAN20,^88^ share identical packing arrangements, but the SGD molecule adopts different tautomeric forms. Given that the later structure determination (SOGUAN20) includes H atom positions, it was chosen as the basis for our structural discussion (**Hy1-I**). The second hydrate is referred to as **Hy1-II** from here onward.

Both hydrate structures exhibit almost identical conformations, differing by less than 2° in φ_1_ and φ_2_. Notably, **Hy1-I**, owing to its *P*2_1_/*c* space group symmetry, accommodates the two inversion-related conformers within its unit cell, while **Hy1-II** (*P*2_1_) contains only one of the two.

**Figure 4:**
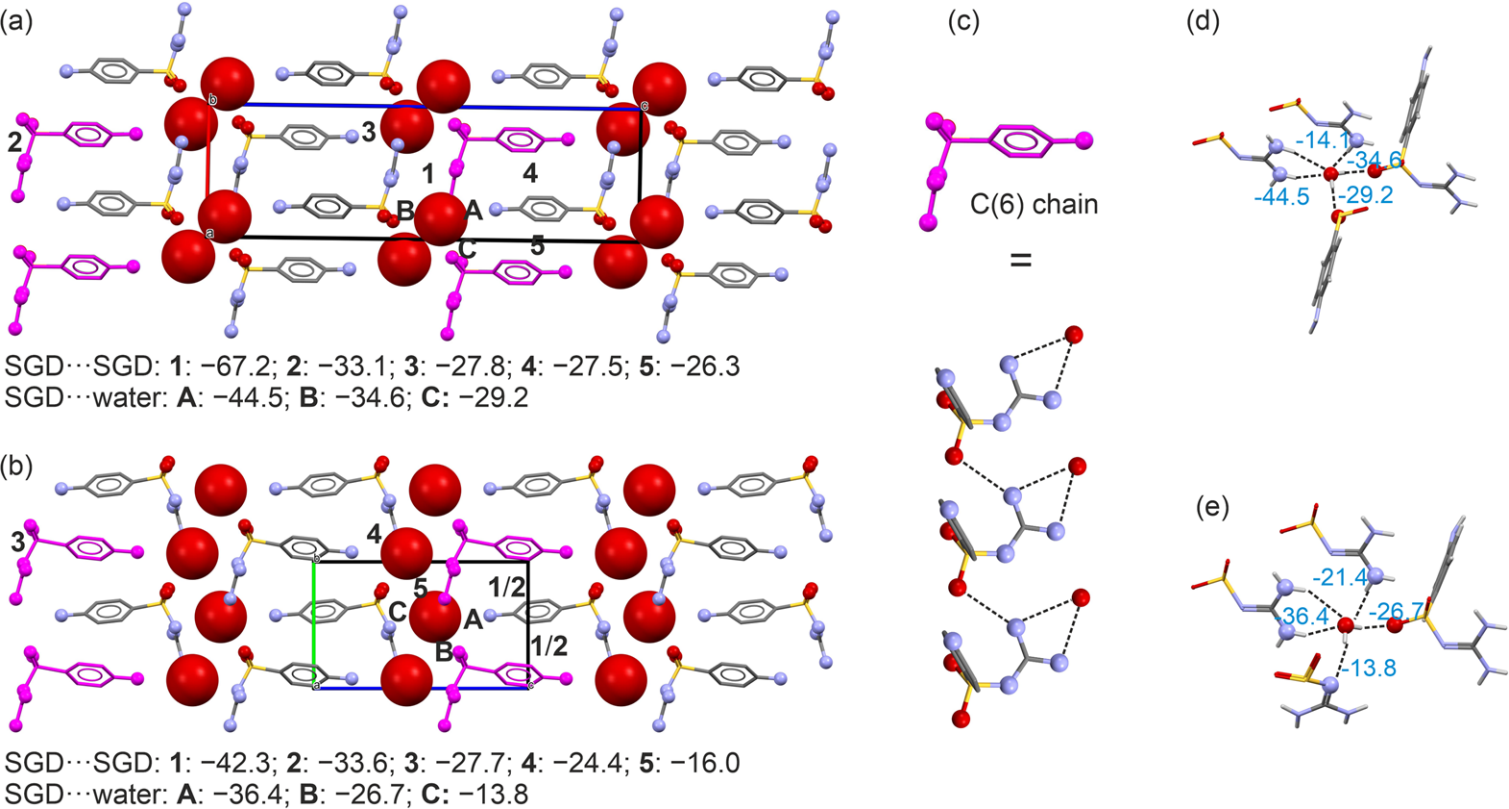
Packing comparison of (a) **Hy1-I** and (b) **Hy1-II**; the shared 2D-1 motif is highlighted in magenta. The numbers represent the pairwise intermolecular interaction energies (in kJ mol^–1^). (c) C(6) chain shared between the hydrate polymorphs. The connectivity of water molecules is depicted (d) for **Hy1-I** and (e) for **Hy1-II**. To enhance clarity, hydrogen atoms are not shown in panels a–c. Dashed lines in panel c indicate H-bonding interactions.

An analysis comparing the packing of the two hydrate structures showed a remarkable degree of similarity between these two polymorphs (Figure 4a,b). Specifically, there is a 2-dimensional (2D) packing similarity based on identical H-bonding between SGD and water molecules, with the SGD molecules forming C(6) chains.^89^ These C(6) chains are connected through a N–H···O bond, while the water molecule engages in two N–H···O_water_ interactions (Figure 4c) with C(6). The water interaction observed in this shared fragment was identified as the strongest water interaction, contributing −44.5 kJ mol^–1^ (for **Hy1-I**) and −36.4 kJ mol^–1^ (for **Hy1-II**) in intermolecular energy, as illustrated in Figure 4d,e, respectively.

The C(6) chains are linked to adjacent C(6) chains through O_water_–H···O hydrogen bonds, forming a common 2D fragment, referred to as 2D-1. The key distinction between the two hydrate structures lies in the packing of these 2D-1 fragments. In **Hy1-II**, adjacent 2D-1 motifs are related by a 2_1_ symmetry and translation, while in **Hy1-I** the relation occurs by inversion symmetry. Furthermore, the two structures share the characteristics of water molecules forming five strong H-bonding interactions, contributing to the high stability of the hydrates. Remarkably, even after being stored for over 60 years under standard conditions, **Hy1-I** maintained its hydrate structure without displaying any indications of transforming into an anhydrous form.

#### 3.3.2 Thermal Analysis of **Hy1-I** and Temperature Dependent FT-IR Spectroscopy

**Figure 5:**
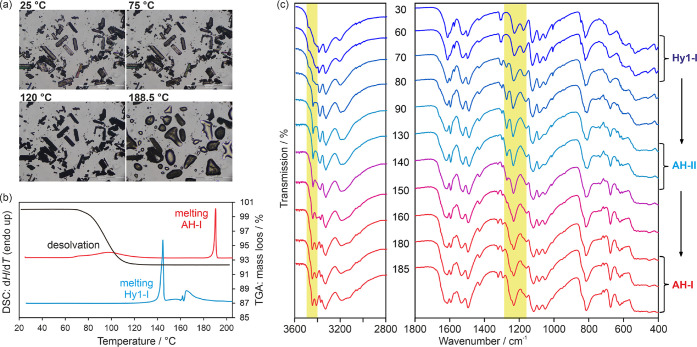
(a) Hot-stage microscopic investigations of **Hy1-I** (dry preparation). (b) TGA and DSC traces of **Hy1-I**: The TGA (black) and DSC data (red, upper curve, pierced lid) were measured at a heating rate of 5 °C min^–1^. In contrast, the DSC measurement with a hermetically sealed crucible (blue, lower curve) was performed at a rate of 10 °C min^–1^. (c) Temperature-dependent IR measurements following the dehydration of **Hy1-I**. The numbers indicate the measurement temperatures (°C), and distinctive regions are highlighted.

Our investigations yielded only one of the two SG hydrates, **Hy1-I**, and therefore, we focused our temperature-dependent studies solely on this hydrate phase. HSM observations (Figure 5a) revealed the typical behavior of a stoichiometric hydrate, characterized by “pseudomorphosis”^86^ as the temperature increased. Dehydration commenced at approximately 65 °C and was completed at around 130 °C. Further heating led to the melting of SGD in the range of 188.5–189 °C, which corresponds to the **AH-I** melting point.

These findings were corroborated with DSC and TGA investigations (Figure 5b). The broad endothermic peak observed in the DSC heating curve (red, upper DSC curve), corresponds to the dehydration reaction, as indicated by the loss of one mole of water per mole of SGD in the TGA curve. The second endothermic peak, occurring at 188.6 ± 0.3 °C, corresponds to the melting of **AH-I**. Notably, the hydrate’s melting point was measured at 143.0 ± 0.1 °C (blue, lower curve).

Temperature-dependent IR spectroscopy was used to track the dehydration process. The temperature was increased at a rate of 2 °C min^–1^, which was slower compared to the DSC experiments. This slower heating rate was employed to investigate whether **AH-II** formed before **AH-I**, as previously reported.^53^ By reducing the heating rate, we were able to complete the dehydration at a lower temperature of 90 °C, as opposed to the 130 °C required in the DSC experiments. Initially, the IR spectra of **Hy1-I** displayed no changes up to 60 °C. However, between 60 and 80 °C, a gradual emergence of new bands was observed (Figure 5c). These new bands indicate the formation of **AH-II**. Furthermore, in the temperature range between 130 and 160 °C, the phase transformation from **AH-II** to **AH-I** was observed. Hence, it is confirmed that the transition pathway is influenced by the dehydration temperature.

#### 3.3.3 Computationally Generated Monohydrate Crystal Energy Landscape

**Figure 6:**
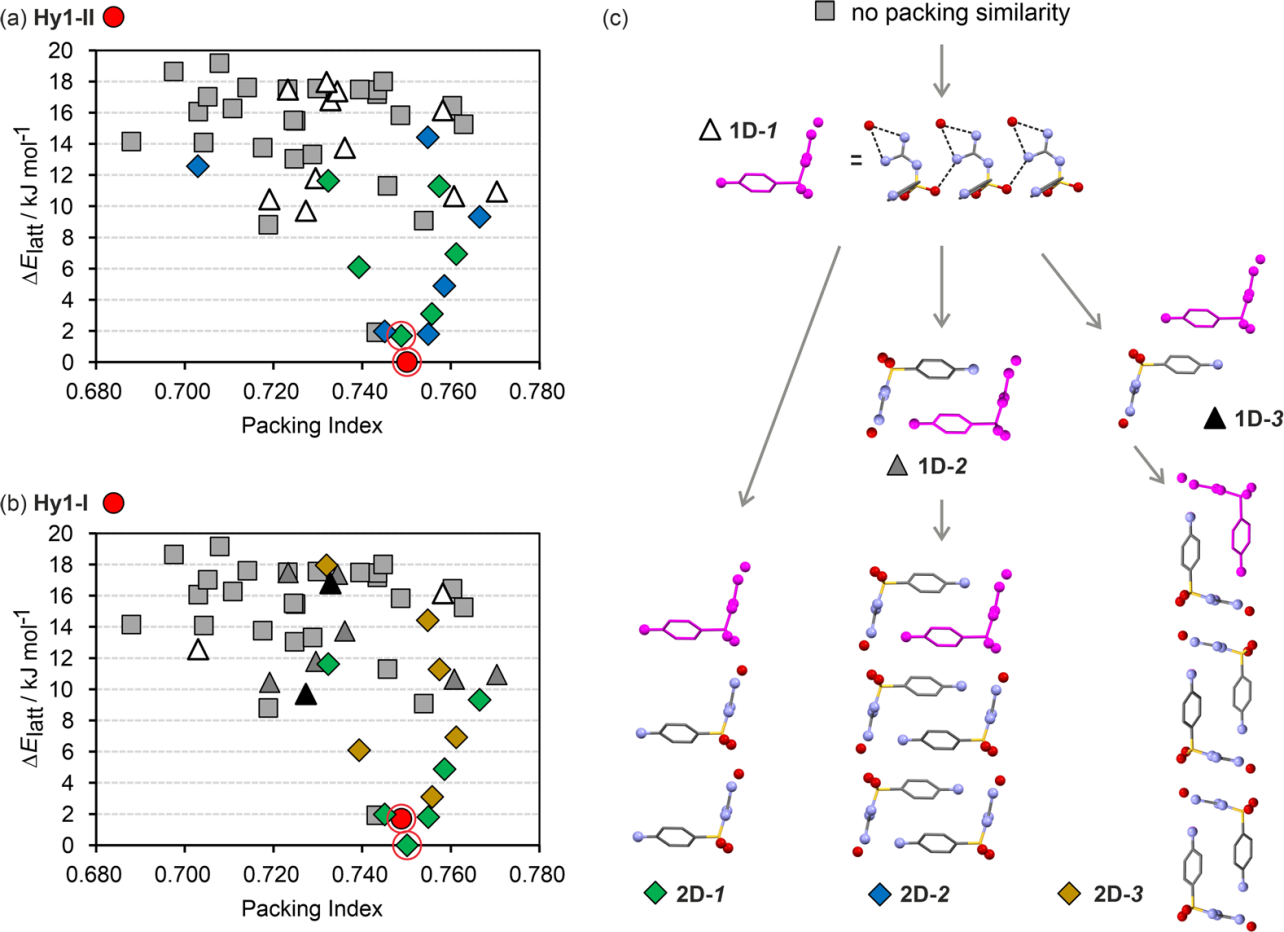
Computational generation of the SGD monohydrate crystal energy landscape. The lattice energy landscapes were clustered based on their similarity to **Hy1-II** (a) and **Hy1-I** (b), with packing fragments indicated in panel c. The terms “1D” and “2D” refer to one-dimensional and two-dimensional packing similarity, respectively.

The experimental investigations of **Hy1-I** were complemented by a virtual monohydrate screen, specifically, a CSP study. The energy landscape of monohydrate crystals exhibited a multitude of structures within the energy range expected for polymorphism.^90^ In total, we identified 50 structures within 20 kJ mol^–1^ and seven structures within 5 kJ mol^–1^ of the global minimum (Figure 6).

Encouragingly, the experimental hydrate structures were found as the global minimum and the second lowest energy structure, with **Hy1-II** corresponding to the global minimum. At the PBE-MBD* level of theory, these two structures were estimated to differ by only 1.7 kJ mol^–1^ in lattice energy, while the structures ranking second to fourth were within a mere 0.3 kJ mol^–1^ range. Driven by the structural similarity between the two hydrate polymorphs (Figure 4), a comparison and clustering analysis of the 50 lowest energy structures in relation to **Hy1-II** (Figure 6a) and **Hy1-I** (Figure 6b) was conducted.

The packing comparisons revealed that 24 out of the 50 structures share the 1D-1 chain motif (Figure 6c) observed in the experimental packings. Among the low energy structures (12 kJ mol^–1^ range), only 20% of them did not exhibit the 1D-1 motif. Therefore, it appears that the 1D-1 motif is the preferred packing characteristic in hydrate structures. However, it is also worth noting that the fourth ranked structure, although very low in energy, deviates from the 1D-1-based pattern. This structure may be considered a potential SGD hydrate polymorph, but its formation would likely require the disruption of the strong SGD···water interaction, which could already be present in solution.

The comparison of **Hy1-II** with other hypothetical structures reveals that most of the lowest energy structures not only share the 1D-1 chain fragment but also exhibit a 2D packing similarity (Figure 6a), specifically motifs 2D-1 or 2D-2 (Figure 6c). This similarity extends to polymorph **Hy1-I** as well. Similar to **Hy1-II**, **Hy1-I** also exhibits a 2D packing similarity with the majority of lowest energy structures (Figure 6b), packing motifs 2D-1 and 2D-3.

The imino version of **Hy1-I** was initially also chosen as an input structure for the PBE-MBE* optimizations. During the minimization process the relevant proton underwent relocation, ultimately yielding the amino version of the structure, namely, **Hy1-I**. Therefore, it appears that the amino tautomer represents the correct solution for the **Hy1-I** structure.

### 3.4 SGD Solvates

The experimental screen resulted in nine SGD solvate forms, i.e., from ACO, CHXO, MeOH, EtOH, *t*BuOH, THF, DMSO, DMF, and DMA (Figure 1).

#### 3.4.1 Crystal Structures and Pairwise Intermolecular Energy Calculations

Seven out of the nine solvate structures were solved from PXRD data (Table 2). The unit cell parameters for **S-ACO** have already been reported and agree with our redetermination, although **S-ACO** was found to adopt the *Pbca* space group symmetry and not *Pbcm*.^54^ For more information, see Table 2 and ESI, Table S2.

**Table 2:** Crystallographic Information of the SGD Solvates^a^

	**S-THF**	**S-DMSO**	**S-DMA**	**S-DMF**
chemical formula	C_7_H_10_N_4_O_2_S·C_4_H_8_O	C_7_H_10_N_4_O_2_S·C_2_H_6_OS	C_7_H_10_N_4_O_2_S·C_4_H_9_NO	C_7_H_10_N_4_O_2_S·C_3_H_7_NO
molar mass/g mol^–1^	286.35	292.38	301.37	287.35
crystal system	orthorhombic	orthorhombic	orthorhombic	monoclinic
space group	*P*2_1_2_1_2_1_	*P*2_1_2_1_2_1_	*P*2_1_2_1_2_1_	*P*2_1_
*a*/Å	9.8324(<1)	10.0367(<1)	9.6441(1)	7.0231 (<1)
*b*/Å	11.1144(1)	10.8651(1)	10.5177(1)	12.1415(2)
*c*/Å	13.1626(1)	13.2347(1)	31.3681(3)	8.2156(1)
α/°	90	90	90	90
β/°	90	90	90	94.249(1)
γ/°	90	90	90	90
vol/Å^3^	1438.42(2)	1443.24(2)	3181.81(6)	698.63(2)
*Z*	4	4	8	2

	**S-MeOH**	**S-***t***BuOH**	**S-ACO**
chemical formula	C7H10N4O2S·C4H9NO	C_7_H_10_N_4_O_2_S·C_4_H_10_O	2(C_7_H_10_N_4_O_2_S)·C_3_H_6_O
molar mass/g mol^–1^	301.37	288.37	486.58
crystal system	orthorhombic	monoclinic	orthorhombic
space group	*Pbca*	*P*2_1_/*c*	*Pbca*
*a*/Å	12.0197(2)	17.7340(2)	12.6609(1)
*b*/Å	6.9858(1)	6.8737(<1)	18.7414(1)
*c*/Å	27.2785(4)	12.2851(1)	19.2784(2)
α/°	90	90	90
β/°	90	93.732(<1)	90
γ/°	90	90	90
vol/Å^3^	2290.50(6)	1494.37(2)	4574.46(7)
*Z*	8	4	8

a For more details, please refer to ESI, Tables S10 and S11.

**Figure 7:**
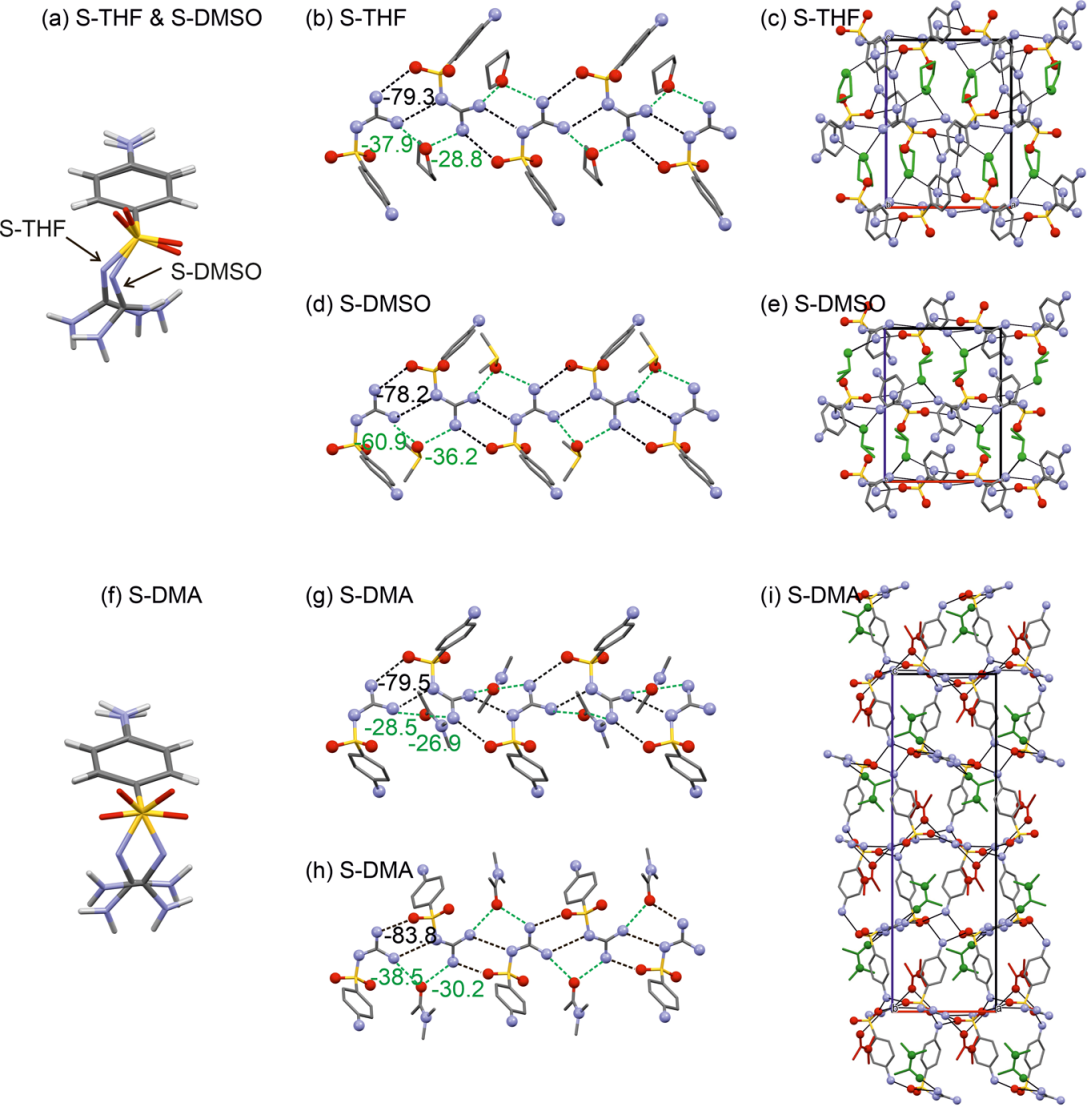
(a) Conformational overlay of SGD in **S-THF** and **S-DMSO**. (b) Depiction of H-bonding motifs and (c) packing diagram for **S-THF**. (d) H-bonding motifs and (e) packing diagram for **S-DMSO**. (f) Conformational overlay of the SGD molecules in **S-DMA**. (g, h) H-bonding motifs and (i) packing diagram for **S-DMA**. H atoms have been omitted for clarity, solvent molecules are drawn in green, and selected strong H-bonding interactions are indicated with dashed lines. Given numbers in panels b, d, g, and h correspond to pairwise interaction energies in kJ mol^–1^.

Three solvates, namely **S-THF**, **S-DMSO**, and **S-DMA**, crystallize in the orthorhombic space group *P*2_1_2_1_2_1_. The lattice parameters for **S-THF** and **S-DMSO** are closely related, with each asymmetric unit containing one SGD molecule and one solvent molecule. The observed SGD conformations in these two solvates differ by approximately 15° in φ_2_ and less than 2° in φ_1_, as depicted in Figure 7a. A structural analysis revealed that they are isostructural. The strongest H-bonding interaction observed in **S-THF** is a corrugated double-chain motif (Figure 7b), mediated by C(4) and C(6) chains, forming a strong *R*_2_^2^(8) ring motif. The latter was identified as the strongest interaction, contributing approximately −79.3 kJ mol^–1^ in pairwise energy. The THF molecule is linked to the double-chain motif through two strong N–H···O_THF_ interactions, accounting for −37.9 and −28.8 kJ mol^–1^ in energy (see ESI, Table S12). A strong N–H···O interaction (−39.3 kJ mol^–1^) connects the corrugated double-chain fragments along the crystallographic *b*-axis. Another strong N–H···N interaction interconnects the double-chain motifs along the crystallographic *c*-axis, resulting in a H-bonded 3D network structure (Figure 7c).

In the isostructural **S-DMSO** solvate, the same strong intramolecular interactions are observed. Notably, the DMSO O atom occupies the position previously held by the THF O atom. The most significant disparity between the two structures lies in the strength of the SGD···solvent interactions, which are stronger in **S-DMSO** than in **S-THF** (Figure 7d). The strongest SGD···DMSO interaction accounts for −60.9 kJ mol^–1^ (see ESI, Table S13).

The third *P*2_1_2_1_2_1_ solvate, **S-DMA**, distinguishes itself from **S-THF** and **S-DMSO** due to its doubled *c*-axis, resulting in a *Z*′ = 2 structure. In **S-DMA**, the two SGD conformations differ; one aligns with conformational region A, the other with region B. These two conformations can be linked through mirror symmetry, as depicted in Figure 7f. Similar to **S-THF** and **S-DMSO**, **S-DMA** also features the strong H-bonded double-chain motif. In this case, each of the symmetry-independent SGD molecules forms a distinct motif of this type, as shown in Figure 7g,h. These two double-chain motifs interact through four strong N–H···O interactions, facilitated by the aniline H-donor groups. Moreover, the solvent molecule also plays a crucial role in stabilizing the **S-DMA** structure.

**Figure 8:**
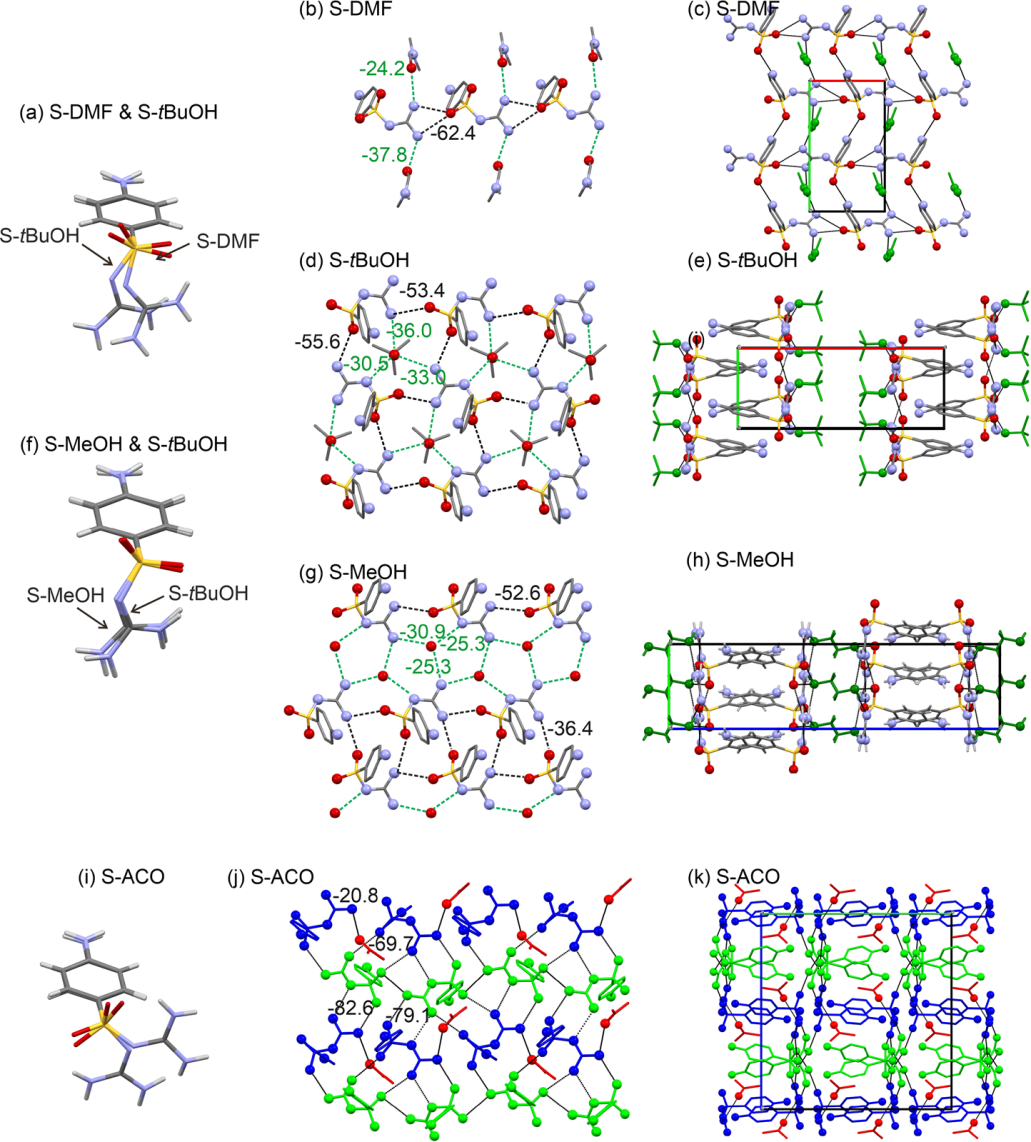
(a) Conformational overlay of SGD in **S-DMF** and **S-*t*BuOH**. (b) Depiction of H-bonding motifs and (c) packing diagram for **S-DMF**. (d) H-bonding motifs and (e) packing diagram for **S-*t*BuOH**. (f) Conformational overlay of SGD in **S-MeOH** and **S-*t*BuOH**. (g) H-bonding motifs and (h) packing diagram for **S-MeOH**. (i) Conformational overlay of the SGD molecules in **S-ACO**. (j) H-bonding motifs and (k) packing diagram for **S-ACO**. H atoms have been omitted for clarity, solvent molecules are drawn in green in panels c, e, and h, and symmetry independent molecules are color coded in panels j and k. Selected strong H-bonding interactions are indicated with dashed lines. Numbers given in panels b, d, g, and j denote pairwise intermolecular energies (in kJ mol^–1^).

**S-DMF** crystallizes in the monoclinic space group *P*2_1_, with one SGD and one DMF molecule in the asymmetric unit (Figure 8a). In contrast to the corrugated double-chain motif observed in the preceding three solvate structures, **S-DMF** exhibits a distinct chain motif. This motif consists of two C(6) chains, formed through N–H···O interactions between the guanidinium moiety and one SO_2_ oxygen atom. These chains propagate along the crystallographic *a*-axis and contribute significantly to the structure’s stability, with a pairwise energy of −62.4 kJ mol^–1^ (Figure 8b, ESI Table S15). The second strongest interaction (N–H···O) connects adjacent chain motifs (Figure 8c), mediated by 2_1_-fold symmetry. The DMF molecule itself engages in strong H-bonding interactions (N–H···O_DMF_) with SGD, as illustrated in Figure 8b. Interestingly, the second strongest SGD···DMF interaction is not the second N–H···O_DMF_ interaction, but a C–H···π close contact.

The **S-***t***BuOH**, a monoclinic *P*2_1_/*c* structure, has one SGD and one solvent molecule in the asymmetric unit. The SGD conformation in **S-***t***BuOH** is related to the conformation seen in the other solvates (Figure 8a). Each of the SO_2_ oxygen atoms engages in a strong C(6) chain motif with a guanidinium NH_2_ group, forming N–H···O interactions with energies of −55.6 and −53.4 kJ mol^–1^. The two interaction patterns result in the formation of a 2D layer of SGD molecules (Figure 8d) situated on planes that are parallel to (100). The *t*BuOH molecule forms three strong H-bonds with SGD, one O–H···N and two N–H···O, effectively linking the solvent molecule to three SGD molecules (Figure 8c). Interestingly, the strongest **S-*t*BuOH** interaction is not a H-bonding interaction but rather an aromatic π···π close contact between SGD molecules (−66.0 kJ mol^–1^, see ESI, Table S17). The **S-*t*BuOH** structure can be described as a layer structure (Figure 8e), with alternating layers of SGD and *t*BuOH parallel to (100).

The second alcohol solvate, **S-MeOH**, adopts the orthorhombic *Pbca* space group and has one SGD and one MeOH molecule in its asymmetric unit. The conformation of SGD in **S-MeOH** closely resembles that observed in **S-*t*BuOH** (Figure 8f). Moreover, these two solvates share identical chains of SGD molecules [C(6)], which extend parallel to the respective crystallographic *b*-axes of the two alcohol solvates. This strong H-bonding interaction (N–H···O, –52.6 kJ mol^–1^) contributes significantly to the lattice energy of **S-MeOH** (Figure 8g). The positions of the alcohol molecules are consistent between the two solvates. In **S-MeOH**, three H-bonding interactions involving MeOH are observed: one O–H···N and two N–H···O interactions (ESI, Table S16). These interactions are weaker when compared to those found in **S-*t*BuOH**. The linkage between adjacent C(6) chains and solvent molecules differs in the two solvates, resulting in two distinct solvate structures (Figure 8h,k).

**S-ACO** stands out as the sole solvate crystallizing in a 2:1 ratio. Its crystal structure, orthorhombic symmetry and *Pbca* space group, features two SGD molecules and one ACO molecule within its asymmetric unit. Notably, the conformations of these two independent molecules exhibit substantial differences (Figure 8i) and can be grouped into two conformational regions (A and B, Figure 2). To transition from one conformation to the other, a 180° rotation of the guanidine function (ϕ_1_) is necessary, along with a relatively minor adjustment in ϕ_2_, of approximately 12°. The strongest intermolecular interactions within **S-ACO** arise from interactions between the two independent SGD molecules (Figure 8j). Notably, there are two strong *R*_2_^2^(8) ring motifs, each comprising a N–H···N and N–H···O interaction, with energies of −82.6 and −71.9 kJ mol^–1^, respectively (ESI, Table S18). The third strongest interaction is a *R*_2_^2^(12) motif, contributing −69.7 kJ mol^–1^ in pairwise energy. These strong interactions are all found within planes parallel to (010). Intriguingly, while the ACO molecule participates in a classical H-bonding interaction with one of the SGD molecules (N–H···O, –20.8 kJ mol^–1^), this is not the most energetically favorable SGD···ACO interaction. ACO additionally engages in close contacts and C–H···π interactions with the second SGD molecule (−27.4 kJ mol^–1^ and −21.4 kJ mol^–1^).

When comparing the crystal structures of **S-ACO** (Figure 8k), **S-DMF**, **S-MeOH**, and **S-*t*BuOH** to those of **S-THF**/**S-DMSO** and **S-DMA**, it becomes apparent that they exhibit distinct H-bonding and distinct packing motifs.

Comparisons of the solvate structures with the anhydrates (section 3.5.1) reveal a 2D packing similarity between **S-THF** and **S-DMSO** with **AH-V**, providing a rationale for the desolvation of **S-THF** to **AH-V** at 0% RH (RT). In the case of **S-DMSO**, challenges in desolvating the solvate at RT (0% RH) were encountered within the allotted investigation time. Only by increasing the temperature were we able to successfully desolvate **S-DMSO** into an anhydrous SGD form (**AH-I**). A similar temperature-induced desolvation also led to **AH-I** in the case of **S-THF**.

Among the other five structurally characterized solvates, only 1D similarity was observed (identical chains allowing the rotation of the aniline moiety) with SGD anhydrate polymorphs. **S-DMF** exhibits 1D packing similarity with **AH-I** and **AH-V**, while the alcohol solvates exhibit 1D packing similarity with **AH-I**, and the remaining two solvates (**S-ACO** and **S-DMA**) with **AH-V**. These 1D similarities do not offer a satisfactory explanation for the observed dehydration pathways.

#### 3.4.2 Thermal Analysis and Temperature-Dependent IR Spectroscopy

**S-EtOH** (which was not obtained phase pure) and **S-MeOH** were observed to be highly unstable under ambient conditions. They started to transform into **Hy1-I** within a matter of seconds to minutes. Therefore, the DSC, TGA, and IR data for these two solvates are not reported in this work. DSC measurements were conducted on SGD solvates in hermetically sealed and pinholed pans, aiming to assess the influence of atmospheric conditions on the desolvation behavior and to identify the associated processes.

**Figure 9:**
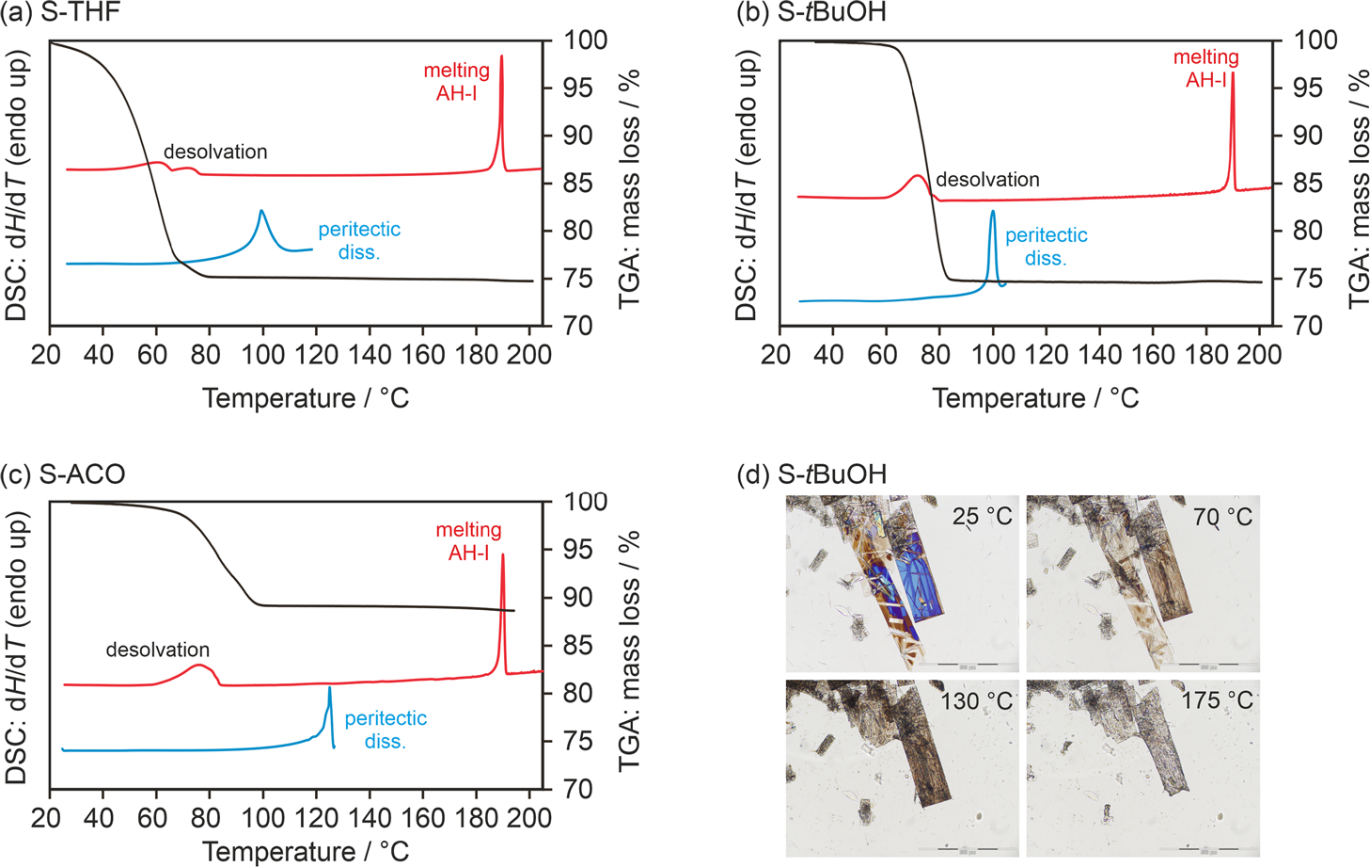
DSC and TGA traces of (a) **S-THF**, (b) **S-*t*BuOH**, and (d) **S-ACO**. TGA curves are depicted in black, pierced DSC pan measurements in red (top curves, heating rate of 5 °C min^–1^), and DSC measurements in hermetically sealed pans in blue (bottom curves, heating rate of 10 °C min^–1^). (d) Hot-stage microscopy images recorded for **S-*t*BuOH** showing the desolvation process (dry preparation).

Three of the solvates, **S-THF**, **S-*t*BuOH**, and **S-ACO** exhibited similar behavior as temperature increased (Figure 9). In DSC measurements conducted with pierced crucibles, desolvation events were observed at temperatures ≤90 °C. As the temperature further increased, a single melting event was observed at 188.6 ± 0.3 °C, corresponding to the melting of **AH-I**. Modifying the experimental conditions, specifically by employing hermetically sealed DSC pans, enabled us to determine the dissociation temperatures for these three solvates, which were found to be 99.1 ± 0.8 °C for **S-THF** (Figure 9a), 97.2 ± 1.0 °C for **S-*t*BuOH** (Figure 9b), and 123.8 ± 0.9 °C for **S-ACO** (Figure 9c). The HSM investigation for **S-*t*BuOH** is shown in Figure 9d as an example for this group of solvates. With increasing temperature, the solvate crystals become opaque, and pseudomorphosis can be seen. The mass loss recorded in the TGA traces corroborates the desolvation temperatures and stoichiometric ratios established from the crystal structures. Specifically, **S-THF** and **S-*t*BuOH** were identified as monosolvates, while **S-ACO** was confirmed as a hemisolvate.

Complementary IR heating experiments, revealed that upon desolvation, **AH-I** was not the sole polymorph observed. Specifically, in the case of **S-THF**, **AH-V** band positions were detected at 70 °C but disappeared upon further heating, indicating a transformation from **AH-V** to **AH-I** (ESI, Figure S32), likely induced by the presence of **AH-I** seed crystals, which formed during desolvation. Likewise, **S-*t*BuOH** exhibited **AH-II** band positions at 70 °C, which then transformed into **AH-I** with continued heating (ESI, Figure S36). Consequently, the desolvation temperature (and presence of seed crystals) plays a crucial role in determining which specific polymorph of SDG is obtained. It is worth noting that the IR-dependent studies were conducted with a heating rate of 2 °C min^–1^, whereas the initial DSC studies employed a faster heating rate of 5 °C min^–1^. The slower heating rates favor the formation of polymorphs other than **AH-I**.

**Figure 10:**
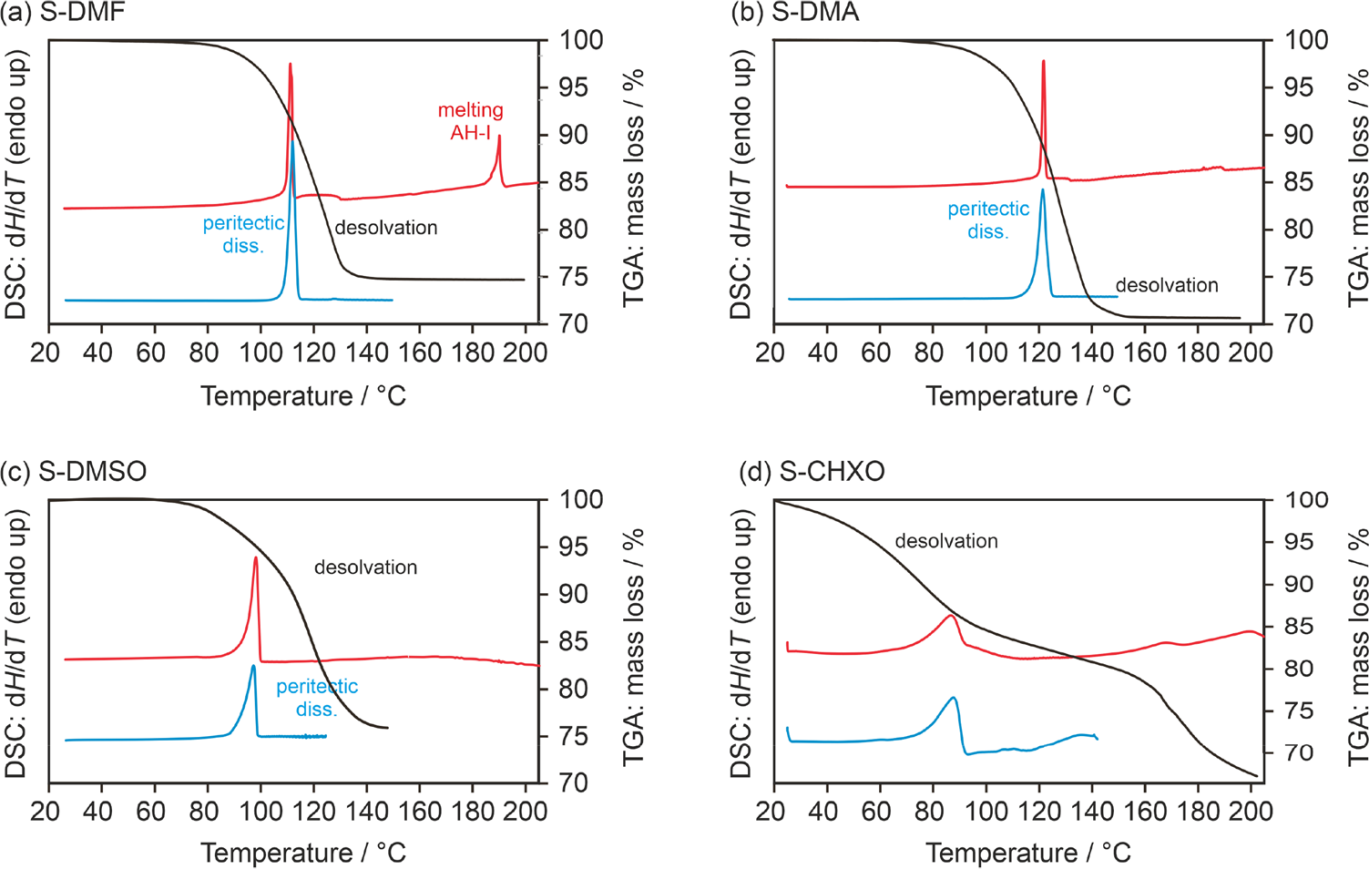
DSC and TGA traces of (a) **S-DMF**, (b) **S-DMA**, (c) **S-DMSO**, and (d) **S-CHXO**. TGA curves are depicted in black, pierced DSC pan measurements in red (top curves, heating rate of 5 °C min^–1^), and DSC measurements in hermetically sealed pans in blue (bottom curves, heating rate of 10 °C min^–1^).

In the case of **S-DMF**, **S-DMA**, and **S-DMSO**, the DSC traces, irrespective of the type of pans used, clearly indicated dissociation temperatures at 109.2 ± 0.8 °C for **S-DMF** (Figure 10a), 118.8 ± 1.1 °C for **S-DMA** (Figure 10b), and 95.3 ± 0.9 °C for **S-DMSO** (Figure 10c). Furthermore, the TGA curves confirmed a consistent 1:1 molar ratio between the SGD and the solvent for all three solvates. The TGA heating experiments concluded with the formation of **AH-I** (stopped at 160 °C, once the desolvation process was completed).

The TGA mass loss observed for **S-CHXO** corresponds to approximately 1 mol of CHXO per mole SGD (Figure 10d). When the TGA and DSC experiments were halted at 100 °C, it was evident that a yellow-brownish mass had formed (dissociation of the solvate). The presence of a solvate was indirectly confirmed through desolvation studies conducted at lower temperatures and the mass loss observed in the TGA experiments.

#### 3.4.3 Virtual Solvate Screening

In the context of virtual solvate screening, we tested the multicomponent hydrogen-bonding propensity tool, which is a quicker alternative compared to CSP. The CSP approach would require the generation of distinct multicomponent crystal energy landscapes, and furthermore, even different ratios of SGD/solvent. Since the importance of SGD···solvent H-bonding interactions has already been established, we selected the set of SGD solvates and hydrates as a benchmark for evaluating the solvate propensity predictions. However, it should be noted that H-bonding is not the exclusive driving force for solvate formation, thereby diminishing the predictability success of the selected approach.

**Figure 11:**
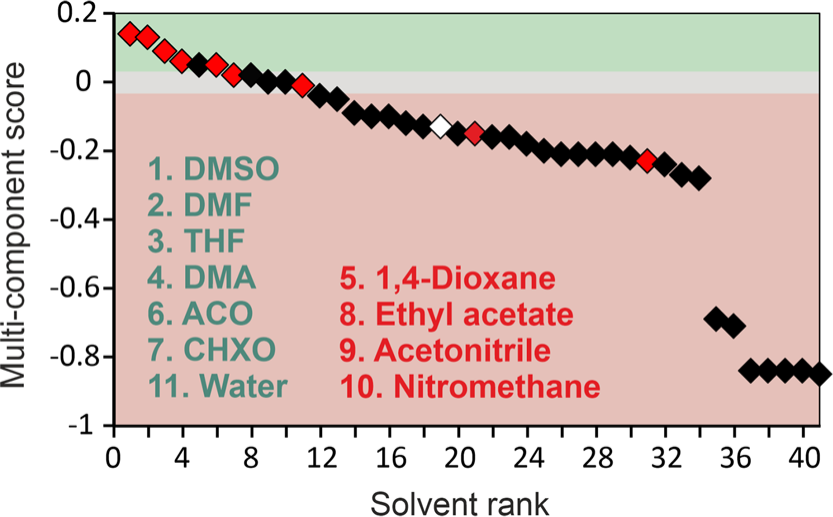
Multicomponent hydrogen-bond propensity chart for SGD. Experimentally observed solvates are indicated in red (fully characterized) and white (indication that solvate exists). Highest ranked solvents are listed in green for solvate forming solvents and in red for nonsolvate forming solvents.

Figure 11 illustrates the ranking of the 41 solvents against the multicomponent-score. A positive score indicates that a heteromeric interaction (SGD/solvent) is more likely than a homomeric interaction (SGD/SGD or solvent/solvent). The higher the score, the greater the tendency for solvate formation. Scores close to 0 do not strongly favor either type of interaction.

Out of the ten solvent molecules capable of forming solvates (hydrates), six were found in the range that favors solvate formation (DMSO, DMF, THF, DMA, ACO, and CHXO). Water falls in the neither-favoring-nor-opposing range. The only outliers were the alcohols (MeOH, EtOH, and *t*BuOH), which were identified in the range where the SGD/SGD interaction propensity surpasses the SGD/alcohol interaction propensity. It is worth noting that the SGD/SGD propensity alone is already quite high, making SGD/solvent propensity values well above the 0.8 value necessary to “predict” solvate formation. Moreover, the tool’s consideration of only one possibility, despite the capacity of alcohols to participate in multiple H-bonding interactions, may have contributed to the inaccurate propensity prediction result. Figure 11 additionally indicates that there are four solvent molecules with a high propensity for solvate formation, although this has not been experimentally confirmed. These solvents include 1,4-dioxane, ethyl acetate, acetonitrile, and nitromethane.

The seven solvent molecules found at the lower end of the range are predicted as very unlikely to form solvates and include dichloromethane, chloroform, and the five hydrocarbon-based solvents used. We do not rule out the possibility that other alcohol solvates, particularly transient solvates, may exist. This is especially relevant since sulfonamides are known to have a high propensity for forming solvates.^91^ Nevertheless, based on H-bond propensity calculations, a high success rate could have been achieved by using only the top-ranked solvents.

### 3.5 SGD Anhydrates

Four distinct polymorphs of SGD were identified (**AH-I**, **AH-II**, **AH-III**, **AH-V**) and for another anhydrate (**AH-IV**) lattice parameters^54^ have been reported. **AH-III** was found to be very unstable, and only a low quality PXRD pattern of an **AH-II**/**AH-III** mixture could be recorded (ESI, Figure S31) in addition to its melting point at 143–145 °C.

#### 3.5.1 Crystal Structures

The structures of **AH-I** and **AH-V** were determined from PXRD data and are discussed together with **AH-II**.^92^

**Figure 12:**
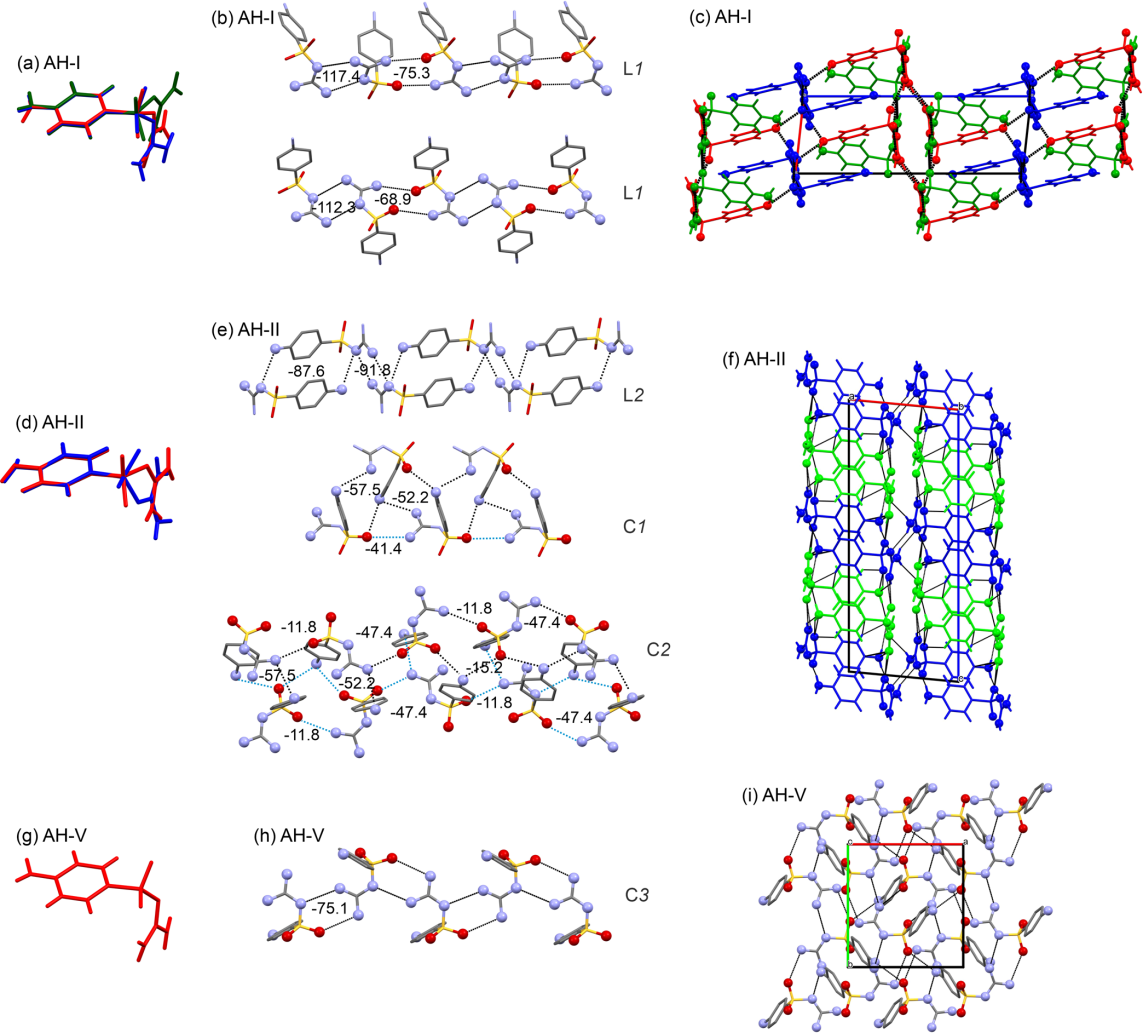
Crystal structure comparison. **AH-I**: (a) conformation overlay, (b) strongest H-bonded ladder motifs, and (c) packing viewed along the crystallographic *b*-axis. **AH-II**: (d) conformation overlay, (e) H-bonded chain motifs, and (f) packing viewed along the crystallographic *b*-axis. **AH-V**: (g) conformation, (h) H-bonded chain motif, and (i) packing viewed along the crystallographic *c*-axis.

**AH-I** crystallizes in the triclinic *P*1̅ space group, with three molecules in the asymmetric unit. These three molecules exhibit the same φ_1_ value (Figure 1). However, they span a range of 40° in φ_2_ (Figure 12a). The most prominent H-bonded motifs are two ladder motifs (L1), each composed of two strong *R*_2_^2^(8) and *R*_2_^2^(12) ring motifs, propagating along the crystallographic *b*-axis (Figure 12b). The first ladder motif comprises two symmetrically independent SGD molecules, with the two ring motifs contributing −117.4 kJ mol^–1^ [*R*_2_^2^(8))] and −75.3 kJ mol^–1^ [(*R*_2_^2^(12)] in pairwise energy. The *R*_2_^2^(8) motif is formed through two strong N–H···N interactions, and the *R*_2_^2^(12) motif comprises two N–H···O bonds. A similar connectivity is observed in the second ladder, but these ring motifs involve only the third SGD molecule. The two ladder motifs are connected through N–H···N and N–H···O interactions, resulting in a 3D H-bonded structure (Figure 12c).

**AH-II** exhibits monoclinic *P*2_1_/*c* symmetry and has two molecules within its asymmetric unit. These two molecules display distinct conformations (Figure 12d). One of the SGD molecules forms a unique ladder-like motif, referred to as L2. This motif hosts the two strongest pairwise intermolecular interactions observed in **AH-II** (−91.8 kJ mol^–1^ and −87.6 kJ mol^–1^, Figure 12e). Another distinctive 1D interlinked double-chain motif, C1, can be identified, which forms strong H-bonding interactions. Importantly, C1 serves as a bridge connecting the two symmetrically independent SGD molecules. These motifs, L2 and C1, propagate along the crystallographic axes *a* and *b*, respectively. Along the crystallographic *c*-axis, an additional interlinked H-bonded double chain motif, C2, becomes apparent. Collectively, the L2, C1, and C2 motifs contribute to the formation of a stable and intricately interconnected structure.

**AH-V** crystallizes in the monoclinic *P*2_1_/*n* space group with one molecule in the asymmetric unit (Figure 12g). The conformation in **AH-V** deviates by approximately 50° and 30° from the global gas-phase minimum. Each of the six N–H groups forms a strong intermolecular interaction with one of the two SO_2_ oxygen atoms or the −N= atom. Each of the purely H-bonding acceptor groups serves as an acceptor twice. One of the N–H groups forms a bifurcated inter- and intramolecular H-bonding interaction. In total, seven adjacent SGD molecules are linked through strong H-bonding interactions to one SGD molecule. The strongest intermolecular interaction is a *R*_2_^2^(8) dimer, involving the N–H···N and N–H···O H-bonds, contributing −75.1 kJ mol^–1^ in pairwise energy and forming the C3 motif along the crystallographic *c*-axis (Figure 12h). The second strongest interaction observed in **AH-V** is another *R*_2_^2^(8) dimer, once again formed by N–H···N and N–H···O H-bonds (−69.8 kJ mol^–1^), linking adjacent C3 motifs. The remaining three strong H-bonding interactions of C(6) or C(8) nature involve N–H···N or N–H···O bonds, leading to a well H-bonded structure (Figure 12i).

A common feature among all SGD structures (anhydrates, hydrates, and solvates) is a conformational energy difference of approximately 8–24 kJ mol^–1^ relative to the global minimum conformer. This energy disparity is compensated by the formation of strong H-bonding interactions (SGD···SGD and SGD···solvent), highlighting the significance of these interactions in stabilizing the various SGD solid-state forms. The experimental SDG conformations span the entire possible range in φ2 (0–360°). Surprisingly, the φ_1_ values are confined to the ranges of 78 ± 15° and 282 ± 15°, as illustrated in Figure S28 (ESI). Notably, none of the experimental structures exhibit the global minimum conformation, emphasizing the interplay between conformational and intermolecular energy contributions in the crystal structures. All identified SGD structures have been found to belong to a single tautomer, namely the amino form, while the imino tautomer has been observed in some metal complexes^88^ of SGD.

#### 3.5.2 Computationally Generated SGD Anhydrate Crystal Energy Landscape

**Figure 13:**
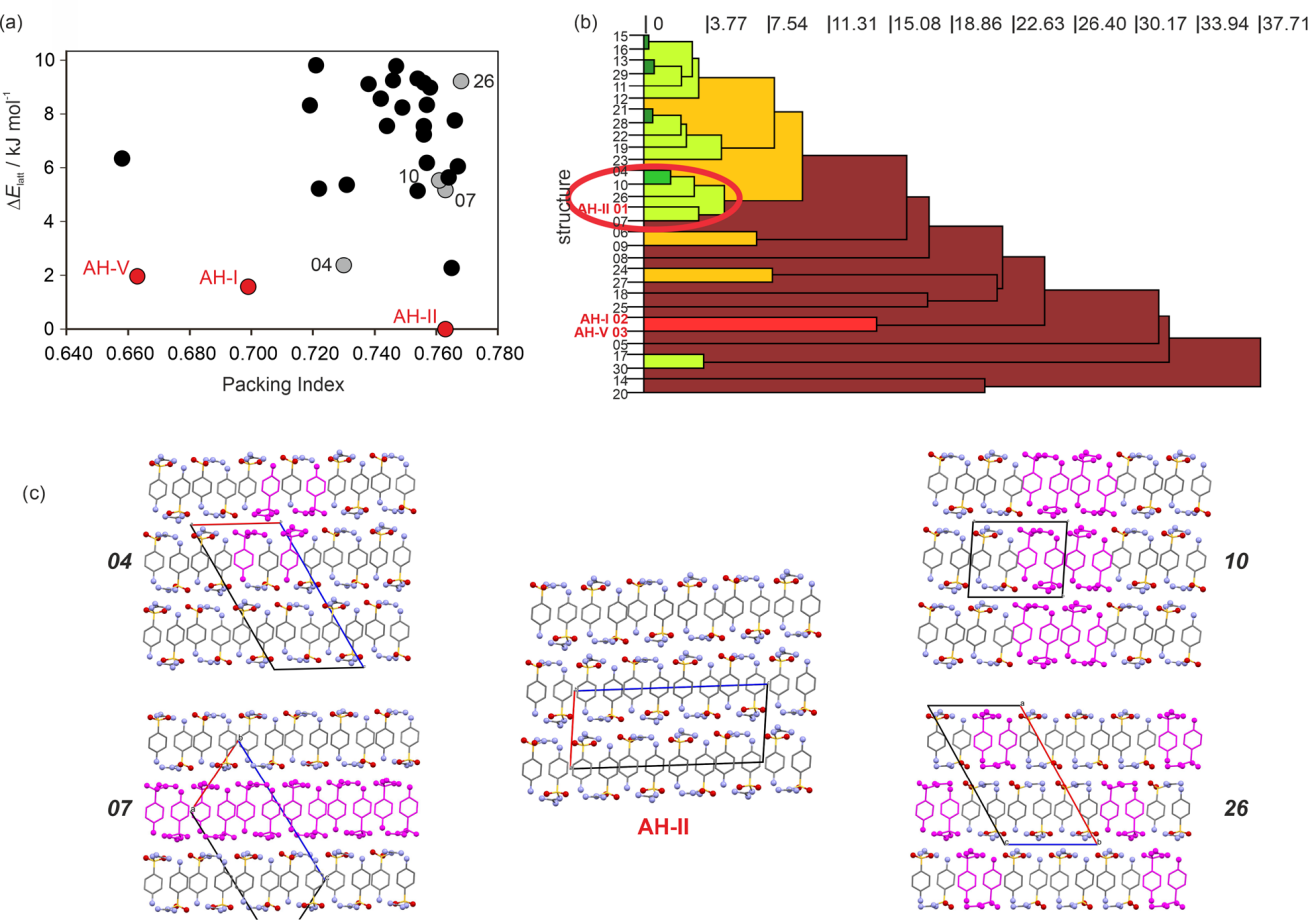
(a) Anhydrate lattice energy landscape with experimentally observed structures highlighted in red and potential **AH-IV** structures in gray. (b) Dendrogram showing the packing similarity of computed SGD structures. The horizontal axis corresponds to the PS_ab_ value (similarity).^93^ Dark green indicates almost identical packing, and dark red indicates dissimilar packing. (c) Packing diagrams of the **AH-II** cluster seen in panel b. Packing motifs seen in **AH-II** are highlighted in magenta in structures 04, 07, 10 and 26. An enlarged version of the packing diagrams is provided in ESI, Figure S6.

The SGD lattice energy landscape includes a total of 71 structures within a 15 kJ mol^–1^ range of the global minimum structure (ESI, Table S3). Figure 13a shows the lowest energy structures. **AH-I**, a *Z*′ = 3 structure, has been added to the lattice energy landscape. Remarkably, the three structurally characterized SGD anhydrate forms were found as the three lowest energy structures, with **AH-II** identified as the global minimum. **AH-I** was calculated to be approximately 1.6 kJ mol^–1^ less stable than **AH-II** and **AH-V** approximately 2 kJ mol^–1^ less stable than **AH-II**.

The structure of **AH-IV** remains elusive. Alberola and coauthors^54^ reported a *P*2_1_/*c* cell closely resembling the lattice parameters of **AH-II**, but with a lower density. Consequently, structure comparisons^93^ were conducted, identifying structures related to **AH-II** (Figure 13b). The packing diagrams of these related structures were examined in more detail (Figure 13c). Structures **AH-II** and 07, **AH-II** and 10, as well as **AH-II** and 26 exhibit 2D packing similarity. Furthermore, structure 04 and **AH-II**, show a 1D relationship involving double stacks of SGD molecules. Interestingly, all five **AH-II** cluster structures are *Z*′ = 2, with four of them (**AH-II**, 04, 07, and 26) sharing the same space group (*P*2_1_/*c*) and possessing similar lattice parameters. It is possible that one of these structures, or a combination thereof (involving disorder, stacking faults, or a polytypic structure), could correspond to **AH-IV**, with structure 04 being the most likely candidate for **AH-IV** based on energy and density considerations.

#### 3.5.3 Temperature-Dependent Stability of the SGD Anhydrate Polymorphs

**Figure 14:**
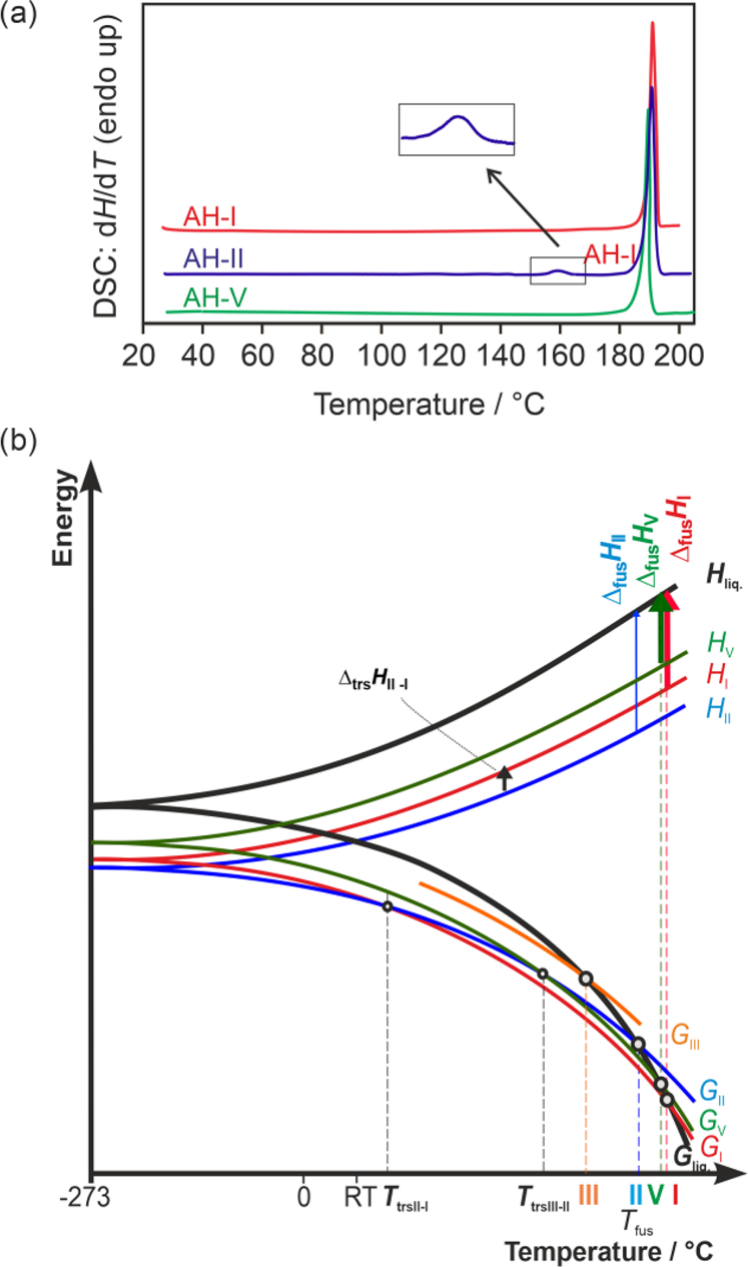
(a) DSC heating curves of SGD anhydrates **I**, **II**, and **V**. (b) Semischematic energy/temperature diagram. *T*_fus_, melting point; *G*, Gibbs free energy; *H*, enthalpy; Δ_fus_*H*, enthalpy of fusion; *T*_trs_, transition point; Δ_trs_*H*, transition enthalpy; liq, liquid phase (melt). The bold vertical arrows signify experimentally measured enthalpies.

The DSC heating curve for **AH-I** displays a single endothermic event at 188.6 ± 0.3 °C, corresponding to its melting process (Figure 14a). This melting event is associated with an enthalpy of fusion (Δ_fus_*H*) of 27.47 ± 0.05 kJ mol^–1^. When the melted sample was cooled to RT (at a rate of 5 or 10 °C min^–1^), no thermal event occurred. However, upon reheating the sample, a glass transition was observed at approximately 60 °C, followed by recrystallization starting from 110 °C onward. The phase obtained during recrystallization was **AH-I**.

In the case of the second polymorph, **AH-II**, two endothermic events were observed upon heating. The first event, occurring at temperatures >155 °C, represents the phase transformation from **AH-II** to **AH-I**, with a transition enthalpy of 0.94 ± 0.07 kJ mol^–1^. Conversely, the DSC curve for **AH-V** exhibits a single thermal event, namely, melting at 187.3 ± 0.4 °C, with a Δ_fus_*H* of 24.43 ± 0.17 kJ mol^–1^.

Using the melting temperatures, heat of fusion values, and heat of transition values, we were able to construct a semischematic energy–temperature diagram for the SGD polymorphs (Figure 14b). The fact that the transition from **AH-II** to **AH-I** is endothermic suggests that these two polymorphs are enantiotropically^94^ related. The thermodynamic transition point from the measured melting points and heat of fusion values can be estimated using eq 1,^94−98^
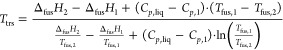
1where *T*_fus_ represents the melting point, Δ_fus_*H* is the heat of fusion, and *C*_*p*_ is the heat capacity. The subscripts 1 and 2 denote the higher and lower melting forms, respectively.

In cases where the heat capacities of the melt (*C*_*p*,liq_) and the higher melting form (*C*_*p*,1_) are not readily available, an empirical correction term, *k*Δ_fus_*H*_1_, can be applied to account for the heat capacity difference (*C*_*p*,liq_ – *C*_*p*,1_). Typical empirical values for *k* range from 0.001 to 0.007 K^–1^, with an average of *k* = 0.003 K^–1^.^96^

The heat of fusion for **AH-II** was calculated as the sum of Δ_fus_*H*_1_ and Δ_trs_*H*_II–I_, and the melting point of **AH-II** was derived to be 174–176 °C from HSM investigations, in agreement with literature data,^53^ resulting in a *T*_trs_ value of approximately 50 °C. Consequently, **AH-II** is the thermodynamic form at RT, and **AH-I** is the high-temperature form (with a very high kinetic stability at RT), in agreement with an earlier report.^53^ Slurry experiments conducted with non-solvate-forming solvents revealed that no transformation was observed within 2 weeks when pure **AH-I** or **AH-II** was employed. Nevertheless, a discernible, albeit slow, transition from **AH-I** to **AH-II** became evident after 2 weeks when a mixture of the two polymorphs was used as the starting point.

**AH-V** exhibits a lower melting point compared to **AH-I** and displays a lower Δ_fus_*H* value, indicating a monotropic relationship^94^ between **AH-I** and **AH-V**, i.e., no intersection of the *G* isobars prior to the melting temperature in Figure 14b is observed. In contrast, an enantiotropic relationship is established between **AH-V** and **AH-II**, with **AH-V** having a higher melting point but a lower Δ_fus_*H*. It can be assumed that **AH-III** maintains a monotropic relationship with all three other forms. This assumption is supported by the fact that **AH-III** melts at a significantly lower temperature than the other three forms, and the three other forms were identified as the three lowest energy structures in Figure 13a. The comparison between experimental and computed energy differences (Δ*H* = −Δ*E*_latt_) reveals a reasonably good agreement in the values [**II** → **I**: 0.94 (exp)/1.58 (calc) kJ mol^–1^; **V** → **I**: 3.04 (exp)/1.96 (calc) kJ mol^–1^] and the 0 K stability order [**II** (most stable) > **I** > **V**].

#### 3.5.4 Moisture-Dependent Stability of SGD Anhydrates

**Figure 15:**
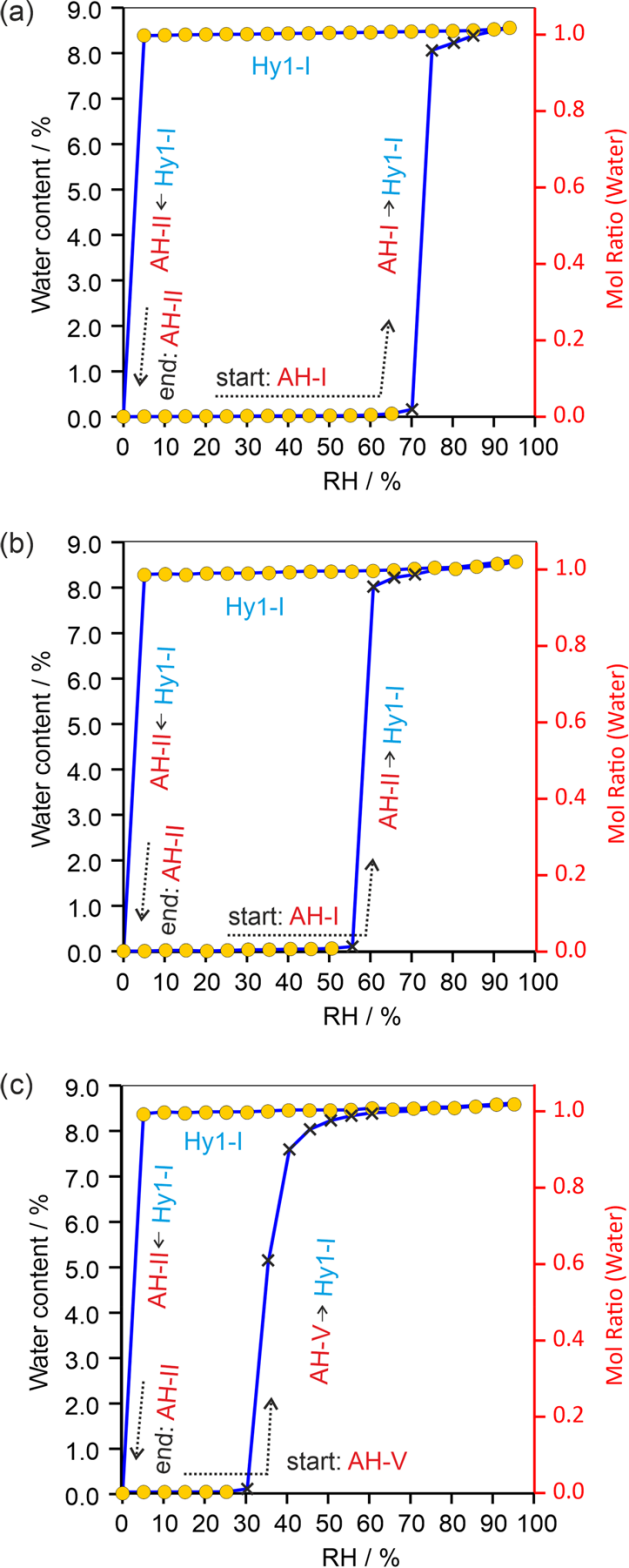
Gravimetric moisture (de)sorption curves of (a) **AH-I**, (b) **AH-II**, and (c) **AH-V**. The circles represent data points that are in equilibrium, whereas crosses mark data points where the sample did not reach the equilibrium moisture content.

Gravimetric studies on moisture-dependent (de)sorption were conducted, commencing at 0% RH, using the anhydrates **AH-I**, **AH-II**, and **AH-V** (Figure 15). All three anhydrates exhibit a single-step increase in mass as RH increases, corresponding to the transformation from anhydrate to **Hy1-I** (as confirmed by *ex situ* PXRD measurements). However, the three anhydrates differ significantly in the RH conditions at which the transformation to the hydrate occurs at RT. **AH-I** begins transforming into the hydrate at 70% RH, **AH-II** at 55%, and **AH-V** at a remarkably low 30%, making **AH-V** particularly challenging to handle. The only hydrate formed during water sorption is **Hy1-I**. Desorption studies confirmed that at RT, **Hy1-I** dehydrates into **AH-II**. Therefore, based on the sorption studies, the stability order of the anhydrates at RT is as follows: **AH-I** (most stable) > **AH-II** > **AH-V**. However, it is worth noting that **Hy1-I** should be considered the preferred form, as a transition into another solid form only occurs at the driest conditions (<5% RH) at RT.

Based on the structure comparisons, it was possible to elucidate the experimentally observed dehydration reaction from **Hy1-I** to **AH-II**. This is justified by the identical stacks of SG molecules present in both the hydrate and anhydrate structures. In the case of **AH-I** and **AH-V**, no structural relationship could be discerned. The strong hydration tendency of **AH-V** may be ascribed to its low density.

## 4 Conclusions

This comprehensive re-investigation of SGD has uncovered a wealth of previously unknown solid-state forms. Currently, a total of 16 solid-state forms of SGD are known. The finding of novel forms was largely facilitated by conducting slurry experiments in various organic solvents. The observation that the choice of the initial solid-state form played a pivotal role, leading to different outcomes in the formation of alcohol solvates (**S-MeOH** and **S-EtOH**), was of particular significance. This underscores the profound influence of transformation kinetics, solubility, and intermolecular interactions in solvent-mediated transformation reactions. The identification of the solvate forms was further complicated by the tendency of SGD to rapidly transform into one of its monohydrate forms (**Hy1-I**) under ambient conditions, potentially explaining why these solvates had remained elusive in previous studies. Nevertheless, the isolation of the solvate forms was paramount, as they can serve as precursors in the formation of the polymorphs **AH-I**, **AH-II**, and **AH-V**. A careful selection of the solvate (hydrate) form and desolvation temperature are essential factors that determine which anhydrate is obtained. In contrast, **AH-III**, a metastable polymorph, was observed solely during heating the SGD melt.

The computed anhydrate and monohydrate lattice energy landscapes have not only confirmed that the most stable polymorphs have been identified but also provided insights into their interaction and packing possibilities. In the hydrate structures, a strong chain motif involving water molecules was identified as the dominant monohydrate building block, consistent with the experimental hydrate structures. In contrast, the anhydrate crystal energy landscape exhibits a multitude of distinct H-bonding motifs and packings, rationalizing the high tendency toward polymorphism and the prevalence of structures with *Z*′ > 1. While alternative feasible structures have been predicted, their formation and isolation may be hindered by the exceptional stability of **Hy1-I**, which only undergoes dehydration at relative humidity levels <5% (at RT). The anhydrate crystal energy landscape also provides insights into the elusive **AH-IV** crystal structure.

The molecular features, including the H-bonding groups, along with the aromatic moiety, synergize with the broad energy wells of the rotatable dihedrals (which lack steric hindrance). This synergy facilitates the formation of the numerous strong intermolecular interactions, resulting in distinct densely packed (un)solvated structures. Within the crystal structures, unfavorable conformations (up to 20 kJ mol^–1^ less stable than the global minimum conformation) are compensated for by the molecule’s remarkable ability to form strong intermolecular interactions. This capability can be seen as the primary driving force for the stability of both anhydrous and solvated forms and prevalence for polymorphism.

To conclude, this study highlights the significance of gaining insights into the processes of polymorphic transformations, desolvation reactions, and stability across a range of temperature and humidity (solvent) conditions. This knowledge is crucial not only for the pharmaceutical and materials science fields but also for a deeper understanding of the fundamental principles governing solid-state chemistry and crystal engineering.

## Supporting Information Available

Virtual solid form screening: Crystal structure prediction studies; Experimental solid form screening: solvent evaporation, liquid-assisted grinding, slurry and solvent crystallization experiments; Crystallographic information; Pairwise intermolecular energy calculations; Characterization of the experimental forms: Powder X-ray diffraction, temperature-dependent IR spectroscopy, and compilation of physicochemical data (PDF)

## References

[ref1] Datta S.; Grant D. J. W. Crystal structures of drugs: Advances in determination, prediction and engineering. Nat. Rev. Drug Discovery 2004, 3 (1), 42–57. 10.1038/nrd1280. 14708020

[ref2] Aaltonen J.; Alleso M.; Mirza S.; Koradia V.; Gordon K. C.; Rantanen J. Solid form screening - A review. Eur. J. Pharm. Biopharm 2009, 71 (1), 23–37. 10.1016/j.ejpb.2008.07.014. 18715549

[ref3] Singhal D.; Curatolo W. Drug polymorphism and dosage form design: a practical perspective. Adv. Drug Delivery Rev. 2004, 56 (3), 335–347. 10.1016/j.addr.2003.10.008. 14962585

[ref4] Hilfiker R.; Blatter F.; von Raumer M. Relevance of solid-state properties for pharmaceutical products. In Polymorphism; Hilfiker R., Ed.; Wiley: 2006; pp 1–19.

[ref5] Censi R.; Di Martino P. Polymorph Impact on the Bioavailability and Stability of Poorly Soluble Drugs. Molecules 2015, 20 (10), 18759–76. 10.3390/molecules201018759. 26501244 PMC6331817

[ref6] Brittain H. G. Polymorphism in Pharmaceutical Solids; Informa Healthcare: New York, London, 2009; Vol. 192.

[ref7] Byrn S. R.; Pfeiffer R. R.; Stowell J. G. Solid-State Chemistry of Drugs. 2nd ed.; SSCI, Inc.: West Lafayette, IN, 1999.

[ref8] Reutzel-Edens S. M.; Braun D. E.; Newman A. W. Hygroscopicity and Hydrates in Pharmaceutical Solids. In Polymorphism in the Pharmaceutical Industry: Solid Form and Drug Development; Hilfiker R., Von Raumer M., Eds.; Wiley-VCH: 2019; Vol. 2.

[ref9] Griesser U. J., The importance of solvates. In Polymorphism: In the Pharmaceutical Industry, Hilfiker R., Ed.; Wiley-VCH: Germany, 2006; pp 211–233.

[ref10] Healy A. M.; Worku Z. A.; Kumar D.; Madi A. M. Pharmaceutical solvates, hydrates and amorphous forms: A special emphasis on cocrystals. Adv. Drug Delivery Rev. 2017, 117, 25–46. 10.1016/j.addr.2017.03.002. 28342786

[ref11] Yu K.; Shen J.; Liu J.; Tang G.; Hu X. Stability and Mechanical Properties of Darunavir Isostructural Solvates: An Experimental and Computational Study. Cryst. Growth Des. 2023, 23 (4), 2905–2915. 10.1021/acs.cgd.3c00057.

[ref12] Braun D. E.; Lingireddy S. R.; Beidelschies M. D.; Guo R.; Mueller P.; Price S. L.; Reutzel-Edens S. M. Unraveling Complexity in the Solid Form Screening of a Pharmaceutical Salt: Why so Many Forms? Why so Few?. Cryst. Growth Des. 2017, 17 (10), 5349–5365. 10.1021/acs.cgd.7b00842. PMC5629560 29018305

[ref13] Braun D. E.; Kahlenberg V.; Gelbrich T.; Ludescher J.; Griesser U. J. Solid state characterisation of four solvates of R-cinacalcet hydrochloride. CrystEngComm 2008, 10 (11), 1617–1625. 10.1039/b809219b.

[ref14] Chemburkar S. R.; Bauer J.; Deming K.; Spiwek H.; Patel K.; Morris J.; Henry R.; Spanton S.; Dziki W.; Porter W.; Quick J.; Bauer P.; Donaubauer J.; Narayanan B. A.; Soldani M.; Riley D.; McFarland K. Dealing with the Impact of Ritonavir Polymorphs on the Late Stages of Bulk Drug Process Development. Org. Process Res. Dev. 2000, 4 (5), 413–417. 10.1021/op000023y.

[ref15] Perez-Lloret S.; Rey M. V.; Ratti P. L.; Rascol O. Rotigotine transdermal patch for the treatment of Parkinson’s Disease. Fundam. Clin. Pharmacol. 2013, 27 (1), 81–95. 10.1111/j.1472-8206.2012.01028.x. 22320451

[ref16] Bučar D.-K.; Lancaster R. W.; Bernstein J. Disappearing Polymorphs Revisited. Angew Chem Int Ed 2015, 54 (24), 6972–6993. 10.1002/anie.201410356. PMC4479028 26031248

[ref17] Cuppen H. M.; Smets M. M. H.; Krieger A. M.; van den Ende J. A.; Meekes H.; van Eck E. R. H.; Görbitz C. H. The Rich Solid-State Phase Behavior of l-Phenylalanine: Disappearing Polymorphs and High Temperature Forms. Cryst. Growth Des. 2019, 19 (3), 1709–1719. 10.1021/acs.cgd.8b01655. PMC6410616 30872978

[ref18] Bhardwaj R. M.; McMahon J. A.; Nyman J.; Price L. S.; Konar S.; Oswald I. D. H.; Pulham C. R.; Price S. L.; Reutzel-Edens S. M. A Prolific Solvate Former, Galunisertib, under the Pressure of Crystal Structure Prediction, Produces Ten Diverse Polymorphs. J. Am. Chem. Soc. 2019, 141 (35), 13887–13897. 10.1021/jacs.9b06634. 31394896

[ref19] Du W.; Wang H.; Wang R.; Wang X.; Cheng P.; Zhang J.; Tang N.; Zhu L.; Cui P. Conformational Flexibility and Crystallization: The Case of 4-Hexyloxybenzoic Acid. Cryst. Growth Des. 2020, 20 (12), 7694–7703. 10.1021/acs.cgd.0c00960.

[ref20] Nowak M.; Dyba A. J.; Janczak J.; Morritt A.; Fabian L.; Karolewicz B.; Khimyak Y. Z.; Braun D. E.; Nartowski K. P. Directing Crystallization Outcomes of Conformationally Flexible Molecules: Polymorphs, Solvates, and Desolvation Pathways of Fluconazole. Mol. Pharmaceutics 2022, 19 (2), 456–471. 10.1021/acs.molpharmaceut.1c00752. 35050637

[ref21] Braun D. E.; Gelbrich T.; Kahlenberg V.; Laus G.; Wieser J.; Griesser U. J. Packing Polymorphism of a Conformationally Flexible Molecule (Aprepitant). New J. Chem. 2008, 32, 1677–1685. 10.1039/b805438j.

[ref22] Surov A. O.; Manin A. N.; Voronin A. P.; Churakov A. V.; Perlovich G. L.; Vener M. V. Weak Interactions Cause Packing Polymorphism in Pharmaceutical Two-Component Crystals. The Case Study of the Salicylamide Cocrystal. Cryst. Growth Des. 2017, 17 (3), 1425–1437. 10.1021/acs.cgd.7b00019.

[ref23] Bhowal R.; Chopra D. Investigating the Role of Weak Interactions to Explore the Polymorphic Diversity in Difluorinated Isomeric N-Phenylcinnamamides. Cryst. Growth Des. 2021, 21 (7), 4162–4177. 10.1021/acs.cgd.1c00422.

[ref24] Kuhnert-Brandstaetter M. A. Pharm. Unserer Zeit 1975, 4, 131–137. 10.1002/pauz.19750040501. 1181594

[ref25] Braun D. E.; Vickers M.; Griesser U. J. Dapsone Form V: A Late Appearing Thermodynamic Polymorph of a Pharmaceutical. Mol. Pharmaceutics 2019, 16, 3221–3236. 10.1021/acs.molpharmaceut.9b00419. 31075201

[ref26] Braun D. E.; Gelbrich T.; Wurst K.; Griesser U. J. Computational and Experimental Characterization of Five Crystal Forms of Thymine: Packing Polymorphism, Polytypism/Disorder and Stoichiometric 0.8-Hydrate. Cryst. Growth Des 2016, 16 (6), 3480–3496. 10.1021/acs.cgd.6b00459. PMC5486440 28663717

[ref27] Gataullina K. V.; Buzyurov A. V.; Gerasimov A. V.; Gatiatulin A. K.; Ziganshin M. A.; Schick C.; Gorbatchuk V. V. New Polymorph of Indomethacin: Screening by Solid-State Guest Exchange and Characterization Using Fast Scanning Calorimetry. Cryst. Growth Des. 2023, 23, 7109 10.1021/acs.cgd.3c00443.

[ref28] Li S.; Liu B.; Chen Z.; Ou X.; Rong H.; Lu M. Ritonavir Revisited: Melt Crystallization Can Easily Find the Late-Appearing Polymorph II and Unexpectedly Discover a New Polymorph III. Mol. Pharmaceutics 2023, 20 (8), 3854–3863. 10.1021/acs.molpharmaceut.2c00994. 37450774

[ref29] Gift A. D.; Luner P. E.; Luedeman L.; Taylor L. S. Manipulating Hydrate Formation During High Shear Wet Granulation Using Polymeric Excipients. J. Pharm. Sci. 2009, 98 (12), 4670–4683. 10.1002/jps.21763. 19455624

[ref30] Bhattacharya S.; Saha B. K. Polymorphism through Desolvation of the Solvates of a van der Waals Host. Cryst. Growth Des. 2013, 13 (2), 606–613. 10.1021/cg301269d.

[ref31] Braun D. E. Experimental and computational approaches to rationalise multicomponent supramolecular assemblies: dapsone monosolvates. Phys. Chem. Chem. Phys. 2019, 21 (21), 17288–17305. 10.1039/C9CP02572C. 31348477

[ref32] Li Z.; Ouyang R.; Shi P.; Du S.; Gong J.; Wu S. Rationalizing the Formation of Belinostat Solvates with Experimental Screening and Computational Predictions. Cryst. Growth Des. 2021, 21 (9), 4986–4996. 10.1021/acs.cgd.1c00435.

[ref33] Xiong X.; Du Q.; Zeng X.; He J.; Yang H.; Li H. Solvates and polymorphs of rebamipide: preparation, characterization, and physicochemical analysis. RSC Adv. 2017, 7 (38), 23279–23286. 10.1039/C7RA02895D.

[ref34] Abramov Y. A.; Loschen C.; Klamt A. Rational Coformer or Solvent Selection for Pharmaceutical Cocrystallization or Desolvation. J. Pharm. Sci. 2012, 101 (10), 3687–3697. 10.1002/jps.23227. 22821740

[ref35] Cruz-Cabeza A. J.; Wright S. E.; Bacchi A. On the entropy cost of making solvates. Chem. Commun. (Cambridge, U. K.) 2020, 56 (38), 5127–5130. 10.1039/D0CC01050B. 32267257

[ref36] Takieddin K.; Khimyak Y. Z.; Fabian L. Prediction of Hydrate and Solvate Formation Using Statistical Models. Cryst. Growth Des. 2016, 16 (1), 70–81. 10.1021/acs.cgd.5b00966.

[ref37] Johnston A.; Johnston B. F.; Kennedy A. R.; Florence A. J. Targeted crystallisation of novel carbamazepine solvates based on a retrospective Random Forest classification. CrystEngComm 2008, 10, 23–25. 10.1039/B713373A.

[ref38] Loschen C.; Klamt A. Solubility prediction, solvate and cocrystal screening as tools for rational crystal engineering. J. Pharm. Pharmacol. 2015, 67 (6), 803–811. 10.1111/jphp.12376. 25851032

[ref39] Infantes L.; Fabian L.; Motherwell W. D. S. Organic crystal hydrates: what are the important factors for formation. CrystEngComm 2007, 9 (1), 65–71. 10.1039/B612529H.

[ref40] Nangia A.; Desiraju G. R. Pseudopolymorphism: occurrences of hydrogen bonding organic solvents in molecular crystals. Chem. Commun. 1999, (7), 605–606. 10.1039/a809755k.

[ref41] van de Streek J.; Motherwell S. New software for searching the Cambridge Structural Database for solvated and unsolvated crystal structures applied to hydrates. CrystEngComm 2007, 9 (1), 55–64. 10.1039/B613332K.

[ref42] Brychczynska M.; Davey R. J.; Pidcock E. A study of methanol solvates using the Cambridge structural database. New J. Chem. 2008, 32 (10), 1754–1760. 10.1039/b810063m.

[ref43] Brychczynska M.; Davey R. J.; Pidcock E. A study of dimethylsulfoxide solvates using the Cambridge Structural Database (CSD). CrystEngComm 2012, 14 (4), 1479–1484. 10.1039/C1CE05464C.

[ref44] Boothroyd S.; Kerridge A.; Broo A.; Buttar D.; Anwar J. Why Do Some Molecules Form Hydrates or Solvates?. Cryst. Growth Des. 2018, 18 (3), 1903–1908. 10.1021/acs.cgd.8b00160.

[ref45] Sköld O. Sulfonamide resistance: mechanisms and trends. Drug Resistance Updates 2000, 3 (3), 155–160. 10.1054/drup.2000.0146. 11498380

[ref46] Skold O. Resistance to trimethoprim and sulfonamides. Vet. Res. 2001, 32, 261–273. 10.1051/vetres:2001123. 11432417

[ref47] Abidi S. S. A.; Azim Y.; Khan S. N.; Khan A. U. Sulfaguanidine cocrystals: Synthesis, structural characterization and their antibacterial and hemolytic analysis. J. Pharm. Biomed. Anal. 2018, 149, 351–357. 10.1016/j.jpba.2017.11.028. 29145096

[ref48] Gibreel A.; Sköld O. Sulfonamide Resistance in Clinical Isolates of *Campylobacter jejuni*: Mutational Changes in the Chromosomal Dihydropteroate Synthase. Antimicrob Agents Chemother 1999, 43 (9), 2156–2160. 10.1128/AAC.43.9.2156. 10471557 PMC89439

[ref49] De Liguoro M.; Riga A.; Fariselli P. Synergistic toxicity of some sulfonamide mixtures on Daphnia magna. Ecotoxicology and Environmental Safety 2018, 164, 84–91. 10.1016/j.ecoenv.2018.08.011. 30098509

[ref50] Dalla Bona M.; Di Leva V.; De Liguoro M. The sensitivity of Daphnia magna and Daphnia curvirostris to 10 veterinary antibacterials and to some of their binary mixtures. Chemosphere 2014, 115, 67–74. 10.1016/j.chemosphere.2014.02.003. 24630458

[ref51] Kuhnert-Brandstätter M. Polymorphie bei Arzneistoffen. Oesterr. Apoth. Ztg. 1959, 13, 297.

[ref52] Kuhnert-Brandstaetter M.; Wunsch S. Polymorphism and solid solution formation in sulfonamides and related compounds. I. Mikrochim. Acta 1969, 57 (6), 1297–1307. 10.1007/BF01271407. 5365039

[ref53] Kuhnert-Brandstätter M.; Bachleitebr-Hofmann F. I.R.-spektroskopische untersuchungen an enantiotropen kristall-modiflkationen von sulfonamiden. Spectrochimica Acta Part A: Molecular Spectroscopy 1971, 27 (2), 191–198. 10.1016/0584-8539(71)80024-7.

[ref54] Alberola S.; Rambaud J.; Sabon F. Study of the polymorphism of sulfaguanidine. Bulletin de la Societe Chimique de France 1977, (3–4), 181–4.

[ref55] Mesley R. J.; Houghton E. E. Infrared identification of pharmaceutically important sulfonamides with particular reference to the occurrence of polymorphism. J. Pharm. Pharmacol. 2011, 19 (5), 295–304. 10.1111/j.2042-7158.1967.tb08090.x. 4382401

[ref56] Yang S. S.; Guillory J. K. Polymorphism in sulfonamides. J. Pharm. Sci. 1972, 61 (1), 26–40. 10.1002/jps.2600610104. 5066733

[ref57] Eccles K. S.; Stokes S. P.; Daly C. A.; Barry N. M.; McSweeney S. P.; O’Neill D. J.; Kelly D. M.; Jennings W. B.; Ni Dhubhghaill O. M.; Moynihan H. A.; Maguire A. R.; Lawrence S. E. Evaluation of the Bruker SMART X2S: crystallography for the nonspecialist?. J. Appl. Crystallogr. 2011, 44 (1), 213–215. 10.1107/S0021889810042561. 22477782 PMC3253739

[ref58] Koo C. H.; Kim H. S.; Shin W.; Choe C. J. Korean Chem. Soc. 1974, 18, 97.

[ref59] Alberola S.; Rambaud J.; Sabon F. Study of the antipyrine-sulfaguanidine system by x-ray diffraction and differential enthalpic analysis. Annales Pharmaceutiques Francaises 1976, 34 (3–4), 95–9. 984686

[ref60] Huang S.; Cheemarla V. K. R.; Tiana D.; Lawrence S. E. Experimental and Theoretical Investigation of Hydrogen-Bonding Interactions in Cocrystals of Sulfaguanidine. Cryst. Growth Des. 2023, 23 (4), 2306–2320. 10.1021/acs.cgd.2c01337. PMC10080660 37038403

[ref61] Frisch M. J.; Trucks G. W.; Schlegel H. B.; Scuseria G. E.; Robb J. M. A.; Cheeseman R.; Scalmani G.; Barone V.; Mennucci B.; Petersson G. A.; Nakatsuji H.; Caricato M.; Li X.; Hratchian H. P.; Izmaylov A. F.; Bloino J.; Zheng G.; Sonnenberg J. L.; Hada M.; Ehara M.; Toyota K.; Fukuda R.; Hasegawa J.; Ishida M.; Nakajima T.; Honda Y.; Kitao O.; Nakai H.; Vreven T.; Montgomery J. A.; Peralta J. E.; Ogliaro F.; Bearpark M.; Heyd J. J.; Brothers E.; Kudin K. N.; Staroverov V. N.; Kobayashi R.; Normand J.; Raghavachari K.; Rendell A.; Burant J. C.; Iyengar S. S.; Tomasi J.; Cossi M.; Rega N.; Millam J. M.; Klene M.; Knox J. E.; Cross J. B.; Bakken V.; Adamo C.; Jaramillo J.; Gomperts R.; Stratmann R. E.; Yazyev O.; Austin A. J.; Cammi R.; Pomelli C.; Ochterski J. W.; Martin R. L.; Morokuma K.; Zakrzewski V. G.; Voth G. A.; Salvador P.; Dannenberg J. J.; Dapprich S.; Daniels A. D.; Farkas O.; Foresman J. B.; Ortiz J. V.; Cioslowski J.; Fox D. J. Gaussian 09; Gaussian Inc.: Wallingford CT, 2009.

[ref62] Karamertzanis P. G.; Pantelides C. C. Ab initio crystal structure prediction-I. Rigid molecules. J. Comput. Chem. 2005, 26 (3), 304–324. 10.1002/jcc.20165. 15622548

[ref63] Karamertzanis P. G.; Pantelides C. C. Ab initio crystal structure prediction. II. Flexible molecules. Mol. Phys. 2007, 105 (2–3), 273–291. 10.1080/00268970601143317. 15622548

[ref64] Habgood M.; Sugden I. J.; Kazantsev A. V.; Adjiman C. S.; Pantelides C. C. Efficient Handling of Molecular Flexibility in Ab Initio Generation of Crystal Structures. J. Chem. Theory Comput 2015, 11 (4), 1957–1969. 10.1021/ct500621v. 26574397

[ref65] Price S. L.; Leslie M.; Welch G. W. A.; Habgood M.; Price L. S.; Karamertzanis P. G.; Day G. M. Modelling organic crystal structures using distributed multipole and polarizability-based model intermolecular potentials. Phys. Chem. Chem. Phys. 2010, 12 (30), 8478–8490. 10.1039/c004164e. 20607186

[ref66] Kazantsev A. V.; Karamertzanis P. G.; Adjiman C. S.; Pantelides C. C. Efficient Handling of Molecular Flexibility in Lattice Energy Minimization of Organic Crystals. J. Chem. Theory Comput 2011, 7 (6), 1998–2016. 10.1021/ct100597e. 26596459

[ref67] Stone A. J. Distributed multipole analysis: Stability for large basis sets. J. Chem. Theory Comput. 2005, 1 (6), 1128–1132. 10.1021/ct050190+. 26631656

[ref68] Coombes D. S.; Price S. L.; Willock D. J.; Leslie M. Role of Electrostatic Interactions in Determining the Crystal Structures of Polar Organic Molecules. A Distributed Multipole Study. J. Phys. Chem. 1996, 100 (18), 7352–7360. 10.1021/jp960333b.

[ref69] Clark S. J.; Segall M. D.; Pickard C. J.; Hasnip P. J.; Probert M. J.; Refson K.; Payne M. C. First principles methods using CASTEP. Zeitschrift fur Kristallographie 2005, 220 (5–6), 567–570. 10.1524/zkri.220.5.567.65075.

[ref70] Perdew J. P.; Burke K.; Ernzerhof M. Generalized gradient approximation made simple. Phys. Rev. Lett. 1996, 77 (18), 3865–3868. 10.1103/PhysRevLett.77.3865. 10062328

[ref71] Vanderbilt D. Soft Self-Consistent Pseudopotentials in a Generalized Eigenvalue Formalism. Phys. Rev. B 1990, 41 (11), 7892–7895. 10.1103/PhysRevB.41.7892. 9993096

[ref72] Tkatchenko A.; Alfe D.; Kim K. S. First-Principles Modeling of Non-Covalent Interactions in Supramolecular Systems: The Role of Many-Body Effects. J. Chem. Theory Comput. 2012, 8 (11), 4317–4322. 10.1021/ct300711r. 26605594

[ref73] Macrae C. F.; Sovago I.; Cottrell S. J.; Galek P. T. A.; McCabe P.; Pidcock E.; Platings M.; Shields G. P.; Stevens J. S.; Towler M.; Wood P. A. Mercury 4.0: from visualization to analysis, design and prediction. J. Appl. Crystallogr. 2020, 53 (1), 226–235. 10.1107/S1600576719014092. 32047413 PMC6998782

[ref74] Turner M. J.; Grabowsky S.; Jayatilaka D.; Spackman M. A. Accurate and Efficient Model Energies for Exploring Intermolecular Interactions in Molecular Crystals. J. Phys. Chem. Lett. 2014, 5 (24), 4249–4255. 10.1021/jz502271c. 26273970

[ref75] Turner M. J.; Thomas S. P.; Shi M. W.; Jayatilaka D.; Spackman M. A. Energy frameworks: insights into interaction anisotropy and the mechanical properties of molecular crystals. Chem. Commun. 2015, 51 (18), 3735–3738. 10.1039/C4CC09074H. 25525647

[ref76] Mackenzie C. F.; Spackman P. R.; Jayatilaka D.; Spackman M. A. CrystalExplorer model energies and energy frameworks: extension to metal coordination compounds, organic salts, solvates and open-shell systems. IUCrJ. 2017, 4 (5), 575 10.1107/S205225251700848X. 28932404 PMC5600021

[ref77] Turner M.; McKinnon J.; Wolff S.; Grimwood D.; Spackman P.; Jayatilaka D.; Spackman M. CrystalExplorer17; The University of Western Australia: 2017.

[ref78] Tkatchenko A.; DiStasio R. A. J.; Car R.; Scheffler M. Accurate and efficient method for many-body van der Waals interactions. Phys. Rev. Lett. 2012, 108 (23), 236402–236402. 10.1103/PhysRevLett.108.236402. 23003978

[ref79] Hoja J.; Ko H.-Y.; Neumann M. A.; Car R.; DiStasio R. A. Jr; Tkatchenko A. Reliable and practical computational prediction of molecular crystal polymorphs. arXiv.org, e-Print Arch., Phys. 2018, 1803.07503 10.48550/arXiv.1803.07503. PMC6357866 30746448

[ref80] Frisch M. J.; Trucks G. W.; Schlegel H. B.; Scuseria G. E.; Robb M. A.; Cheeseman J. R.; Scalmani G.; Barone V.; Petersson G. A.; Nakatsuji H.; Li X.; Caricato M.; Marenich A. V.; Bloino J.; Janesko B. G.; Gomperts R.; Mennucci B.; Hratchian H. P.; Ortiz J. V.; Izmaylov A. F.; Sonnenberg J. L.; Williams; Ding F.; Lipparini F.; Egidi F.; Goings J.; Peng B.; Petrone A.; Henderson T.; Ranasinghe D.; Zakrzewski V. G.; Gao J.; Rega N.; Zheng G.; Liang W.; Hada M.; Ehara M.; Toyota K.; Fukuda R.; Hasegawa J.; Ishida M.; Nakajima T.; Honda Y.; Kitao O.; Nakai H.; Vreven T.; Throssell K.; Montgomery J. A. Jr.; Peralta J. E.; Ogliaro F.; Bearpark M. J.; Heyd J. J.; Brothers E. N.; Kudin K. N.; Staroverov V. N.; Keith T. A.; Kobayashi R.; Normand J.; Raghavachari K.; Rendell A. P.; Burant J. C.; Iyengar S. S.; Tomasi J.; Cossi M.; Millam J. M.; Klene M.; Adamo C.; Cammi R.; Ochterski J. W.; Martin R. L.; Morokuma K.; Farkas O.; Foresman J. B.; Fox D. J. Gaussian 16, Rev. C.01, Wallingford, CT, 2016.

[ref81] Markvardsen A. J.; David W. I. F.; Johnson J. C.; Shankland K. A probabilistic approach to space-group determination from powder diffraction data. Acta Crystallogr., Sect. A: Found. Crystallogr. 2001, A57 (1), 47–54. 10.1107/S0108767300012174. 11124502

[ref82] David W. I. F.; Shankland K.; van de Streek J.; Pidcock E.; Motherwell W. D. S.; Cole J. C. DASH: a program for crystal structure determination from powder diffraction data. J. Appl. Crystallogr. 2006, 39, 910–915. 10.1107/S0021889806042117.

[ref83] Pawley G. S. Unit-Cell Refinement from Powder Diffraction Scans. J. Appl. Crystallogr. 1981, 14 (DEC), 357–361. 10.1107/S0021889881009618.

[ref84] Rietveld H. M. A Profile Refinement Method for Nuclear and Magnetic Structures. J. Appl. Crystallogr. 1969, 2, 65–71. 10.1107/S0021889869006558.

[ref85] Coelho A. A. Topas Academic; Coelho Software: Brisbane, 2020.

[ref86] Kuhnert-Brandstaetter M.; Proell F. Thermal analysis of hydrates of organic compounds. II. Mikrochim. Acta 1983, 81, 287–300. 10.1007/BF01196713.

[ref87] Taylor R.; Wood P. A. A Million Crystal Structures: The Whole Is Greater than the Sum of Its Parts. Chem. Rev. (Washington, DC, U. S.) 2019, 119 (16), 9427–9477. 10.1021/acs.chemrev.9b00155. 31244003

[ref88] Alleaume M.; Gulko A.; Herbstein F. H.; Kapon M.; Marsh R. E. Comparison of the dimensions and conformation of the sulfaguanidine moiety in sulfaguanidine monohydrate and trans-dichlorobis(sulfaguanidine)palladium(II). Acta Crystallographica Section B 1976, 32 (3), 669–682. 10.1107/S0567740876003774.

[ref89] Etter M. C.; MacDonald J. C.; Bernstein J. Graph-set analysis of hydrogen-bond patterns in organic crystals. Acta Crystallographica, Section B: Structural Science 1990, B46 (2), 256–262. 10.1107/S0108768189012929. 2344397

[ref90] Price S. L.; Braun D. E.; Reutzel-Edens S. M. Can computed crystal energy landscapes help understand pharmaceutical solids?. Chem. Commun. 2016, 52, 7065–7077. 10.1039/C6CC00721J. PMC5486446 27067116

[ref91] Bingham A. L.; Hughes D. S.; Hursthouse M. B.; Lancaster R. W.; Tavener S.; Threlfall T. L. Over one hundred solvates of sulfathiazole. Chem. Commun. (Cambridge, U. K.) 2001, (7), 603–604. 10.1039/b009540k.

[ref92] Kalman A.; Czugler M.; Argay G. Conformational characteristics of anhydrous sulfaguanidine: computer retrieval and analysis of N-substituted arylsulfonamides. Acta Crystallographica Section B 1981, 37 (4), 868–877. 10.1107/S0567740881004494.

[ref93] Rohlicek J.; Skorepova E.; Babor M.; Cejka J. CrystalCMP: an easy-to-use tool for fast comparison of molecular packing. J. Appl. Crystallogr. 2016, 49 (6), 2172–2183. 10.1107/S1600576716016058.

[ref94] Burger A.; Ramberger R. On the polymorphism of pharmaceuticals and other molecular crystals. I. Theory of thermodynamic rules. Mikrochim. Acta 1979, 72, 259–271. 10.1007/BF01197379.

[ref95] Burger A.; Ramberger R. On the polymorphism of pharmaceuticals and other molecular crystals. II. Applicability of thermodynamic rules. Mikrochim. Acta 1979, 72, 273–316. 10.1007/BF01197380.

[ref96] Yu L. Inferring Thermodynamic Stability Relationship of Polymorphs from Melting Data. J. Pharm. Sci. 1995, 84 (8), 966–974. 10.1002/jps.2600840812. 7500282

[ref97] Griesser U. J.; Weigand D.; Rollinger J. M.; Haddow M.; Gstrein E. The Crystal Polymorphs of Metazachlor: Identification and thermodynamic stability. J. Therm. Anal. Calorim. 2004, 77, 511–522. 10.1023/B:JTAN.0000038990.43475.db.

[ref98] Braun D. E.; Gelbrich T.; Kahlenberg V.; Tessadri R.; Wieser J.; Griesser U. J. Conformational Polymorphism in Aripiprazole: Preparation, Stability and Structure of Five Modifications. J. Phram. Sci. 2009, 98 (6), 2010–2026. 10.1002/jps.21574. 19009597

