# Supplementary material for: Comprehensive Insights into Sulfaguanidine in the Solid State: An Experimental and Computational Study
Source: Cryst Growth Des. 2024 Jan 17;24(3):1438–57. doi: 10.1021/acs.cgd.3c01384 (PMC10853908; doi:10.1021/acs.cgd.3c01384)
Supplement: Supplementary file 1 — cg3c01384_si_001.pdf [file cg3c01384_si_001.pdf]

Supplementary Information

Comprehensive Insights into Sulfaguanidine in the Solid State: An Experimental and Computational Study

Alexandre Widauer, Tom L. Petrick and Doris E. Braun*

Institute of Pharmacy, University of Innsbruck, Innrain 52c, 6020 Innsbruck, Austria

*Email: doris.braun@uibk.ac.at

Table of Contents

1. Literature forms 3

2. Virtual solid form screening 5

2.1. Input conformations for computational studies 5

2.2. Computational generation of the SGD anhydrate crystal energy landscape 6

2.3. Representation of the experimental SGD anhydrate structures 8

2.4. CSP: AH-II cluster and potential AH-IV structures 9

2.5. Computational generation of the SGD monohydrate crystal energy landscape 14

2.6. Representation of the experimental monohydrate structures 15

2.7. Multi-component hydrogen-bond propensity screen for SGD solvates 16

3. Experimental solid form screening 18

3.1. Solvent evaporation experiments 18

3.2. Liquid-assisted grinding experiments 18

3.3. Slurry experiments 19

3.4. Solvent crystallization experiments 21

4. Crystal structures 24

4.1. Structure solution from PXRD data 24

4.2. Pairwise intermolecular interaction-energy calculations 31

4.3. Experimental Sulfaguanidine conformations 39

5. Characterization of the solid-state forms 40

5.1. Powder X-ray diffraction 40

5.2. Temperature dependent IR spectroscopy 41

5.3. Physicochemical data for SGD solid-state forms 44

References 45

1. Literature forms

Several studies already reported polymorphism and hydrate formation of SGD. Table S1 summarizes the literature data and unites the different designations of the solid-state forms.

Table S1. Overview SGD literate forms.

Publication	amor-phous	AH-I	AH-II	AH-III	AH-IV	AH-V	Hy1-I	Hy1-II	Solvates	Other forms or mixtures
Kuhnert-Brandstätter 1959[1]		**I**	**II**							
Mesley 1967[2]	amor-phous	**B**					**A**			**C, D**
Kuhnert-Brandstätter 1969[3]		**I**	**II**	**III**			**Hydrate**			
Kuhnert-Brandstätter 1971[4]		**I**	**II**	III			**Hydrate**			
Yang 1972[5]		**III**					**SI**			**II, IV ~ V[a]**
Alberola1977[6]		**I**	**II**		**III**		**Hydrate**		**S_{ACO}**	
Eccles2011[7]								**Hydrate**		
This work		**I**	**II**	**III**	**IV**	**V**	**Hy1-I**	**Hy1-II**	**S_{ACO} and others**	

[a] identical IR spectra, according to the authors.

Kuhnert-Brandstätter and coauthors reported three different polymorphs (Mod. **I**, **II**, and **III**) and a **monohydrate**. Melting temperatures have been provided, allowing the identification of the anhydrate polymorphs[3] and the hydrate[4]. Furthermore, the stability was assessed and Mod. **II** identified as the most stable polymorph at room temperature.

Yang and Guillory reported data for five different solid-state forms of SGD, with SI corresponding to the monohydrate. Figure S1 shows a plot of the *d*-spacing values reported[5] by the authors. Regions characteristic for **AH-I**, **AH-II**, **AH-V** and **Hy1-I** are marked. The Yang SI and III solid-state forms can be assigned to **Hy1-I** and **AH-I**, respectively. Forms II and V shows peak positions which can be assigned to **Hy1-I** and different anhydrates. In case of III the majority of the peak positions can be assigned to the structurally characterized forms.

Mesley and Houghton reported on the existence of five SGD solid-state forms, amorphous SGD, one hydrate and three anhydrate polymorphs.[2] Form A corresponds to **Hy1-I**. Considering the solvents used in this study, namely MeOH, EtOH, and acetone, which were found in our study to induce the formation of solvates, and depending on the desolvation and storage conditions, different crystalline forms may be expected. Therefore, it is plausible that the literature sample consisted of either **AH-I**, **AH-II, AH-V**, **Hy1-I**, or phase mixtures.

Alberola and coauthors provided analytical data (thermal analysis and lattice parameters) for three anhydrates I, II, and III, as well as for a hydrate and an acetone solvate (S_{ACO}).[6] Anhydrates I and II correspond to **AH-I** and **AH-II**, respectively. Anhydrate III, which shows a 4.24% increase in volume compared to **AH-II**, does not correspond to **AH-III** and is therefore renamed to **AH-IV**.

Eccles and coauthors reported the structure of a second SGD monohydrate[7] (**Hy1-II**).

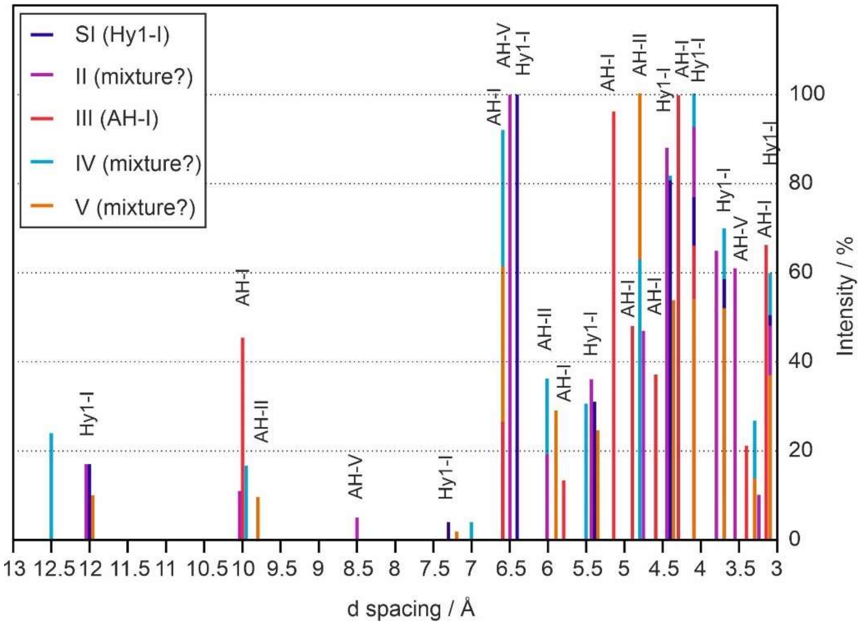

Figure S1. *d*-Spacing values for the SGD crystal forms reported by Yang and Guillory.[5] Characteristic peak positions for **AH-I**, **AH-II**, **AH-V** and **Hy1-I** have been marked.

An overview of all crystal structure/lattice parameter determinations is given in Table S2.

Table S2. Overview of SGD crystal structures/lattice parameters.

CSD Ref. code	Solid form	a / Å	b / Å	c / Å	α / °	β / °	γ / °	Vol / Å³	Space group
					Anhydrates				
ZZZAYP[6]	**AH-I**	10.414	20.219	7.037	82.86	95.29	95.93	1457.62	?
ZZZAYP01[6]	**AH-II**	9.500	7.475	24.732	90	96.10	90	1746.34	$P2_1/c$
ZZZAYP02[6]	**AH-IV**	10.032	7.747	24.662	90	94.85	90	1909.82	$P2_1/c$
ZZZAYP03[8]	**AH-II**	9.912	7.530	24.496	90	95.32	90	1820.44	$P2_1/c$
This work	**AH-I**	6.992	10.159	20.589	99.78	94.84	97.58	1420.06	P-1
This work	**AH-V**	10.056	10.1478	10.958	90	108.49	90	1060.46	$P2_1/n$
					Hydrates				
SOGUAN01[9]	**Hy1-I**	7.57	5.44	24.76	90	91.0	90	1019.48	$P2_1/c$
SOGUAN02[6]	**Hy1-I**	7.536	5.450	24.593	90	90.34	90	1010.05	$P2_1/c$
SOGUAN03[7]	**Hy1-II**	5.610	7.330	12.491	90	93.30	90	512.74	$P2_1$
SOGUAN04[7]	**Hy1-II**	5.610	7.330	12.491	90	93.30	90	512.74	$P2_1$
SOGUAN05[7]	**Hy1-II**	5.625	7.354	12.521	90	93.45	90	516.97	$P2_1$
SOGUAN06[7]	**Hy1-II**	5.625	7.354	12.521	90	93.45	90	516.97	$P2_1$
SOGUAN20[10]	**Hy1-I**	7.515	5.554	24.592	90	90.47	90	1026.39	$P2_1/c$
					Solvates				
ZZZAYM[6]	$\mathbf{S_{ACO}}$	9.548	19.981	12.947	90	90	90	2470.01	*Pbcm*
This work	$\mathbf{S_{ACO}}$	12.661	18.741	19.278	90	90	90	4574.46	*Pbca*
This work	$\mathbf{S_{DMF}}$	7.023	12.121	8.216	90	94.25	90	698.63	$P2_1$
This work	$\mathbf{S_{DMSO}}$	10.037	10.865	13.235	90	90	90	1443.24	$P2_12_12_1$
This work	$\mathbf{S_{DMA}}$	9.644	10.518	31.368	90	90	90	3181.81	$P2_12_12_1$
This work	$\mathbf{S_{THF}}$	9.832	11.114	13.163	90	90	90	1438.42	$P2_12_12_1$
This work	$\mathbf{S_{MeOH}}$	12.020	6.986	27.279	90	90	90	2290.50	*Pbca*
This work	$\mathbf{S_{tBuOH}}$	17.734	6.874	12.285	90	93.73	90	1494.36	$P2_1/c$

2. Virtual solid form screening

2.1. Input conformations for computational studies

Based on the potential energy surface scans (Figure S2) and the **AH-II** structure (ZZZAPY03[8]) following conformations were chosen for the CSP studies:

- **Anhydrates Z'=1 and Monohydrates**:
  - ϕ_1=240/ϕ_2=90
  - ϕ_1=80/ϕ_2=10
- **Anhydrates Z'=2**:
  - ϕ_1=240/ϕ_2=90 & ϕ_1=240/ϕ_2=90
  - ϕ_1=80/ϕ_2=10 & ϕ_1=80/ϕ_2=10
  - ϕ_1=240/ϕ_2=90 & ϕ_1=80/ϕ_2=10
  - ϕ_1=240/ϕ_2=90 & ϕ_1=280/ϕ_2=170
  - ϕ_1=120/ϕ_2=90 & ϕ_1=80/ϕ_2=10

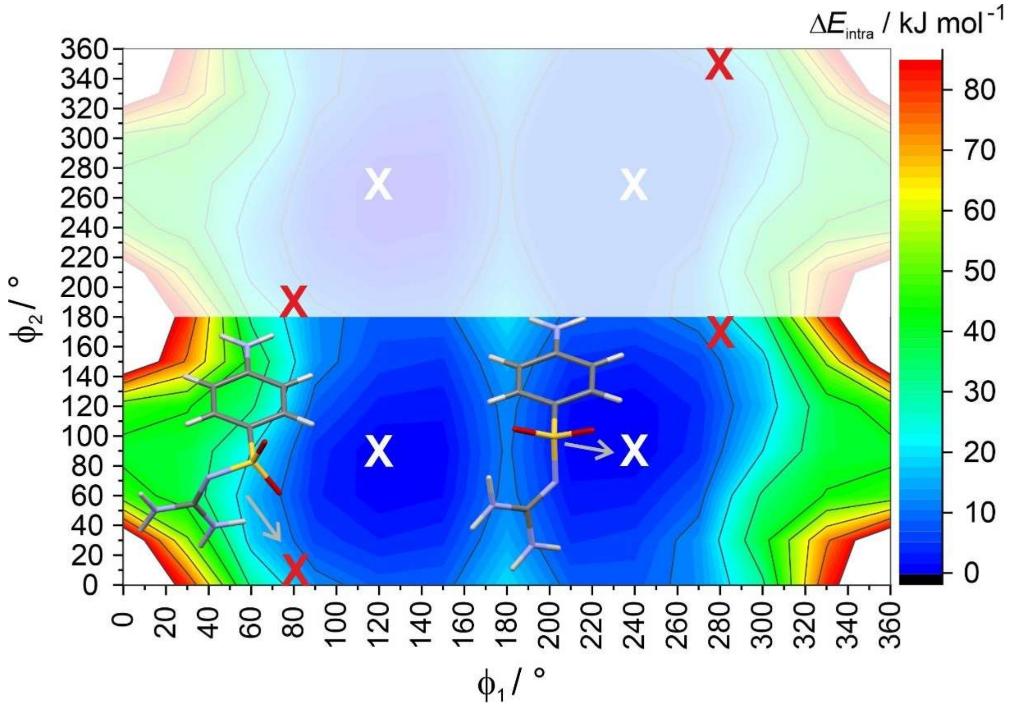

Figure S2. Potential energy surface scan of SGD with search input conformations marked with "X".

2.2. Computational generation of the SGD anhydrate crystal energy landscape

Table S3. Computationally generated low-energy SGD structures. The experimental structures are highlighted in green.

**Str.**	**Space group**	**Cell parameters**						ΔE_{latt}/	**PI**
		a/Å	*b*/Å	*c*/Å	*α*/°	*β*/°	*γ*/°	kJ mol^{-1}	
1 (**II**)	$P2_1/c$	9.837	7.543	23.705	90.00	86.70	90.00	0.00	0.763
2 (**I**)	*P*-1	6.996	10.063	20.237	97.90	95.74	98.99	1.58	0.699
3 (**V**)	$P2_1/c$	10.867	9.847	9.877	90.00	72.52	90.00	1.96	0.663
4	$P2_1/c$	12.038	7.342	22.539	90.00	118.63	90.00	2.27	0.765
5	C2/c	10.832	16.007	11.213	90.00	109.64	90.00	2.38	0.730
6	*Pbca*	11.710	21.731	7.030	90.00	90.00	90.00	5.15	0.754
7	$P2_1/c$	11.805	7.243	22.588	90.00	65.88	90.00	5.18	0.763
8	$Pna2_1$	9.052	20.656	9.955	90.00	90.00	90.00	5.23	0.722
9	$P2_1/c$	13.166	9.439	9.875	90.00	48.62	90.00	5.37	0.731
10	*P*-1	12.330	9.935	7.217	84.48	88.97	86.18	5.53	0.761
11	$P2_1$	6.403	7.217	9.642	90.00	82.31	90.00	5.64	0.764
12	$Pca2_1$	19.060	6.304	7.348	90.00	90.00	90.00	6.05	0.767
13	$P2_1$	10.225	7.210	6.355	90.00	71.39	90.00	6.18	0.757
14	*P*-1	11.196	6.426	14.401	89.99	89.38	103.09	6.34	0.658
15	$Pna2_1$	38.564	6.411	7.208	90.00	90.00	90.00	7.24	0.756
16	$Pna2_1$	19.313	6.445	7.173	90.00	90.00	90.00	7.55	0.756
17	$P2_12_12_1$	9.424	21.007	9.157	90.00	90.00	90.00	7.56	0.744
18	*P*-1	12.814	10.205	7.084	89.75	95.98	73.05	7.76	0.766
19	$P2_1$	10.757	8.125	11.351	90.00	64.76	90.00	8.24	0.749
20	$P2_1/c$	6.354	11.952	12.229	90.00	88.27	90.00	8.32	0.719
21	$P2_1$	5.967	7.600	19.667	90.00	84.74	90.00	8.34	0.757
22	$P2_1$	5.724	7.983	10.010	90.00	84.78	90.00	8.57	0.742
23	Pc	9.811	6.058	15.027	90.00	94.99	90.00	8.98	0.758
24	$Pca2_1$	19.646	12.123	7.670	90.00	90.00	90.00	9.11	0.738
25	C2/c	27.370	6.132	25.959	90.00	125.35	90.00	9.16	0.756
26	$P2_1/c$	22.170	6.975	12.914	90.00	61.16	90.00	9.22	0.768
27	Pbca,	20.561	7.231	24.311	90.00	90.00	90.00	9.25	0.746
28	$Pna2_1$	39.026	6.097	7.505	90.00	90.00	90.00	9.32	0.754
29	$P2_1/c$	6.489	7.135	48.772	90.00	52.24	90.00	9.78	0.747
30	$P2_12_12_1$	9.477	20.766	9.464	90.00	90.00	90.00	9.81	0.721
31	*P*1	9.844	6.043	7.618	90.09	95.61	84.41	10.05	0.755
32	Pbca,	8.327	20.419	10.964	90.00	90.00	90.00	10.22	0.718
33	$P2_1/c$	11.313	11.592	15.496	90.00	66.98	90.00	10.24	0.718
34	$P2_1$	11.052	7.670	11.870	90.00	118.03	90.00	10.37	0.762
35	$P2_1/c$	8.082	9.979	22.886	90.00	90.91	90.00	10.70	0.731
36	*Pbca*	24.952	19.662	7.237	90.00	90.00	90.00	10.90	0.756
37	$Pca2_1$	38.582	6.190	7.614	90.00	90.00	90.00	11.02	0.745
38	$P2_1$	11.801	7.664	9.831	90.00	84.73	90.00	11.06	0.762
39	$P2_1/c$	19.732	7.008	12.769	90.00	85.10	90.00	11.09	0.760
40	*P*-1	11.307	9.917	10.869	124.83	94.24	104.85	11.43	0.725
41	C2/c	43.326	6.939	12.987	90.00	115.48	90.00	11.51	0.765
42	$P2_1/c$	9.783	23.454	7.952	90.00	90.43	90.00	11.58	0.730
43	$P2_1$	19.737	7.696	6.066	90.00	99.27	90.00	11.79	0.743
44	*Pc*	7.974	5.828	19.489	90.00	93.42	90.00	11.88	0.749
45	$P2_1/c$	6.272	20.131	7.207	90.00	94.86	90.00	11.93	0.742
46	$P2_1/c$	12.629	12.114	14.062	90.00	121.71	90.00	12.18	0.738

Str.	Space group	Cell parameters						ΔE_{latt}/ kJ mol^{-1}	PI
		a/Å	b/Å	c/Å	α/°	β/°	γ/°		
47	Pc	12.411	7.132	9.910	90.00	86.98	90.00	12.29	0.772
48	$P2_1/c$	19.829	7.191	12.377	90.00	86.51	90.00	12.32	0.762
49	$P2_1/c$	10.051	19.274	9.711	90.00	92.74	90.00	12.32	0.714
50	P-1	10.051	7.463	15.121	113.88	119.19	85.77	12.35	0.751
51	$P2_1/c$	19.239	10.031	9.676	90.00	90.80	90.00	12.64	0.720
52	$P2_1$	7.892	11.747	10.042	90.00	86.56	90.00	12.65	0.721
53	$Pbca$	24.374	10.400	14.229	90.00	90.00	90.00	12.71	0.749
53	$Pca2_1$	18.824	9.847	9.637	90.00	90.00	90.00	12.80	0.756
55	$P2_1/c$	8.197	9.992	22.702	90.00	92.60	90.00	12.83	0.725
56	$P2_1/c$	15.442	11.488	10.311	90.00	84.21	90.00	12.86	0.740
57	Cc	22.449	7.036	12.471	90.00	117.95	90.00	13.05	0.773
58	$Pbca$	19.570	7.274	12.464	90.00	90.00	90.00	13.10	0.755
59	Cc	7.621	38.950	6.106	90.00	89.06	90.00	13.17	0.749
60	$Pna2_1$	39.484	6.168	7.539	90.00	90.00	90.00	13.38	0.734
61	$P2_1/c$	8.174	12.924	8.520	90.00	96.77	90.00	13.45	0.752
62	$Pca2_1$	12.584	7.096	19.649	90.00	90.00	90.00	13.49	0.769
63	$Pna2_1$	39.288	6.328	7.369	90.00	90.00	90.00	13.55	0.736
64	$Pbca$	12.768	38.437	7.212	90.00	90.00	90.00	13.75	0.757
65	$P2_1$	11.152	8.440	9.744	90.00	84.90	90.00	13.76	0.740
66	$P2_1/c$	20.688	7.133	12.598	90.00	71.27	90.00	14.05	0.761
67	Pc	6.157	19.680	7.584	90.00	89.08	90.00	14.07	0.735
68	$P2_1/c$	6.304	38.971	7.358	90.00	87.41	90.00	14.11	0.744
69	$P2_1/c$	10.798	7.240	11.997	90.00	70.04	90.00	14.44	0.762
70	$P2_1/c$	6.426	7.281	39.295	90.00	85.03	90.00	14.63	0.730
71	$Pbca$	39.607	7.023	12.917	90.00	90.00	90.00	14.94	0.747

2.3. Representation of the experimental SGD anhydrate structures

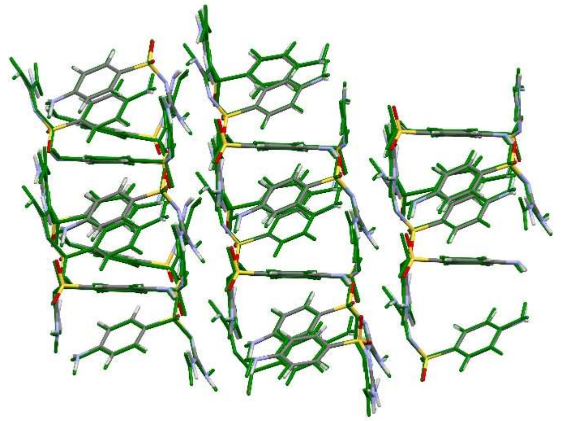

Figure S3. Overlay of a 30-molecule cluster of the observed structure of SGD **AH-I** (colored by element) and calculated PBE-MBD* structure (green), $rmsd_{30}^{11}$=0.25 Å.

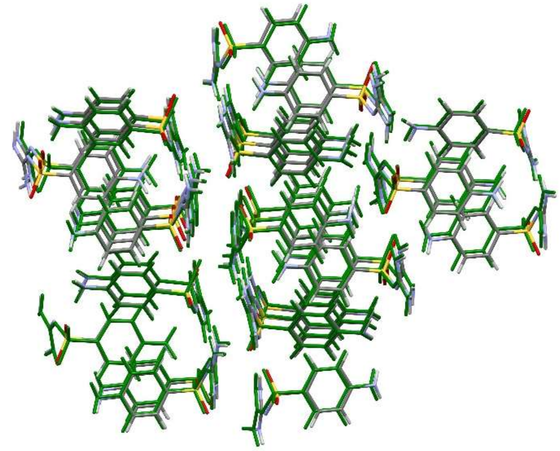

Figure S4. Overlay of a 30-molecule cluster of the observed structure of SGD **AH-II** (colored by element) and calculated PBE-MBD* structure (green), $rmsd_{30}$=0.20 Å.

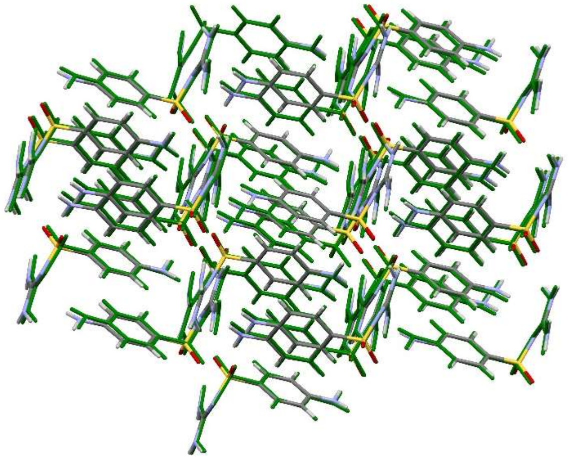

Figure S5. Overlay of a 30-molecule cluster of the observed structure of SGD **AH-V** (colored by element) and calculated PBE-MBD* structure (green), $rmsd_{30}$=0.22 Å.

2.4. CSP: AH-II cluster and potential AH-IV structures

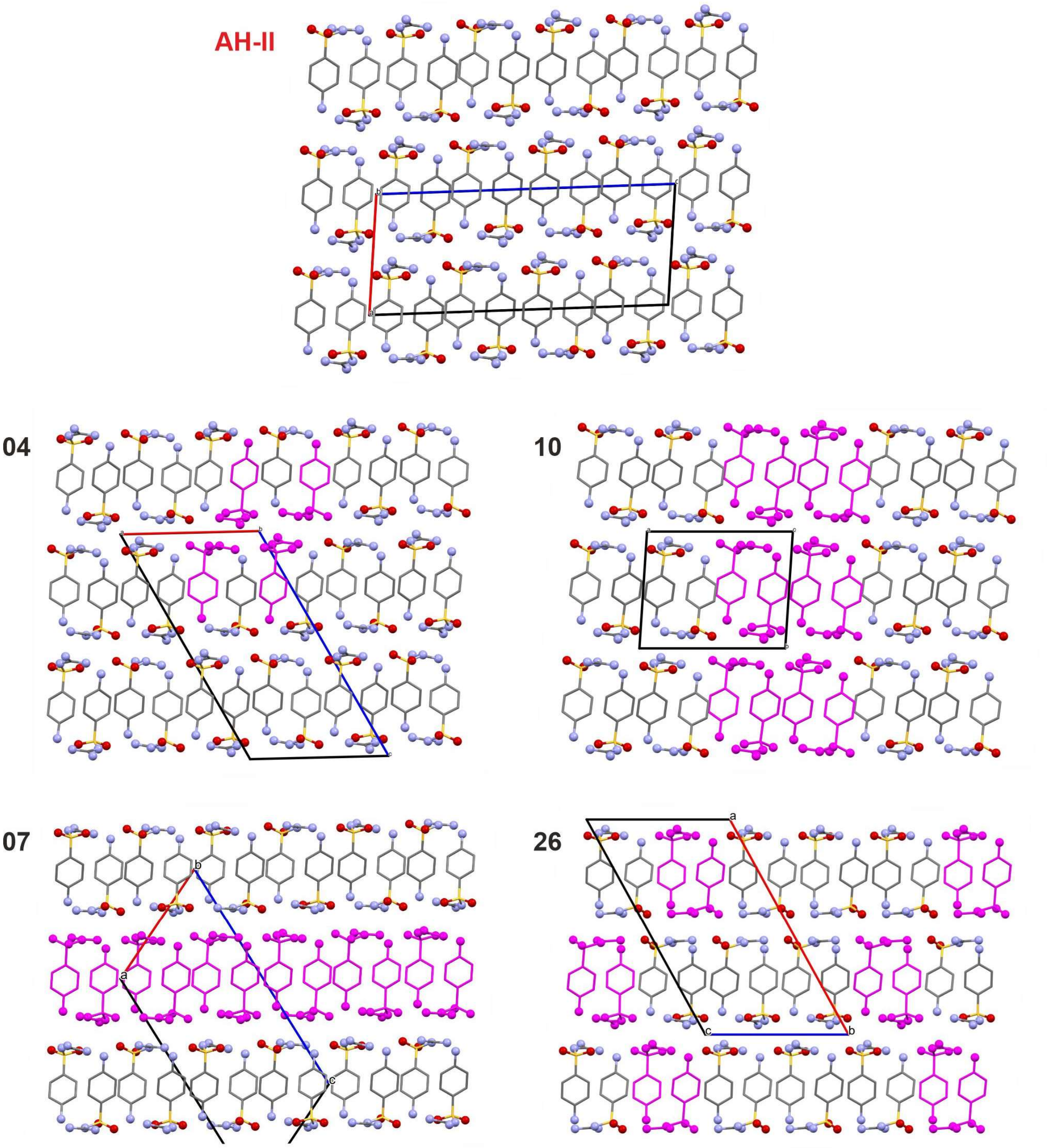

Figure S6. Packing diagrams of the **AH-II** and closely related structures. With **AH-II** common packing motifs are highlighted in magenta in structures *04*, *07*, *10,* and *26*.

The .res files for the potential **AH-IV** structures are provided below.

2.4.1. Structure *04*

```
TITL SGD_AH_04
CELL 0.71073 12.0378 7.34174 22.5389 90 118.635 90
ZERR 4 0 0 0 0 0 0
LATT 1
SYMM -x,1/2+y,1/2-z
SFAC C H N O S
C1     1  0.499080  0.144692  0.286479  1.000000 0.010000
C2     1  0.437725  0.170503  0.325150  1.000000 0.010000
C3     1  0.306402  0.149941  0.295056  1.000000 0.010000
C4     1  0.237221  0.100042  0.227575  1.000000 0.010000
C5     1  0.298939  0.063339  0.189438  1.000000 0.010000
C6     1  0.430534  0.090734  0.219707  1.000000 0.010000
C7     1  0.664859 -0.055863  0.434018  1.000000 0.010000
C8     1  0.817588  0.377162  0.783772  1.000000 0.010000
C9     1  0.942331  0.313449  0.816446  1.000000 0.010000
C10    1  0.992887  0.229321  0.778928  1.000000 0.010000
C11    1  0.917959  0.202231  0.710224  1.000000 0.010000
C12    1  0.789464  0.251506  0.678116  1.000000 0.010000
C13    1  0.742067  0.347266  0.715134  1.000000 0.010000
C14    1  1.125331  0.014429  0.948467  1.000000 0.010000
H1     2  0.258961  0.168137  0.325369  1.000000 0.010000
H2     2  0.135043  0.082578  0.204930  1.000000 0.010000
H3     2  0.478114  0.065605  0.189865  1.000000 0.010000
H4     2  0.600912  0.164724  0.308877  1.000000 0.010000
H5     2  0.556584 -0.138331  0.475898  1.000000 0.010000
H6     2  0.598492 -0.310895  0.439216  1.000000 0.010000
H7     2  0.768725 -0.259644  0.418848  1.000000 0.010000
H8     2  0.819279 -0.034504  0.418760  1.000000 0.010000
H9     2  0.143415 -0.046700  0.105405  1.000000 0.010000
H10    2  0.285630 -0.059697  0.104419  1.000000 0.010000
H11    2  1.090690  0.182686  0.803356  1.000000 0.010000
H12    2  0.957243  0.136250  0.681217  1.000000 0.010000
H13    2  0.644447  0.395000  0.689464  1.000000 0.010000
H14    2  0.780001  0.451072  0.812148  1.000000 0.010000
H15    2  1.247404  0.161916  0.928202  1.000000 0.010000
H16    2  1.271966 -0.079339  0.933805  1.000000 0.010000
H17    2  1.035910 -0.163915  0.981687  1.000000 0.010000
H18    2  1.182421 -0.239393  0.992659  1.000000 0.010000
H19    2  0.617882  0.194461  0.598088  1.000000 0.010000
H20    2  0.743705  0.102889  0.593209  1.000000 0.010000
N1     3  0.659227  0.128164  0.439604  1.000000 0.010000
N2     3  0.754313 -0.121092  0.421343  1.000000 0.010000
N3     3  0.232874  0.007352  0.122451  1.000000 0.010000
N4     3  0.583536 -0.173014  0.440264  1.000000 0.010000
N5     3  1.039423  0.146486  0.939363  1.000000 0.010000
N6     3  1.107378 -0.149524  0.969537  1.000000 0.010000
N7     3  0.711363  0.209574  0.610491  1.000000 0.010000
N8     3  1.226108  0.034925  0.938623  1.000000 0.010000
O1     4  0.449748  0.180738  0.443350  1.000000 0.010000
O2     4  0.557420  0.425635  0.415887  1.000000 0.010000
O3     4  1.161870  0.397695  0.917075  1.000000 0.010000
O4     4  0.968830  0.464179  0.926202  1.000000 0.010000
S1     5  0.526562  0.231259  0.411419  1.000000 0.010000
S2     5  1.035829  0.339395  0.904548  1.000000 0.010000
END
```

2.4.2. Structure *07*

```
TITL SGD_AH_07
CELL 0.71073 11.8047 7.243 22.5876 90 65.8758 90
ZERR 4 0 0 0 0 0 0
LATT 1
SYMM -x,1/2+y,1/2-z
SFAC C H N O S
C1     1  0.269167  0.170583  0.335281  1.000000 0.010000
C2     1  0.323404  0.229656  0.270815  1.000000 0.010000
C3     1  0.451745  0.256293  0.240507  1.000000 0.010000
C4     1  0.525277  0.223784  0.274016  1.000000 0.010000
C5     1  0.471882  0.159158  0.338235  1.000000 0.010000
C6     1  0.342703  0.135149  0.368566  1.000000 0.010000
C7     1  0.086518  0.003990  0.271786  1.000000 0.010000
C8     1  0.945469  0.266577 -0.001773  1.000000 0.010000
C9     1  0.816168  0.245809  0.026903  1.000000 0.010000
C10    1  0.759438  0.174608  0.089972  1.000000 0.010000
C11    1  0.830456  0.116128  0.122061  1.000000 0.010000
C12    1  0.960785  0.124751  0.092176  1.000000 0.010000
C13    1  1.016888  0.209459  0.030777  1.000000 0.010000
C14    1  0.640892 -0.060390 -0.005212  1.000000 0.010000
H1     2  0.494203  0.302775  0.190353  1.000000 0.010000
H2     2  0.625214  0.245981  0.250332  1.000000 0.010000
H3     2  0.300170  0.086406  0.418485  1.000000 0.010000
H4     2  0.169213  0.151046  0.359383  1.000000 0.010000
H5     2  0.226743 -0.060020  0.184206  1.000000 0.010000
H6     2  0.146712 -0.253026  0.229827  1.000000 0.010000
H7     2 -0.024685 -0.202847  0.330683  1.000000 0.010000
H8     2 -0.066578  0.026166  0.357773  1.000000 0.010000
H9     2  0.636942  0.098946  0.344110  1.000000 0.010000
H10    2  0.508093  0.026154  0.408133  1.000000 0.010000
H11    2  0.659119  0.161703  0.114006  1.000000 0.010000
H12    2  0.785602  0.060129  0.170881  1.000000 0.010000
H13    2  1.117438  0.226129  0.008027  1.000000 0.010000
H14    2  0.990028  0.326348 -0.050104  1.000000 0.010000
H15    2  0.523909  0.097541  0.069660  1.000000 0.010000
H16    2  0.498539 -0.144966  0.080304  1.000000 0.010000
H17    2  0.712597 -0.248575 -0.078189  1.000000 0.010000
H18    2  0.601810 -0.337394 -0.006811  1.000000 0.010000
H19    2  1.120759  0.020082  0.096995  1.000000 0.010000
H20    2  0.984925 -0.033204  0.161698  1.000000 0.010000
N1     3  0.099544  0.191191  0.268089  1.000000 0.010000
N2     3 -0.007275 -0.062798  0.323703  1.000000 0.010000
N3     3  0.544832  0.125200  0.372646  1.000000 0.010000
N4     3  0.162286 -0.113403  0.226560  1.000000 0.010000
N5     3  0.713941  0.072836 -0.044229  1.000000 0.010000
N6     3  0.663781 -0.234181 -0.028939  1.000000 0.010000
N7     3  1.029728  0.059000  0.124843  1.000000 0.010000
N8     3  0.551695 -0.034256  0.054424  1.000000 0.010000
O1     4  0.297333  0.210424  0.161892  1.000000 0.010000
O2     4  0.211365  0.482697  0.230824  1.000000 0.010000
O3     4  0.604083  0.350475  0.024756  1.000000 0.010000
O4     4  0.795522  0.385280 -0.075431  1.000000 0.010000
S1     5  0.230547  0.282374  0.228150  1.000000 0.010000
S2     5  0.726070  0.274873 -0.018374  1.000000 0.010000
END
```

2.4.3. Structure *10*

```
TITL SGD_AH_10
CELL 0.71073 12.3299 9.93473 7.21712 84.4791 88.9673 86.1823
ZERR 2 0 0 0 0 0 0
LATT 1
SFAC C H N O S
C1     1  0.301677  0.421069  0.828828  1.000000 0.010000
C2     1  0.393516  0.343206  0.775360  1.000000 0.010000
C3     1  0.491451  0.404813  0.739412  1.000000 0.010000
C4     1  0.497940  0.541521  0.757154  1.000000 0.010000
C5     1  0.406293  0.620292  0.812995  1.000000 0.010000
C6     1  0.307968  0.557873  0.847389  1.000000 0.010000
C7     1  0.267542  0.125334  1.033419  1.000000 0.010000
C8     1  0.022528  0.560794  0.227410  1.000000 0.010000
C9     1  0.119118  0.624267  0.246631  1.000000 0.010000
C10    1  0.209634  0.549578  0.325669  1.000000 0.010000
C11    1  0.202456  0.415417  0.391247  1.000000 0.010000
C12    1  0.104015  0.352784  0.384112  1.000000 0.010000
C13    1  0.015092  0.425777  0.294130  1.000000 0.010000
C14    1  0.185153  0.883866  0.503847  1.000000 0.010000
H1     2  0.563173  0.343784  0.699730  1.000000 0.010000
H2     2  0.574966  0.588434  0.732339  1.000000 0.010000
H3     2  0.236731  0.617657  0.892294  1.000000 0.010000
H4     2  0.224921  0.374604  0.857383  1.000000 0.010000
H5     2  0.417864  0.055493  1.126971  1.000000 0.010000
H6     2  0.334831  0.121689  1.294961  1.000000 0.010000
H7     2  0.146348  0.143767  1.235150  1.000000 0.010000
H8     2  0.101816  0.151342  1.003586  1.000000 0.010000
H9     2  0.488956  0.786670  0.847858  1.000000 0.010000
H10    2  0.357383  0.795578  0.925059  1.000000 0.010000
H11    2  0.285651  0.597349  0.337196  1.000000 0.010000
H12    2  0.272885  0.357121  0.452749  1.000000 0.010000
H13    2 -0.060696  0.377082  0.280556  1.000000 0.010000
H14    2 -0.046927  0.617875  0.160494  1.000000 0.010000
H15    2  0.321216  0.851258  0.343361  1.000000 0.010000
H16    2  0.345660  0.854258  0.585868  1.000000 0.010000
H17    2  0.069609  0.938200  0.694914  1.000000 0.010000
H18    2  0.202291  0.969985  0.750967  1.000000 0.010000
H19    2  0.024049  0.187707  0.506639  1.000000 0.010000
H20    2  0.159787  0.185734  0.550578  1.000000 0.010000
N1     3  0.282274  0.117387  0.847095  1.000000 0.010000
N2     3  0.165763  0.143015  1.094614  1.000000 0.010000
N3     3  0.411763  0.757818  0.830022  1.000000 0.010000
N4     3  0.350637  0.117924  1.154951  1.000000 0.010000
N5     3  0.107052  0.870844  0.378519  1.000000 0.010000
N6     3  0.151145  0.920859  0.671655  1.000000 0.010000
N7     3  0.099181  0.218821  0.457991  1.000000 0.010000
N8     3  0.292801  0.863914  0.475087  1.000000 0.010000
O1     4  0.491927  0.103593  0.815328  1.000000 0.010000
O2     4  0.372880  0.156684  0.545018  1.000000 0.010000
O3     4  0.238118  0.819013  0.112861  1.000000 0.010000
O4     4  0.038397  0.847085  0.067912  1.000000 0.010000
S1     5  0.389328  0.169123  0.742417  1.000000 0.010000
S2     5  0.128598  0.799292  0.188807  1.000000 0.010000
END
```

2.4.4. Structure *26*

```
TITL SG_AH_26
CELL 0.71073 22.1703 6.97531 12.914 90 61.1569 90
ZERR 4 0 0 0 0 0 0
LATT 1
SYMM -x,1/2+y,1/2-z
SFAC C H N O S
H1     2  0.691190  0.833528  0.239635  1.000000 0.010000
H2     2  0.814005  0.747151  0.120310  1.000000 0.010000
H3     2  0.818808  0.580495  0.437516  1.000000 0.010000
H4     2  0.696808  0.666705  0.556605  1.000000 0.010000
H5     2  0.557451  0.428357  0.534748  1.000000 0.010000
H6     2  0.561528  0.282743  0.643289  1.000000 0.010000
H7     2  0.551467  0.418411  0.810519  1.000000 0.010000
H8     2  0.561486  0.666458  0.821202  1.000000 0.010000
H9     2  0.911225  0.560584  0.118784  1.000000 0.010000
H10    2  0.908745  0.505463  0.249098  1.000000 0.010000
C1     1  0.721818  0.682013  0.460999  1.000000 0.010000
C2     1  0.685105  0.749453  0.405881  1.000000 0.010000
C3     1  0.718959  0.775352  0.282955  1.000000 0.010000
C4     1  0.787972  0.727866  0.215947  1.000000 0.010000
C5     1  0.824514  0.651744  0.270431  1.000000 0.010000
C6     1  0.790621  0.634762  0.394157  1.000000 0.010000
C7     1  0.566617  0.573024  0.664986  1.000000 0.010000
N1     3  0.573579  0.755276  0.623900  1.000000 0.010000
N2     3  0.562136  0.550527  0.771377  1.000000 0.010000
N3     3  0.893706  0.602432  0.205904  1.000000 0.010000
N4     3  0.564251  0.415331  0.607142  1.000000 0.010000
O1     4  0.560254  0.682008  0.442228  1.000000 0.010000
O2     4  0.586713  1.008166  0.482205  1.000000 0.010000
S1     5  0.596373  0.803159  0.489423  1.000000 0.010000
H11    2  0.194012  0.681666  1.098921  1.000000 0.010000
H12    2  0.317508  0.606006  0.991368  1.000000 0.010000
H13    2  0.321378  0.728687  0.660992  1.000000 0.010000
H14    2  0.196715  0.804895  0.769958  1.000000 0.010000
H15    2  0.082134  0.594164  1.218800  1.000000 0.010000
H16    2  0.074190  0.345441  1.253654  1.000000 0.010000
H17    2  0.028533  0.220302  1.033647  1.000000 0.010000
H18    2  0.041253  0.133370  1.147624  1.000000 0.010000
H19    2  0.414150  0.559614  0.673147  1.000000 0.010000
H20    2  0.409144  0.494012  0.802304  1.000000 0.010000
C8     1  0.223366  0.758534  0.817702  1.000000 0.010000
C9     1  0.186726  0.741103  0.940829  1.000000 0.010000
C10    1  0.221367  0.688661  1.002900  1.000000 0.010000
C11    1  0.290790  0.646314  0.942653  1.000000 0.010000
C12    1  0.327253  0.652033  0.818423  1.000000 0.010000
C13    1  0.293121  0.716136  0.757054  1.000000 0.010000
C14    1  0.061851  0.424398  1.110020  1.000000 0.010000
N5     3  0.064641  0.556586  1.031282  1.000000 0.010000
N6     3  0.044383  0.246188  1.094723  1.000000 0.010000
N7     3  0.396429  0.599663  0.759542  1.000000 0.010000
N8     3  0.075210  0.456537  1.199636  1.000000 0.010000
O3     4  0.078703  0.848608  1.134773  1.000000 0.010000
O4     4  0.075594  0.875681  0.945170  1.000000 0.010000
S2     5  0.096559  0.766340  1.018256  1.000000 0.010000
END
```

2.5. Computational generation of the SGD monohydrate crystal energy landscape

Table S4. Computationally generated low-energy SGD monohydrate structures. The experimental structures are highlighted in green.

Str.	Space group	Cell parameters						ΔE_{latt}/ kJ mol^{-1}	PI
		a/Å	*b*/Å	*c*/Å	*α*/°	*β*/°	*γ*/°		
1 (**Hy1-II**)	$P2_1$	5.486	7.310	12.320	90.00	92.17	90.00	0.00	0.750
2 (**Hy1-I**)	$P2_1/c$	7.432	5.435	24.165	90.00	91.78	90.00	1.70	0.749
3	$P2_1/c$	5.417	7.424	24.474	90.00	99.33	90.00	1.80	0.755
4	*Pbca*	24.134	11.832	6.924	90.00	90.00	90.00	1.91	0.743
5	$P2_1/c$	5.470	7.341	24.583	90.00	92.35	90.00	1.97	0.745
6	$P2_1$	12.103	5.437	7.403	90.00	89.90	90.00	3.10	0.756
7	$P2_12_12_1$	24.160	7.399	5.436	90.00	90.00	90.00	4.89	0.759
8	$P2_1/c$	7.557	5.443	23.942	90.00	91.68	90.00	6.10	0.739
9	$P2_12_12_1$	23.999	7.340	5.484	90.00	90.00	90.00	6.93	0.761
10	$P2_1/c$	11.885	7.514	12.528	90.00	65.59	90.00	8.80	0.719
11	$P2_1/c$	11.057	9.521	9.396	90.00	83.07	90.00	9.07	0.754
12	$Pca2_1$	24.247	5.416	7.396	90.00	90.00	90.00	9.32	0.766
13	$P2_1/c$	12.093	5.522	14.948	90.00	90.30	90.00	9.71	0.727
14	$P2_1/c$	12.095	5.495	15.184	90.00	93.58	90.00	10.44	0.719
15	C2/c,	23.703	5.448	15.015	90.00	92.83	90.00	10.65	0.761
16	$P2_1/c$	13.670	5.443	14.996	90.00	120.25	90.00	10.95	0.770
17	C2	23.939	5.460	7.423	90.00	85.77	90.00	11.28	0.757
18	*Pbca*	27.616	8.247	8.646	90.00	90.00	90.00	11.30	0.746
19	*P*-1	5.433	7.549	12.166	84.61	92.34	88.36	11.62	0.732
20	$P2_1/c$	13.835	5.482	15.265	90.00	59.70	90.00	11.80	0.730
21	$P2_1/c$	6.705	7.081	23.775	90.00	67.97	90.00	12.57	0.703
22	*Pbcn*	24.339	8.468	9.808	90.00	90.00	90.00	13.03	0.725
23	$P2_1/c$	11.982	6.910	17.694	90.00	43.36	90.00	13.31	0.729
24	$P2_1/c$	12.123	5.504	14.873	90.00	92.44	90.00	13.75	0.736
25	$P2_1/c$	13.653	8.883	8.444	90.00	92.76	90.00	13.76	0.718
26	$P2_1/c$	12.110	7.444	12.277	90.00	69.20	90.00	14.08	0.704
27	*P*-1	9.432	6.172	10.145	116.37	89.46	89.36	14.15	0.688
28	$Pna2_1$	7.388	24.034	5.502	90.00	90.00	90.00	14.43	0.755
29	$P2_1/c$	12.077	5.579	14.625	90.00	100.08	90.00	15.25	0.763
30	*Pbca*	10.903	7.573	24.482	90.00	90.00	90.00	15.46	0.725
31	$P2_1/c$	10.363	12.371	7.941	90.00	86.04	90.00	15.50	0.725
32	$P2_1/c$	11.552	7.222	12.193	90.00	76.96	90.00	15.83	0.749
33	C2/c,	12.044	7.494	24.654	90.00	69.43	90.00	16.07	0.703
34	$P2_1/c$	12.163	5.486	14.758	90.00	99.06	90.00	16.16	0.758
35	*P*-1	7.432	12.412	6.996	87.47	61.13	67.44	16.27	0.711
36	$P2_1/c$	11.363	8.114	11.941	90.00	117.92	90.00	16.44	0.760
37	C2/c	24.071	5.501	15.243	90.00	88.34	90.00	16.80	0.733
38	*Pbca*	7.424	12.222	22.951	90.00	90.00	90.00	17.01	0.705
39	*Pbcn*	6.378	12.463	24.975	90.00	90.00	90.00	17.18	0.743
40	$P2_1/c$	12.001	5.502	15.254	90.00	87.75	90.00	17.37	0.734
41	*Pbca*	11.801	23.827	7.092	90.00	90.00	90.00	17.45	0.743
42	*Pbca*	7.216	21.867	12.690	90.00	90.00	90.00	17.48	0.740

Str.	Space group	Cell parameters						ΔE_{latt}/	PI
		a/Å	*b*/Å	*c*/Å	*α*/°	*β*/°	*γ*/°	kJ mol^{-1}	
43	C2	15.371	5.495	14.070	90.00	58.52	90.00	17.49	0.723
44	*Pbca*	22.375	7.443	12.194	90.00	90.00	90.00	17.50	0.723
45	*P*2$_1$/*c*	6.152	6.787	27.019	90.00	63.16	90.00	17.55	0.730
46	*P*-1	12.088	6.037	7.525	74.13	78.84	91.98	17.60	0.714
47	C2/c	28.166	5.487	15.286	90.00	58.76	90.00	17.96	0.732
48	*Pbca*	13.741	7.572	18.967	90.00	90.00	90.00	17.99	0.745
49	*P*-1	6.031	11.944	7.589	85.47	105.99	86.16	18.63	0.698
50	C2/c	18.985	6.082	17.932	90.00	86.91	90.00	19.16	0.708

2.6. Representation of the experimental monohydrate structures

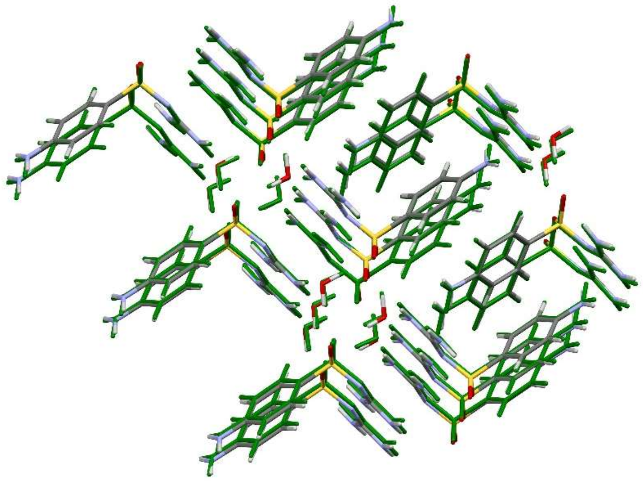

Figure S7. Overlay of a 30-molecule cluster of the observed structure of SGD **Hy1-I** (colored by element) and calculated PBE-MBD* structure (green), *rmsd*$_{30}$=0.15 Å.

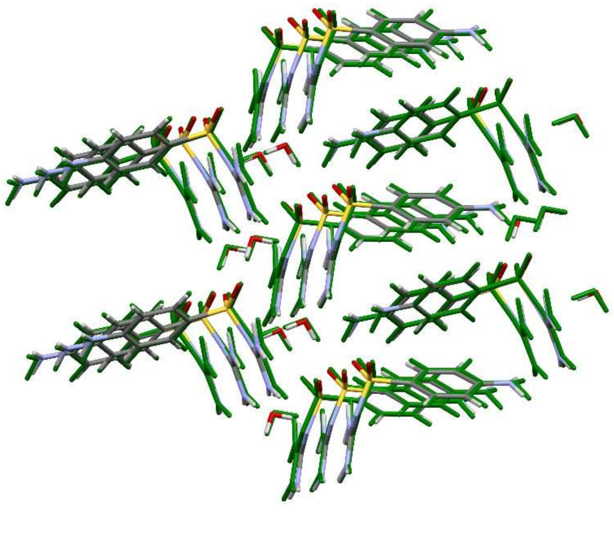

Figure S8. Overlay of a 30-molecule cluster of the observed structure of SGD **Hy1-II** (colored by element) and calculated PBE-MBD* structure (green), *rmsd*$_{30}$=0.20 Å.

2.7. Multi-component hydrogen-bond propensity screen for SGD solvates

Table S5. Multi-component hydrogen-bond propensity screen results: SGD – sulfaguanidine and S – solvent. In green the experimentally observed solvates (and hydrate).

Rank	Solvent (C)	Multi-component score	Max interaction	Max SGD:S propensity	Max SGD:SGD propensity	Max S:S propensity
1	Dimethyl sulfoxide	0.14	SGD:S	0.99	0.85	0.14
2	*N,N*-Dimethyl formamide	0.13	SGD:S	0.98	0.85	0.13
3	Tetrahydrofurane	0.09	SGD:S	0.92	0.83	0.09
4	*N,N*-Dimethyl acetamide	0.06	SGD:S	0.92	0.86	0.06
5	1,4-Dioxane	0.05	SGD:S	0.89	0.83	0.05
6	Acetone	0.05	SGD:S	0.87	0.83	0.05
7	Cyclohexanone	0.02	SGD:S	0.84	0.82	0.02
8	Ethyl acetate	0.02	SGD:S	0.85	0.82	0.02
9	Acetonitrile	0.0	SGD:S	0.83	0.83	0.0
10	Nitromethane	0.0	SGD:S	0.83	0.83	0.0
11	Water	−0.01	SGD:SGD	0.75	0.76	−0.01
12	Methyl ethyl ketone	−0.04	SGD:SGD	0.81	0.85	−0.04
13	*i*-Propanol	−0.05	SGD:SGD	0.77	0.82	−0.05
14	Methyl *i*-butyl ketone	−0.09	SGD:SGD	0.76	0.85	−0.09
15	Butyl acetate	−0.1	SGD:SGD	0.73	0.83	−0.1
16	Glycerol	−0.1	SGD:SGD	0.68	0.78	−0.1
17	Isobutyl acetate	−0.12	SGD:SGD	0.71	0.83	−0.12
18	Dimethyl carbonate	−0.13	SGD:SGD	0.67	0.8	−0.13
19	Ethanol	−0.13	SGD:SGD	0.67	0.81	−0.13
20	Ethylene glycol	−0.15	SGD:SGD	0.64	0.79	−0.15
21	Methanol	−0.15	SGD:SGD	0.65	0.8	−0.15
22	Diethylene glycol	−0.16	SGD:SGD	0.66	0.82	−0.16
23	Diethyl ether	−0.16	SGD:SGD	0.65	0.81	−0.16
24	*n*-Propanol	−0.18	SGD:SGD	0.61	0.79	−0.18
25	*n*-Butanol	−0.2	SGD:SGD	0.59	0.79	−0.2
26	*n*-Decanol	−0.21	SGD:SGD	0.58	0.79	−0.21
27	*n*-Octanol	−0.21	SGD:SGD	0.58	0.79	−0.21
28	*i*-Butanol	−0.21	SGD:SGD	0.58	0.79	−0.21
29	*t*-Butyl methyl ether	−0.21	SGD:SGD	0.6	0.81	−0.21
30	*i*-Pentanol	−0.22	SGD:SGD	0.57	0.79	−0.22
31	*t*-Butanol	−0.23	SGD:SGD	0.56	0.79	−0.23
32	*n*-Pentanol	−0.24	SGD:SGD	0.55	0.79	−0.24
33	*t*-Pentanol	−0.27	SGD:SGD	0.52	0.79	−0.27
34	Di-*i*-propyl ether	−0.28	SGD:SGD	0.53	0.81	−0.28
35	Dichloromethane	−0.69	SGD:SGD	0.12	0.81	−0.69
36	Chloroform	−0.71	SGD:SGD	0.11	0.82	−0.71

Rank	Coformer (C)	Multi-component score	Max interaction	Max SGD:S propensity	Max SGD:SGD propensity	Max S:S propensity
37	*n*-Heptane	−0.84	SGD:SGD	0.0	0.84	−0.84
38	*n*-Hexane	−0.84	SGD:SGD	0.0	0.84	−0.84
39	Toluene	−0.84	SGD:SGD	0.0	0.84	−0.84
40	Xylene	−0.84	SGD:SGD	0.0	0.84	−0.84
41	Cyclohexane	−0.85	SGD:SGD	0.0	0.85	−0.85

3. Experimental solid form screening

3.1. Solvent evaporation experiments

A saturated solution of SGD **AH-I** (15 mg) was prepared at RT. The solution was filtered, transferred onto a watch glass and then allowed to evaporate at ambient conditions. The crystallization products were analyzed using PXRD.

Three novel solid-state forms resulted from the experiments (Table S6), *i.e.* **AH-V** and **two solvates** (cyclohexanone and DMSO).

Table S6. SGD solvent evaporation experiments.

Solvent (mL)	Solid-state form[a]	Solvent (mL)	Solid-state form[a]
methanol (2)	**Hy1-I**	methyl *i*-butyl ketone (20)	**Hy1-I**
ethanol (16)	**Hy1-I**	ethyl acetate (>20)	**Hy1-I**
n-propanol (>20)	**Hy1-I**	butyl acetate (>20)	**Hy1-I**
i-propanol (20)	**Hy1-I**	*i*-butyl acetate (>20)	**Hy1-I**
n-butanol (>20)	**Hy1-I**	dimethyl acetamide (0.4)	**Hy1-I**
t-butanol (>20)	**Hy1-I**	dimethyl carbonate (20)	**Hy1-I**
water (20)	**Hy1-I**	nitromethane (20)	**AH-I**
diethyl ether (>20)	—[b]	acetonitrile (6)	**Hy1-I**
di-*i*-propyl ether (>20)	—[b]	dimethyl sulfoxide (0.2)	**S-DMSO**
methyl-*t*-butyl ether (20)	—[b]	dimethyl formamide (0.1)	**Hy1-I**
tetrahydrofurane (20)	**AH-V** (pseudomorphosis)	dichloromethane (>20)	—[b]
1,4-dioxane (>20)	Amorphous	chloroform (>20)	—[b]
acetone (9.5)	**AH-I + AH-II**	*n*-heptane (>20)	—[b]
cyclohexanone (>20)	**S-CHXO**	toluene (>20)	—[b]
methyl ethyl ketone (10)	**Hy1-I**	cyclohexane (>20)	—[b]

[a] AH – anhydrate, Hy – hydrate, S – solvate; [b] not enough material (solubility too low).

3.2. Liquid-assisted grinding experiments

To 80 mg of SGD **AH-I**, eight drops of solvent (in case of dimethyl acetamide and diethylene glycol: two drops) were added, and the resulting paste was milled in stainless steel vessels with three balls of the same material and 0.5 cm in diameter using a Retsch ball mill MM 500 (Haan, Germany) at 15 Hz for 30 min. The resulting samples were analyzed using PXRD.

The liquid-assisted grinding experiments produced **six novel** solid-state forms (Table S7) – all of them **solvates** (*t*-butanol, cyclohexanone, DMSO, DMF, DMA, and THF). The fact that no other anhydrate, except **AH-I**, emerged from the experiments confirms that **AH-I** shows high kinetic stability in the investigated temperature range of 25 – 40 °C.

Table S7. SGD grinding experiments in water and organic solvents.

Solvent	Solid-state form[a]	Solvent	Solid-state form[a]
methanol	**AH-I**	acetone	**AH-I** + **Hy1-I**
ethanol	**AH-I** + **Hy1-I**	cyclohexanone	**S-CHXO** + **AH-I** + **Hy1-I**
n-propanol	**AH-I** + **Hy1-I**	methyl ethyl ketone	**AH-I**
i-propanol	**AH-I**	methyl *i*-butyl ketone	**AH-I**
n-butanol	**AH-I**	ethyl acetate	**AH-I**
i-butanol	**AH-I**	butyl acetate	**AH-I**
t-butanol	**S-*t*BuOH**	*i*-butyl acetate	**AH-I**
n-pentanol	**AH-I**	dimethyl carbonate	**AH-I**
i-pentanol	**AH-I**	nitromethane	**AH-I**
t-pentanol	**AH-I**	acetonitrile	**AH-I** + **Hy1-I**
n-octanol	**AH-I**	dimethyl sulfoxide	**S-DMSO**
n-decanol	**AH-I**	dimethyl formamide	**S-DMF**
glycerol	**Hy1-I**	dimethyl acetamide	**S-DMA**
water	**Hy1-I**	dichloromethane	**AH-I**
ethylene glycol	**AH-I**	chloroform	**AH-I**
diethylene glycol	**AH-I**	*n*-hexane	**AH-I**
diethyl ether	**AH-I**	*n*-heptane	**AH-I**
di-*i*-propyl ether	**AH-I**	cyclohexane	**Hy1-I**
methyl-*t*-butyl ether	**AH-I**	toluene	**AH-I**
tetrahydrofuran	**S-THF** + **AH-I** + **Hy1-I**	xylene	**AH-I**
1,4-dioxane	**AH-I** + **Hy1-I**		

[a] AH – anhydrate, Hy – hydrate, S – solvate.

3.3. Slurry experiments

To 100 mg of SGD **AH-I** solvent was added and the mixture was stirred (700 rpm) in a cycling temperature range between 10 °C and 30 °C. Samples were periodically withdrawn and analyzed using PXRD (Table S8).

Overall, **seven new** solid-state forms (all **solvates**) could be obtained phase pure. Furthermore, the results indicate that **AH-I** slowly transformed to **Hy1-I** in a considerable number of experiments, likely due to water being present in the solvents (no absolute solvents were used) and atmosphere. The samples were measured "wet", *i.e.* in mother-liquor, and covered with a foil to avoid/slow down the **AH-I** to **Hy1-I** transformation due exposure to RH.

Table S8. SGD slurry experiments in water and organic solvents starting from **AH-I**.

Solvent (mL)	Solid-state form[a]		
	after 1 day	after 7 days	after 14 days
methanol (0.2)	**AH-I** + **Hy1-I**	**AH-I** + **Hy1-I**	**AH-I** + **Hy1-I**
ethanol (0.2)	**AH-I**	**AH-I**	**AH-I** + **Hy1-I**
n-propanol (0.3)	**AH-I**	**AH-I**	**AH-I**
i-propanol (0.3)	**AH-I**	**AH-I**	**AH-I**
n-butanol (0.3)	**AH-I** + **Hy1-I**	**AH-I** + **Hy1-I**	**AH-I** + **Hy1-I**
i-butanol (0.3)	**AH-I** + **Hy1-I**	**AH-I** + **Hy1-I**	**AH-I** + **Hy1-I**
t-butanol (0.3)	**S-*t*BuOH**	**S-*t*BuOH**	**S-*t*BuOH**
n-pentanol (0.3)	**AH-I** + **Hy1-I**	**AH-I** + **Hy1-I**	**AH-I** + **Hy1-I**
i-pentanol (0.3)	**AH-I** + **Hy1-I**	**Hy1-I**	**Hy1-I**
t-pentanol (0.3)	**AH-I** + **Hy1-I**	**AH-I** + **Hy1-I**	**AH-I** + **Hy1-I**
water (0.3)	**Hy1-I**	**Hy1-I**	**Hy1-I**
diethyl ether (0.3)	**AH-I**	**AH-I**	**AH-I**
di-*i*-propyl ether (0.3)	**AH-I**	**AH-I**	**AH-I**
methyl-*t*-butyl ether (0.3)	**AH-I**	**AH-I**	**AH-I**
tetrahydrofuran (0.3)	**S-THF**	**S-THF**	**S-THF**
1,4-dioxane (0.3)	**AH-I**	**Hy1-I**	**Hy1-I**
acetone (0.2)	**S-ACO**	**S-ACO**	**S-ACO**
cyclohexanone (0.2)	**S-CHXO**	**S-CHXO**	**S-CHXO**
methyl ethyl ketone (0.2)	**AH-I**	**Hy1-I**	**Hy1-I**
methyl *i*-butyl ketone (0.3)	**AH-I**	**AH-I** + **Hy1-I**	**AH-I** + **Hy1-I**
ethyl acetate (0.3)	**AH-I**	**Hy1-I**	**Hy1-I**
butyl acetate (0.3)	**AH-I**	**AH-I** + **Hy1-I**	**Hy1-I**
i-butyl acetate (0.3)	**AH-I**	**AH-I** + **Hy1-I**	**AH-I** + **Hy1-I**
dimethyl carbonate	**Hy1-I**	**Hy1-I**	**Hy1-I**
nitromethane (0.3)	**AH-I**	**Hy1-I**	**Hy1-I**
acetonitrile (0.2)	**AH-I**	**AH-I**	**Hy1-I**
dimethyl sulfoxide (0.1)	**S-DMSO**	**S-DMSO**	**S-DMSO**
dimethyl formamide (0.1)	**S-DMF**	**S-DMF**	**S-DMF**
dimethyl acetamide (0.1)	**S-DMA**	**S-DMA**	**S-DMA**
dichloromethane (0.3)	**AH-I**	**AH-I**	**AH-I**
chloroform (0.3)	**AH-I**	**AH-I**	**AH-I** + **Hy1-I**
n-heptane (0.3)	**AH-I**	**AH-I**	**AH-I** + **Hy1-I**
cyclohexane (0.3)	**AH-I**	**AH-I**	**AH-I**
toluene (0.3)	**AH-I**	**AH-I**	**AH-I** + **Hy1-I**

[a] AH – anhydrate, Hy – hydrate, S – solvate.

Additional slurry experiments were conducted using various SGD solid-state forms or mixtures thereof (Table S9). Interestingly, when **Hy1-I** was used as the starting material, two additional solvates were discovered, namely, ones formed with MeOH and EtOH. However, it was observed that these two solvates exhibited instability under ambient conditions. Upon removal from the mother liquor, an immediate transformation into the hydrate (**Hy1-I**) was observed.

Furthermore, when **AH-II** or a 1:1 mixture of **AH-I** and **AH-II** was used as the starting point, both of these anhydrates, when used in their pure phase, did not transform to another polymorph. In the case of the **AH-I**:**AH-II** mixture, a very gradual shift towards **AH-II** was observed, although it only became noticeable after two weeks.

Table S9. SGD slurry experiments in organic solvents starting from **Hy1-I**, **AH-II** or **AH-I** & **AH-II** mixture.

Solvent (mL)	Starting material[a]	Solid-state form[a]		
		after 1 day	after 7 days	after 14 days
methanol (0.2)	**Hy1-I**	**S-MeOH**	**S-MeOH**	**S-MeOH**
ethanol (0.2)	**Hy1-I**	**Hy1-I**	**S-EtOH** + **Hy1**	**S-EtOH** + **Hy1**
n-propanol (0.3)	**Hy1-I**	**Hy1-I** + **AH I**	**Hy1-I** + **AH-I**	**Hy1-I** + **AH-I**
i-propanol (0.3)	**Hy1-I**	**Hy1-I** + **AH I**	**Hy1-I** + **AH-I**	**Hy1-I** + **AH-I**
n-butanol (0.3)	**Hy1-I**	**Hy1-I** + **AH I**	**Hy1-I** + **AH-I**	**Hy1-I** + **AH-I**
i-butanol (0.3)	**Hy1-I**	**Hy1-I** + **AH I**	**Hy1-I** + **AH-I**	**Hy1-I** + **AH-I**
t-butanol (0.3)	**Hy1-I**	**S-*t*BuOH**	**S-*t*BuOH**	**S-*t*BuOH**
n-pentanol (0.3)	**Hy1-I**	**Hy1-I** + **AH I**	**Hy1-I** + **AH-I**	**Hy1-I** + **AH-I**
i-pentanol (0.3)	**Hy1-I**	**Hy1-I**	**Hy1-I**	**Hy1-I**
t-pentanol (0.3)	**Hy1-I**	**Hy1-I** + **AH I**	**Hy1-I** + **AH-I**	**Hy1-I** + **AH-I**
n-propanol (0.3)	**AH-II**	**AH-II**	**AH-II**	**AH-II**
n-propanol (0.3)	**AH-I** & **AH-II**	**AH-I** & **AH-II**	**AH-I** & **AH-II**	**AH-I** < **AH-II**
methyl-*t*-butyl ether	**AH-II**	**AH-II**	**AH-II**	**AH-II**
methyl-*t*-butyl ether	**AH-I** & **AH-II**	**AH-I** & **AH-II**	**AH-I** & **AH-II**	**AH-I** < **AH-II**
n-heptane (0.3)	**AH-II**	**AH-II**	**AH-II**	**AH-II**
n-heptane (0.3)	**AH-I** & **AH-II**	**AH-I** & **AH-II**	**AH-I** & **AH-II**	**AH-I** < **AH-II**

[a] AH – anhydrate, Hy – hydrate, S – solvate.

3.4. Solvent crystallization experiments

The temperature-dependent solubility of SGD **AH-I** was determined in 29 different solvents using the Crystal16® crystallization system. The amount of substance used ranged from 1 mg to 40 mg, and the volume of the solvent used ranged from 1 mL to 1.4 mL. The samples were then subjected to a predetermined temperature program, applying heating and cooling rates of 0.2 °C min^{-1}. The upper limit was set to approximately 10 °C below the boiling point of each solvent.

Out of the 29 solvents investigated, temperature-dependent solubility curves were obtained for 12 solvents. The measured "clear points" indicate the temperature at which the compound completely dissolves. On the other hand, the "cloud points" indicate the temperature at which the substance crystallizes (Figure S9 & S10). The products obtained after the solubility experiments were then analyzed using PXRD and corresponded predominantly to **Hy1-I**. Due to the sample preparation, specifically filtration and grinding, transformation to **Hy1-I** may have occurred during these processes.

For following solvents, the solubility was too low (<1-2 mg mL^{-1} close to the boiling point of the solvent): *i*-butanol, *n*-pentanol, tetrahydrofuran, methyl ethyl ketone, methyl *i*-butyl ketone, dimethyl carbonate, ethyl acetate, butyl acetate, *i*-butyl acetate, diethyl ether, methyl *t*-butyl ether, di-*i*-propyl ether, dichloromethane, chloroform, toluene, cyclohexane, and *n*-heptane.

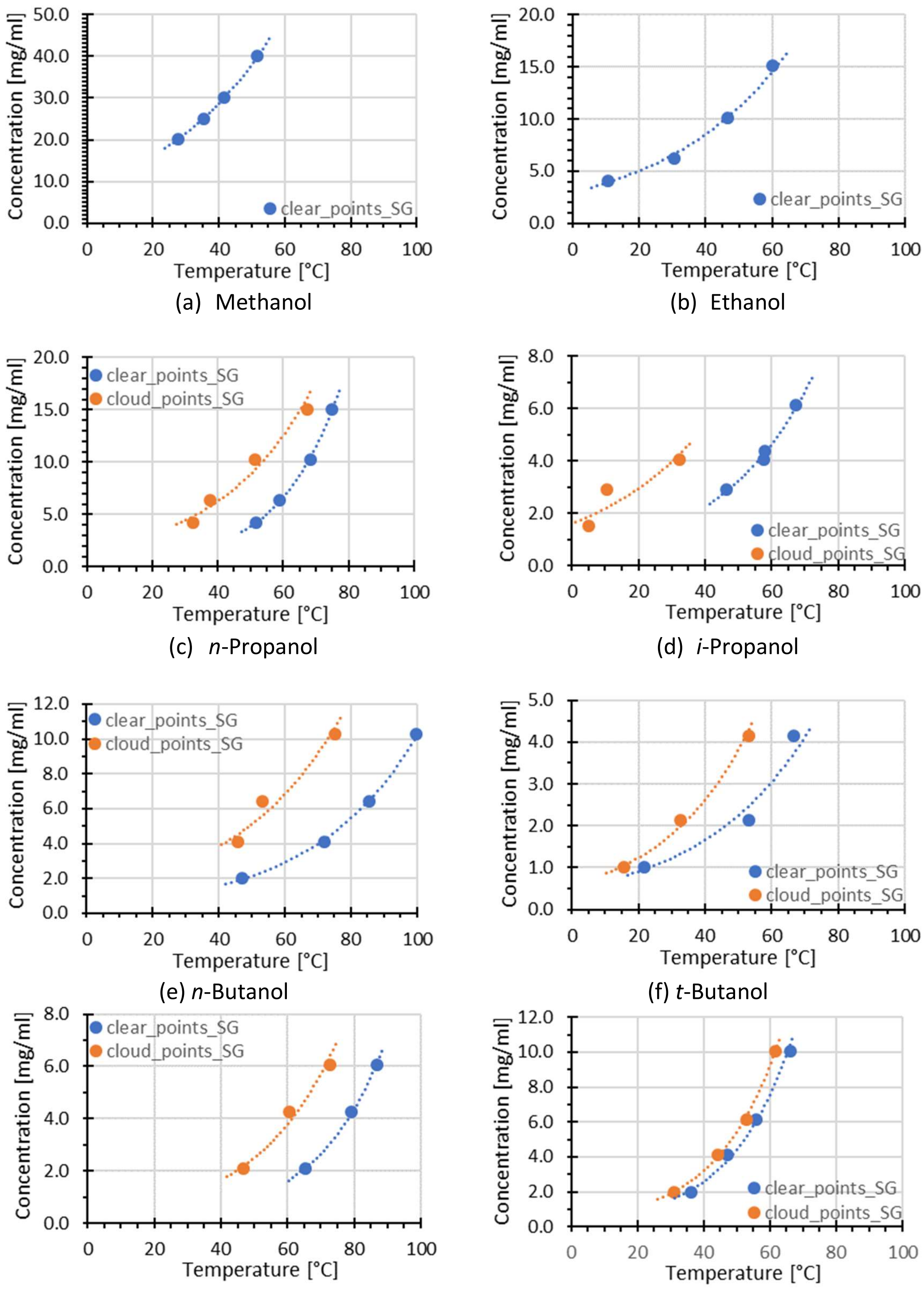

(a) Methanol (b) Ethanol

(c) *n*-Propanol (d) *i*-Propanol

(e) *n*-Butanol (f) *t*-Butanol

(g) *i*-Pentanol (h) Water

Figure S9. Crystal 16® solubility curves of SGD in selected solvents. Note that crystallization upon cooling occurred only for a subset of the solvents used.

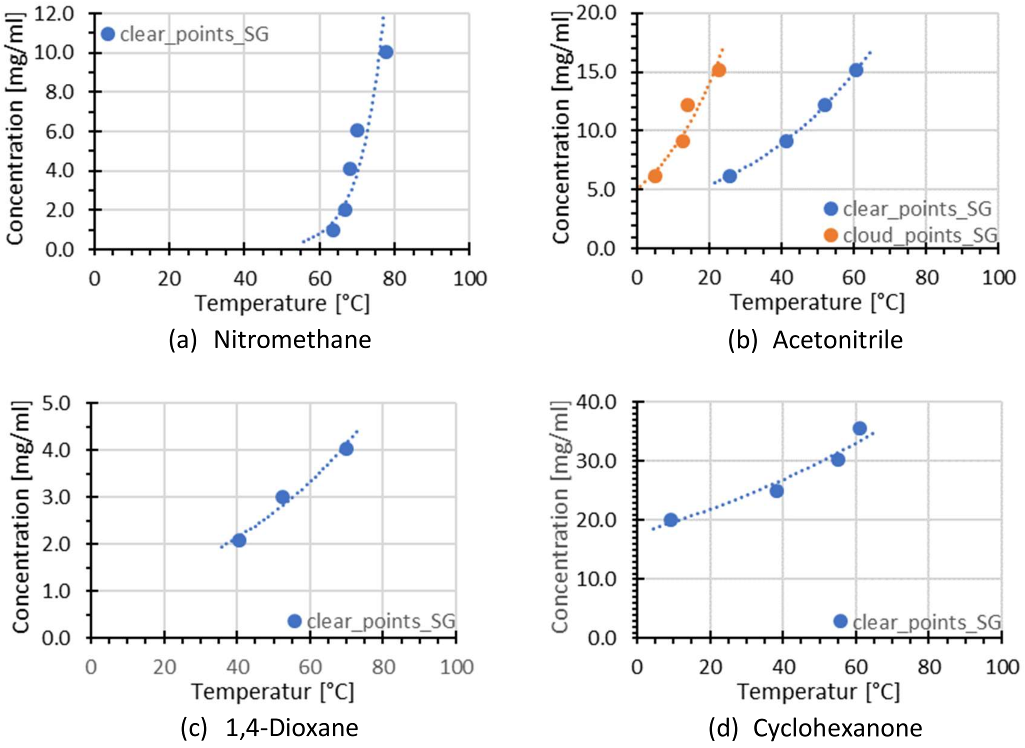

Figure S10. Crystal 16® solubility curves of SGD in selected solvents. Note that crystallization upon cooling occurred only for a subset of the solvents used.

4. Crystal structures

4.1. Structure solution from PXRD data

Table S10. Experimental details for the SGD anhydrate and solvate structures (part 1).

Crystal data	**AH-I**	**AH-V**	**S-THF**	**S-DMSO**	**S-DMA**
Chemical formula	$C_7H_{10}N_4O_2S$	$C_7H_{10}N_4O_2S$	$C_7H_{10}N_4O_2S\cdot C_4H_8O$	C7H10N4O2S· C2H6OS	$C_7H_{10}N_4O_2S\cdot C_4H_9NO$
Molar mass	214.25	214.25	286.35	292.38	301.37
Crystal system	Triclinic	Monoclinic	Orthorhombic	Orthorhombic	Orthorhombic
Space group	*P*-1	$P2_1/n$	$P2_12_12_1$	$P2_12_12_1$	$P2_12_12_1$
a / Å	6.9924(<1)	10.056(<1)	9.8324(<1)	10.0367(<1)	9.6441(1)
b / Å	10.1587(<1)	10.148(<1)	11.1144(1)	10.8651(1)	10.5177(1)
c / Å	20.5888(2)	10.958(<1)	13.1626(1)	13.2347(1)	31.3681(3)
α / °	99.783(<1)	90	90	90	90
β / °	94.843(<1)	108.495(1)	90	90	90
γ / °	97.579(<1)	90	90	90	90
Volume / $Å^3$	1420.06(2)	1060.46(4)	1438.42(2)	1443.24(2)	3181.81(6)
Z	6	4	4	4	8
Radiation type	Cu $K\alpha_{1,2}$				
μ / mm^{-1}	2.918	2.61	2.11	3.43	1.95
Data collection					
Diffractometer	Panalytical X'Pert PRO				
Temp. / K	298				
Data collection mode	Transmission				
2θ values / °	$2\theta_{min}$ = 2.001<br>$2\theta_{max}$ = 69.985 (**AH-V**: 39.985[a])<br>$2\theta_{step}$ = 0.007 (**AH-I**), 0.013				
Refinement					
Parameters	72<br>Profile: 20<br>Cell: 6<br>Scale: 1<br>U_{iso}: 1<br>Preferred orientation: 26<br>Positions: 18	38<br>Profile: 20<br>Cell: 4<br>Scale: 1<br>U_{iso}: 1<br>Preferred orientation: 6<br>Positions: 6	47<br>Profile: 20<br>Cell: 3<br>Scale: 1<br>U_{iso}: 1<br>Preferred orientation: 10<br>Positions: 12	43<br>Profile: 24<br>Cell: 3<br>Scale: 1<br>U_{iso}: 1<br>Preferred orientation: 2<br>Positions: 12	58<br>Profile: 28<br>Cell: 3<br>Scale: 1<br>U_{iso}: 1<br>Preferred orientation: 1<br>Positions: 24
R_p	2.65	2.37	3.40	3.13	3.59
R_{wp}	3.57	3.07	4.48	5.25	5.69
R_{exp}	1.85	1.79	2.49	3.59	1.70
R_{Bragg}	1.99	1.97	3.07	2.77	2.98
χ^2	3.74	2.94	3.24	2.14	11.18

[a]measured at 0% RH using a non-ambient RH chamber.

Table S11. Experimental details for the SGD solvate structures (part 2).

Crystal data	**S-DMF**	**S-MeOH**	**S-*t*BuOH**	**S-ACO**
Chemical formula	$C_7H_{10}N_4O_2S{\cdot}C_3H_7NO$	$C_7H_{10}N_4O_2S{\cdot}C_1H_4O$	$C_7H_{10}N_4O_2S{\cdot}C_4H_{10}O$	$2(C_7H_{10}N_4O_2S){\cdot}C_3H_6O$
Molar mass	287.35	246.29	288.37	486.58
Crystal system	Monoclinic	Orthorhombic	Monoclinic	Orthorhombic
Space group	$P2_1$	*Pbca*	$P2_1/c$	*Pbca*
a / Å	7.0231 (<1)	12.0197(2)	17.7340(2)	12.6609(1)
b / Å	12.1415(2)	6.9858(1)	6.8737(<1)	18.7414(1)
c / Å	8.2156(1)	27.2785(4)	12.2851(1)	19.2784(2)
α / °	90	90	90	90
β / °	94.249(1)	90	93.732(<1)	90
γ / °	90	90	90	90
Volume / $Å^3$	698.63(2)	2290.50(6)	1494.37(2)	4574.46(7)
Z	2	8	4	8
Radiation type	Cu $K\alpha_{1,2}$			
μ / mm^{-1}	2.19	2.551	2.03	2.52
Data collection				
Diffractometer	Panalytical X'Pert PRO			
Temp. / K	298			
Data collection mode	Transmission			
2θ values / °	$2\theta_{min}$ = 2.001<br>$2\theta_{max}$ = 69.985<br>$2\theta_{step}$ = 0.013			
Refinement				
Parameters	38<br>Profile: 20<br>Cell: 4<br>Scale: 1<br>U_{iso}: 1<br>Preferred orientation: 0<br>Positions: 12	54<br>Profile: 20<br>Cell: 3<br>Scale: 1<br>U_{iso}: 1<br>Preferred orientation: 17<br>Positions: 12	55<br>Profile: 20<br>Cell: 4<br>Scale: 1<br>U_{iso}: 1<br>Preferred orientation: 17<br>Positions: 12	57<br>Profile: 24<br>Cell: 3<br>Scale: 1<br>U_{iso}: 1<br>Preferred orientation: 10<br>Positions: 18
R_p	2.75	3.02	2.83	3.39
R_{wp}	3.90	4.19	4.05	4.66
R_{exp}	2.07	2.52	2.35	2.64
R_{Bragg}	2.17	2.26	1.69	2.37
χ^2	3.54	2.76	2.99	3.12

4.1.1. Anhydrates

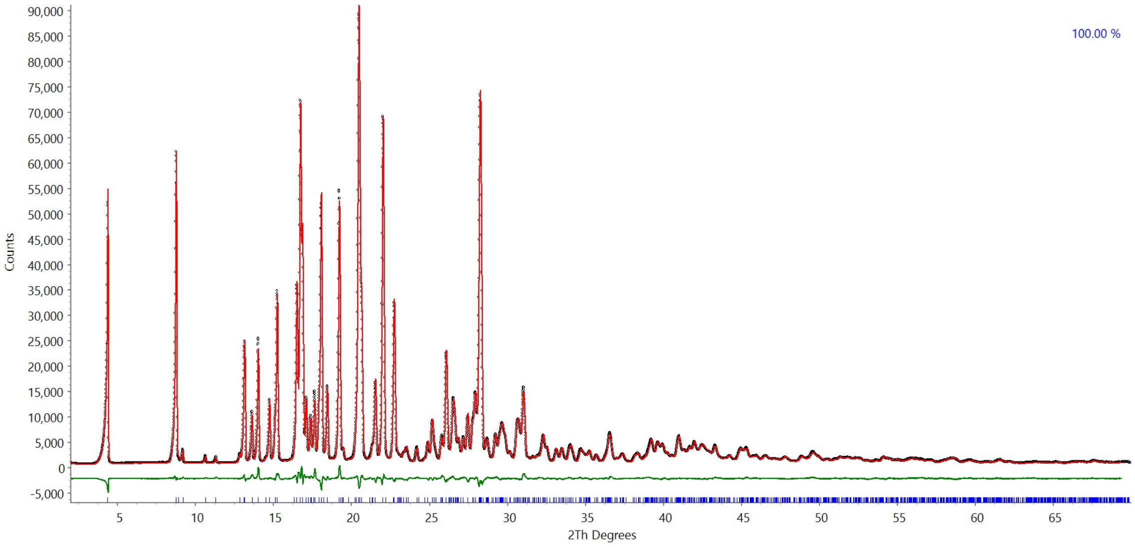

Figure S11. Powder X-ray diffraction pattern and Rietveld fit (rigid body) of **AH-I**: Observed (black points), calculated (red line), and difference profiles (green line). Blue tick marks denote the peak positions.

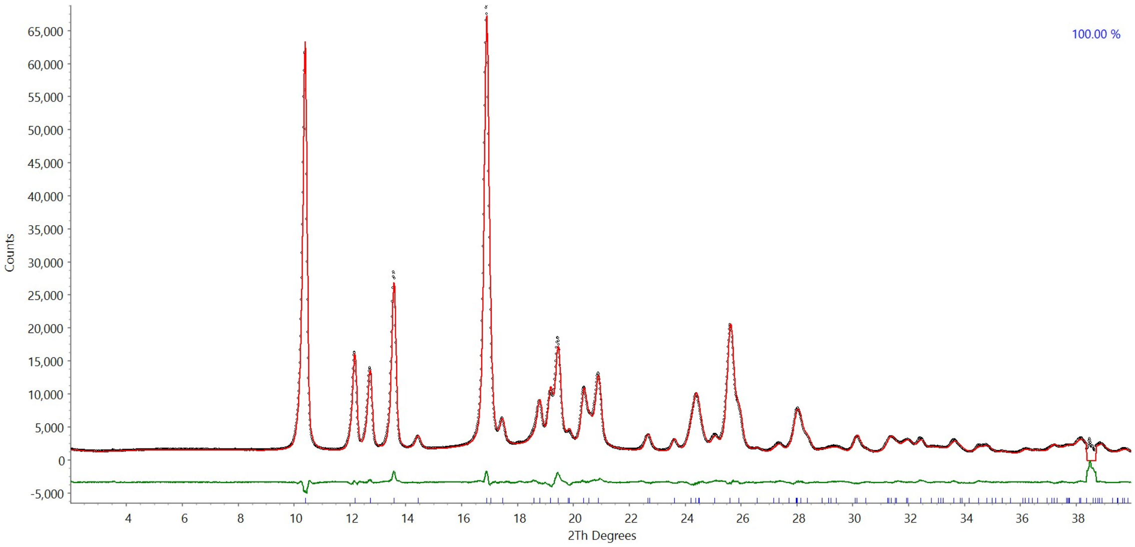

Figure S12. Powder X-ray diffraction pattern and Rietveld fit (rigid body) of **AH-V**: Observed (black points), calculated (red line), and difference profiles (green line). Blue tick marks denote the peak positions. The sample was measured on Al-foil.

4.1.2. Solvates

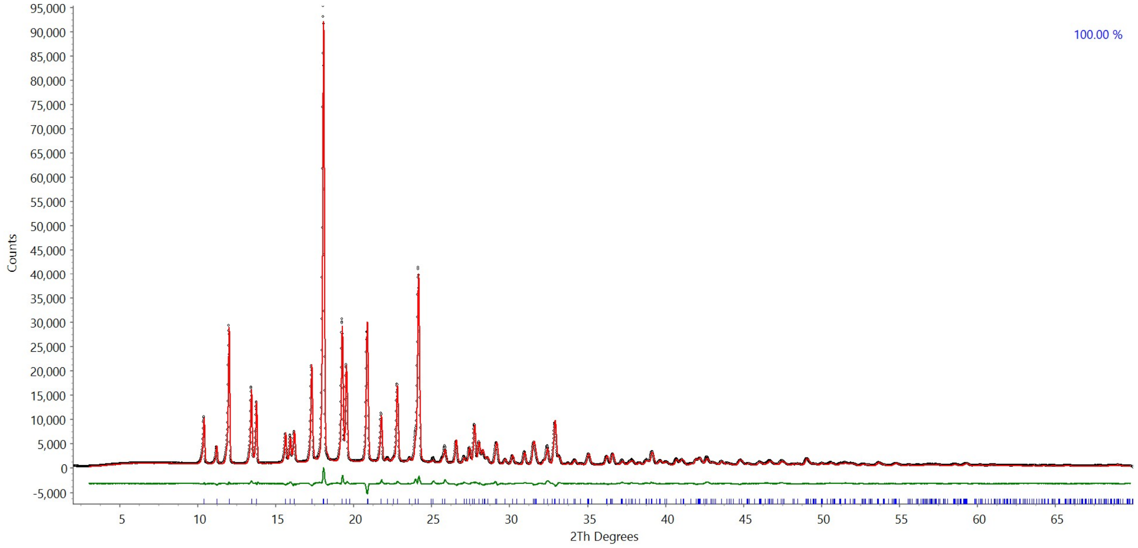

Figure S13. Powder X-ray diffraction pattern and Rietveld fit (rigid body) of **S-THF**: Observed (black points), calculated (red line), and difference profiles (green line). Blue tick marks denote the peak positions.

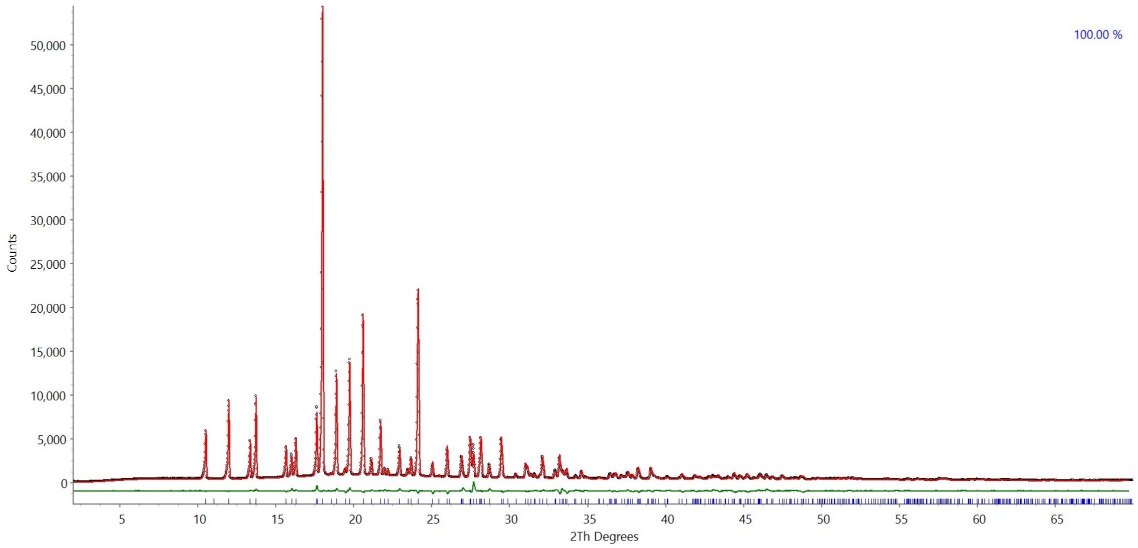

Figure S14. Powder X-ray diffraction pattern and Rietveld fit (rigid body) of **S-DMSO**: Observed (black points), calculated (red line), and difference profiles (green line). Blue tick marks denote the peak positions.

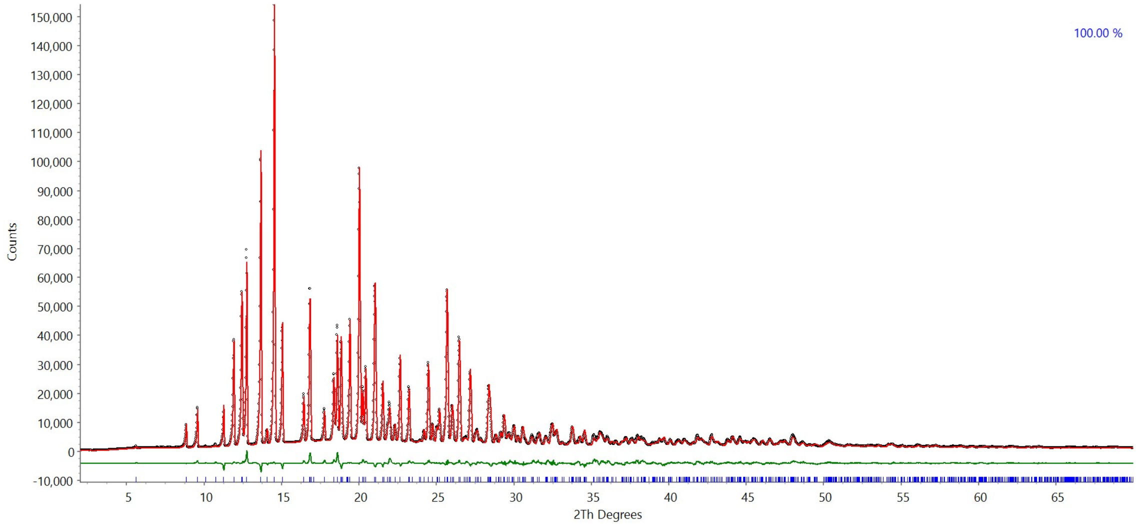

Figure S15. Powder X-ray diffraction pattern and Rietveld fit (rigid body) of **S-DMA**: Observed (black points), calculated (red line), and difference profiles (green line). Blue tick marks denote the peak positions.

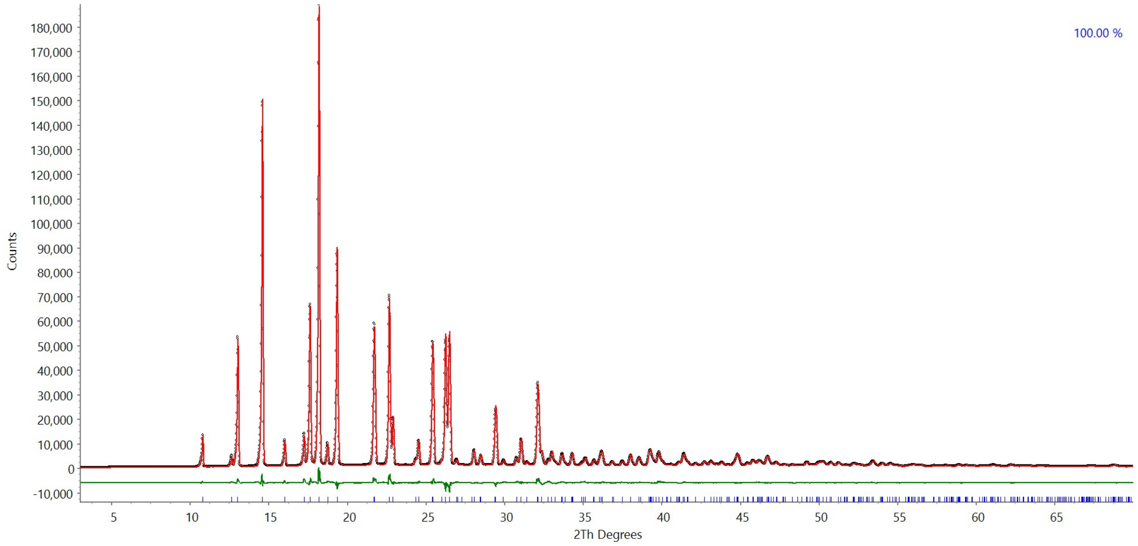

Figure S16. Powder X-ray diffraction pattern and Rietveld fit (rigid body) of **S-DMF**: Observed (black points), calculated (red line), and difference profiles (green line). Blue tick marks denote the peak positions.

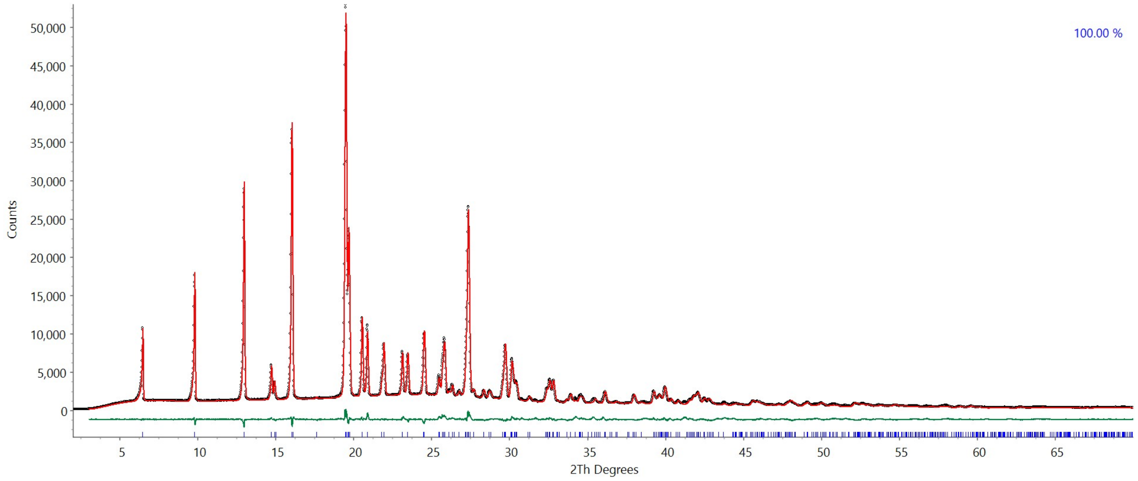

Figure S17. Powder X-ray diffraction pattern and Rietveld fit (rigid body) of **S-MeOH**: Observed (black points), calculated (red line), and difference profiles (green line). Blue tick marks denote the peak positions.

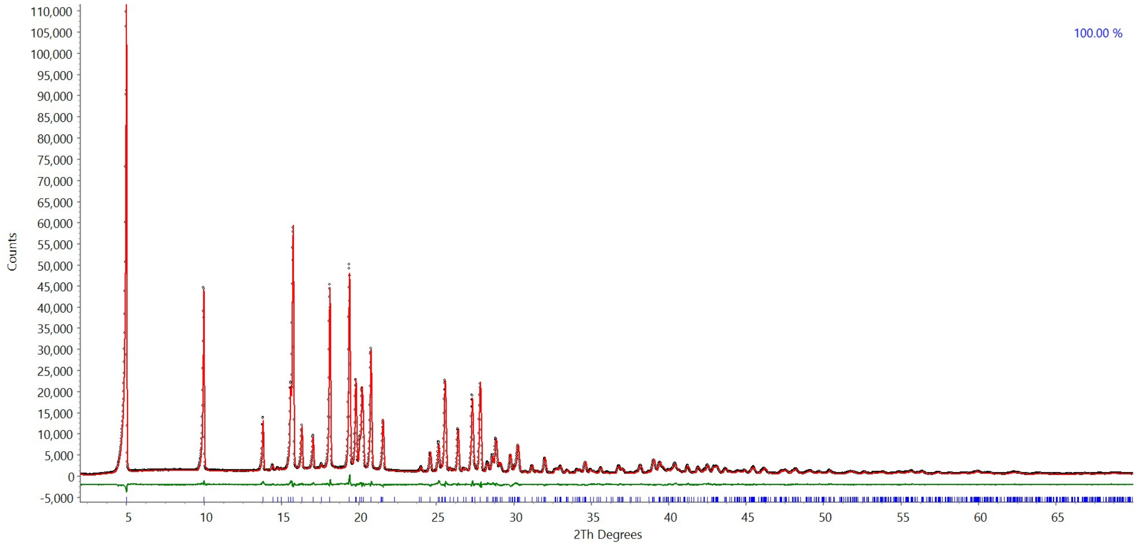

Figure S18. Powder X-ray diffraction pattern and Rietveld fit (rigid body) of **S-*t*BuOH**: Observed (black points), calculated (red line), and difference profiles (green line). Blue tick marks denote the peak positions.

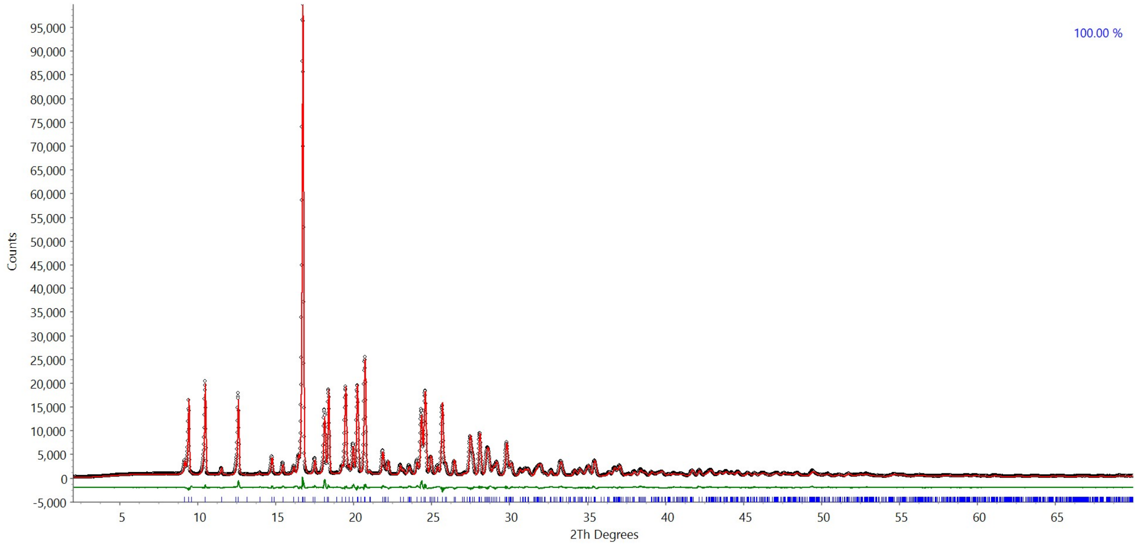

Figure S19. Powder X-ray diffraction pattern and Rietveld fit (rigid body) of **S-ACO**: Observed (black points), calculated (red line), and difference profiles (green line). Blue tick marks denote the peak positions.

4.2. Pairwise intermolecular interaction-energy calculations

4.2.1. Tetrahydrofuran (THF) solvate

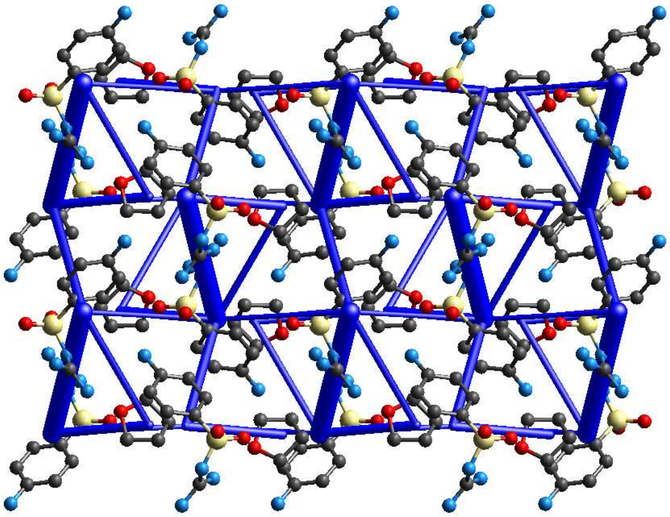

Figure S20. Energy framework diagram (total energy) for **S-THF**. The energy scale factor is 60. Stabilizing contacts are shown in blue and the thickness corresponds to the strength. Pairwise interaction energies <20 kJ mol^{-1} are omitted.

Table S12. Pairwise intermolecular interactions[a] seen in **S-THF**. SGD – sulfaguanidine, S – solvent.

No.	Interaction	Symop.	*n*	R Å	E_E	E_P	E_R kJ mol^{-1}	E_D	E_{tot}
1	SGD···SGD	x+1/2, -y+1/2, -z	2	7.4	−98.5	−28.3	−19.5	101.6	−79.3
2	SGD···SGD	x+1/2, -y+1/2, -z	2	7.28	−37.8	−12.2	−17.7	40.5	−39.3
3	SGD···S	-	2	4.92	−32.5	−8.9	−22	36.1	−37.9
4	SGD···S	-	2	6.46	−30.3	−9.8	−11.5	33.2	−28.8
5	SGD···SGD	-x+1/2, -y, z+1/2	2	6.98	−11.1	−6.6	−12.4	11.1	−20.6
6	SGD···S	-	2	4.72	−4.2	−2.3	−26.2	17	−18.5
7	SGD···SGD	-x, y+1/2, -z+1/2	2	8.24	−6.1	−3.2	−19.4	13.1	−17.6
8	SGD···S	-	2	6.7	−3.5	−3.2	−15.7	11.5	−12.6
9	SGD···S	-	2	6.13	−4.6	−3.2	−6.5	2	−11.6
10	SGD···S	-	2	6.97	−1.6	−1.6	−16.2	12.9	−9.1
11	SGD···S	-	2	6.02	0.1	−1.6	−11	4.1	−8.2
12	SGD···S	-x+1/2, -y, z+1/2	2	6.56	−0.7	−0.3	−2.6	0.5	−2.9
13	S···S	-x, y+1/2, -z+1/2	2	6.67	−0.6	−0.1	−1.8	0.2	−2.1
14	SGD···S	-	2	7.85	1	−1.5	−1.4	0.1	−1.2
15	SGD···SGD	x, y, z	2	10.99	9.9	−1.3	−2.4	0.1	7.4

[a] electrostatic (E_E), polarization (E_P), dispersion (E_D), and exchange-repulsion (E_R). $E_{tot} = k_E E_E + k_P E_P + k_D E_D + k_R E_R$, with *k* being scale factors.[12]

4.2.2. Dimethyl sulfoxide (DMSO) solvate

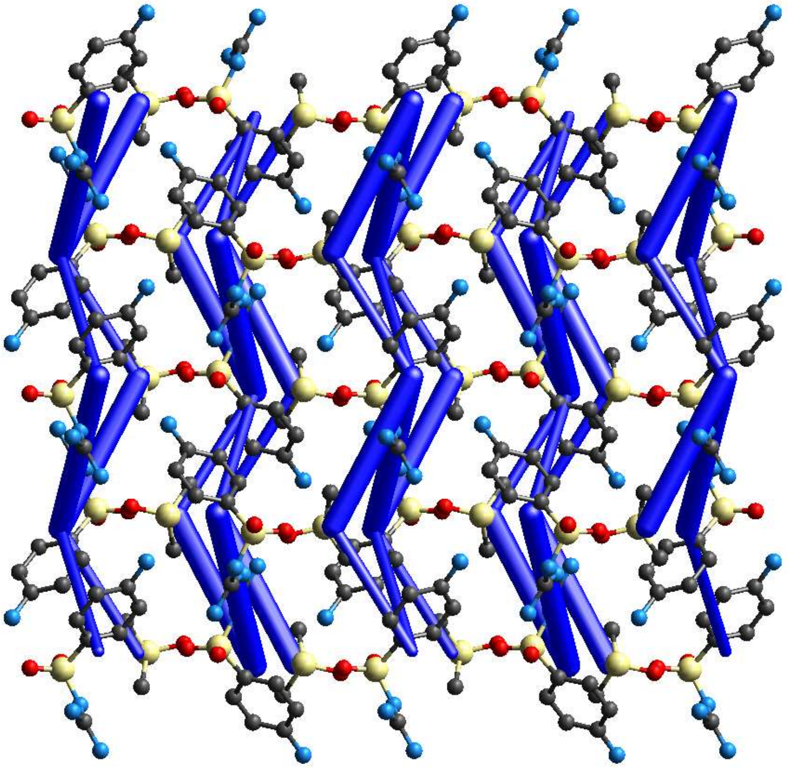

Figure S21. Energy framework diagram (total energy) for **S-DMSO**. The energy scale factor is 60. Stabilizing contacts are shown in blue and the thickness corresponds to the strength. Pairwise interaction energies <20 kJ mol^{-1} are omitted.

Table S13. Pairwise intermolecular interactions[a] seen in **S-DMSO**. SGD – sulfaguanidine, S – solvent.

No.	Interaction	Symop.	*n*	R<br>Å	E_E	E_P	E_R<br>kJ mol^{-1}	E_D	E_{tot}
1	SGD⋯SGD	x+1/2, -y+1/2, -z	2	7.61	−92.3	−26.8	−18.2	89.1	−78.2
2	SGD⋯S	-	2	4.71	−56.9	−15.7	−21.1	47.4	−60.9
3	SGD⋯SGD	x+1/2, -y+1/2, -z	2	7.25	−38.2	−12.1	−16.4	36.8	−40.9
4	SGD⋯S	-	2	6.59	−48.4	−13.3	−7.1	50.2	−36.2
5	SGD⋯SGD	-x, y+1/2, -z+1/2	2	8.03	−5.3	−3.2	−20.2	10.5	−19.1
6	SG⋯S	-	2	6.35	−9.9	−2.4	−5.1	0.7	−16.2
7	SGD⋯SGD	-x+1/2, -y, z+1/2	2	7.05	−5	−5.9	−9.7	5.3	−14.8
8	S⋯S	-x+1/2, -y, z+1/2	2	6.46	−5.9	−0.9	−1.3	0	−8
9	SGD⋯S	-	2	6.09	−2.2	−2.1	−13.4	12.2	−7.9
10	SGD⋯S	-	2	4.7	3.6	−2	−16.3	8	−6.9
11	SGD⋯S	-	2	6.84	−0.9	−2.2	−11.2	9.7	−6.3
12	SGD⋯S	-	2	6.77	−4.7	−3.8	−15.4	25.5	−5.5
13	S⋯S	-x, y+1/2, -z+1/2	2	6.62	−2	−0.3	−2.8	0.7	−4.4
14	SGD⋯SGD	x, y, z	2	11.02	11.3	−1.8	−3.5	0.4	7.8

[a] electrostatic (E_E), polarization (E_P), dispersion (E_D), and exchange-repulsion (E_R). $E_{tot} = k_E E_E + k_P E_P + k_D E_D + k_R E_R$, with *k* being scale factors.[12]

4.2.3. Dimethyl acetamide (DMA) Solvate

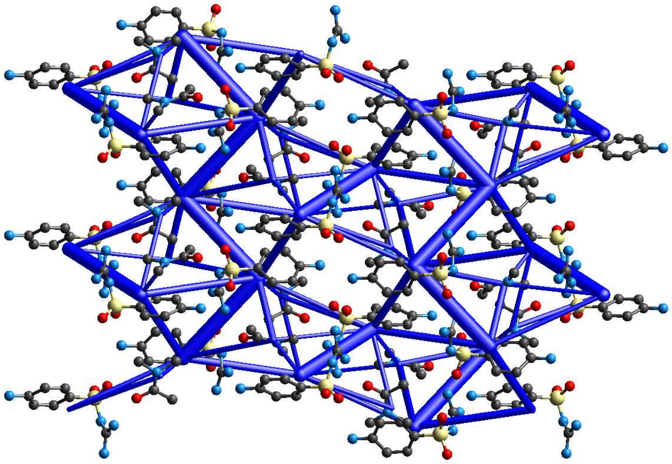

Figure S22. Energy framework diagram (total energy) for **S-DMA**. The energy scale factor is 60. Stabilizing contacts are shown in blue and the thickness corresponds to the strength. Pairwise interaction energies <20 kJ mol^{-1} are omitted.

Table S14. Pairwise intermolecular interactions[a] seen in **S-DMA**. SGD – sulfaguanidine, S – solvent.

No.	Interaction	Symop.	*n*	R<br>Å	E_E	E_P	E_R<br>kJ mol^{-1}	E_D	E_{tot}
1	SGD···SGD	-x, y+1/2, -z+1/2	2	7.03	–86.6	–24.9	–24.4	77	–83.8
2	SGD···SGD	x+1/2, -y+1/2, -z	2	7.02	–91.8	–25.4	–20.6	88	–79.5
3	SGD···SGD	-	2	7.11	–44.7	–11.2	–20	43.1	–46.3
4	SGD···SGD	-	2	8.16	–40.9	–10.1	–14.6	34.3	–42.2
5	SGD···SGD	-	2	7.04	–41.7	–12	–17.1	43	–41.3
6	SGD···S	-	2	4.82	–26.2	–7.9	–19.6	19.5	–38.5
7	SGD···S	-	2	6.84	–43.8	–13.4	–10.6	57.1	–30.2
8	SGD···S	-	2	6.44	–23.9	–6.9	–8.2	14.7	–28.5
9	SGD···SGD	-	2	8.15	–33.7	–11.6	–12.6	44.9	–27.5
10	SGD···S	-	2	5.49	–27.2	–10.8	–13.8	35.5	–26.9
11	SGD···S	-	2	4.4	–4.9	–3.3	–31.6	18.4	–23.8
12	SGD···S	-	2	4.63	–3.5	–3.5	–33.3	18.8	–23.6
13	SGD···S	-	2	6.72	–11.9	–4	–17.9	15.8	–21.3
14	SGD···S	-	2	7.23	–10.4	–4.5	–6.2	3	–17.8
15	SGD···S	-	2	5.69	–6.2	–3.1	–23.3	23.3	–14.7
16	S···S	-	2	5.08	–3.9	–1.2	–12.8	5.2	–13.1
17	SGD···S	-	2	6.83	–7.4	–1.2	–4.5	0.4	–12.4
18	S···S	-	2	6.09	–6	–1	–7.7	5.6	–10.3
19	SGD···S	-	2	7.28	–2.4	–4.7	–6.4	6.3	–7.7
20	SGD···S	-	2	7.69	–4.6	–0.7	–2.4	0.1	–7.5
21	SGD···S	-	2	9.15	0.8	–1.5	–7.1	6.2	–2.6
22	SGD···SGD	x+1/2, -y+1/2, -z	2	12.12	0.8	–0.4	–1.2	0	–0.5
23	SGD···S	-	2	9.21	3.1	–0.8	–5.8	3.5	–0.2
24	SGD···S	-	2	6.61	5.2	–1.2	–4.8	0.7	0.9
25	SGD···SGD	-	2	10.73	8.1	–1.8	–3.8	0.8	4.5
26	SGD···SGD	-	2	10.3	11.9	–2.4	–5.9	1.9	6.8

[a] electrostatic (E_E), polarization (E_P), dispersion (E_D), and exchange-repulsion (E_R). $E_{tot} = k_E\, E_E + k_P\, E_P + k_D\, E_D + k_R\, E_R$, with *k* being scale factors.[12]

4.2.4. Dimethyl formamide (DMF) Solvate

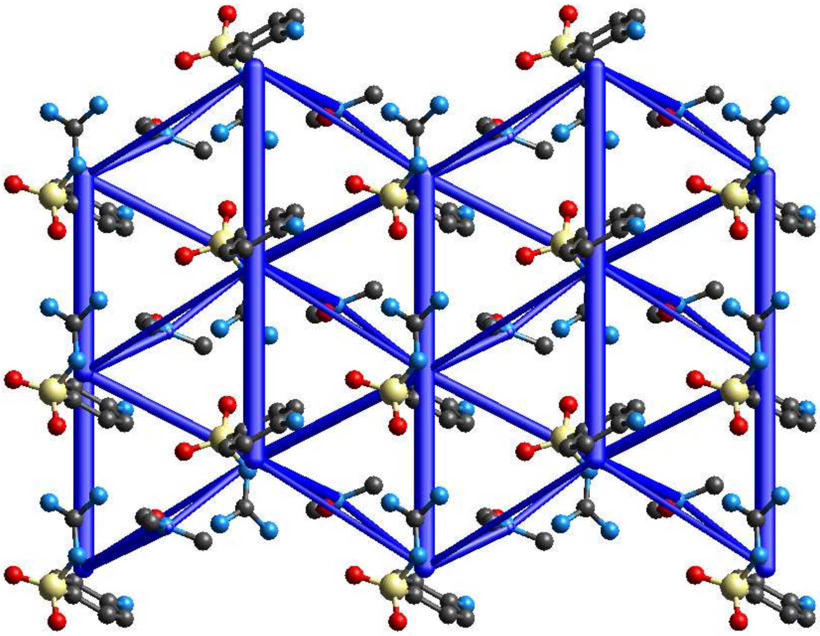

Figure S23. Energy framework diagram (total energy) for **S-DMF**. The energy scale factor is 60. Stabilizing contacts are shown in blue and the thickness corresponds to the strength. Pairwise interaction energies <20 kJ mol^{-1} are omitted.

Table S15. Pairwise intermolecular interactions[a] seen in **S-DMF**. SGD – sulfaguanidine, S – solvent.

No.	Interaction	Symop.	*n*	R Å	E_E	E_P	E_R kJ mol^{-1}	E_D	E_{tot}
1	SGD···SGD	x, y, z	2	6.95	−68.6	−18	−14.9	59	−62.4
2	SGD···SGD	-x, y+1/2, -z	2	7.91	−37.5	−8.9	−14.5	33.9	−37.8
3	SGD···S	-	2	6.96	−50.4	−13.5	−7.8	53	−37.4
4	SGD···S	-	2	4.18	−12.7	−4	−37	27.2	−31.9
5	SGD···SGD	x, y, z	2	8.01	−26.4	−13.9	−20.3	44.1	−28.7
6	SGD···S	-	2	4.79	−16	−7.3	−20.8	26.3	−24.2
7	SGD···SGD	-x, y+1/2, -z	2	8.10	−14.2	−2.8	−5.5	1	−21.3
8	SGD···S	-	2	6.26	−11.4	−2	−12.5	7.3	−19.8
9	SGD···S	-	2	7.7	−10.1	−2.7	−2.7	0.5	−14.7
10	SGD···S	-	2	6.5	−4.8	−4.5	−18.4	17	−13.9
11	S···S	-x, y+1/2, -z	2	6.15	−4	−0.5	−4	2.2	−6.8
12	SGD···SGD	-x, y+1/2, -z	2	7.49	1.6	−3.1	−3.8	0.3	−3.8
13	SGD···SGD	x, y, z	2	10.24	2.9	−3.2	−7.6	6	−2.2
14	SGD···SGD	-x, y+1/2, -z	2	8.92	2.8	−0.6	−1.9	0	0.8
15	SGD···S	-	2	5.7	9.2	−1.7	−11.4	5.7	2
16	SGD···S	-	2	10.71	3.2	−0.3	−0.8	0	2.5

[a] electrostatic (E_E), polarization (E_P), dispersion (E_D), and exchange-repulsion (E_R). $E_{tot} = k_E E_E + k_P E_P + k_D E_D + k_R E_R$, with *k* being scale factors.[12]

4.2.5. Methanol (MeOH) solvate

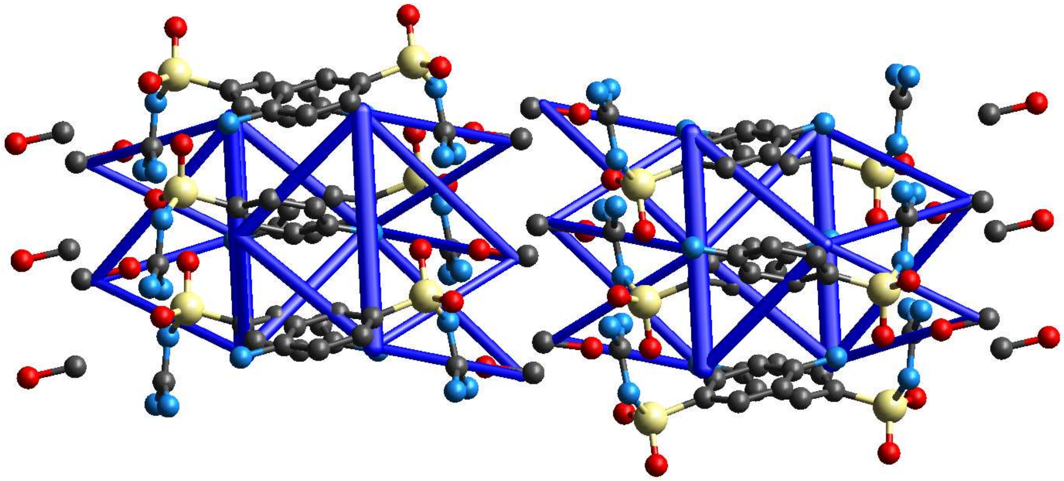

Figure S24. Energy framework diagram (total energy) for **S-MeOH**. The energy scale factor is 60. Stabilizing contacts are shown in blue and the thickness corresponds to the strength. Pairwise interaction energies <20 kJ mol^{-1} are omitted.

Table S16. Pairwise intermolecular interactions[a] seen in **S-MeOH**. SGD – sulfaguanidine, S – solvent.

No.	Interaction	Symop.	*n*	R<br>Å	E_E	E_P	E_R<br>kJ mol^{-1}	E_D	E_{tot}
1	SGD···SGD	x, y, z	2	6.98	−52.8	−12.5	−8.7	32.5	−52.6
2	SGD···SGD	-x, y+1/2, -z+1/2	2	6.22	−21.2	−11.6	−32.9	37.6	−36.4
3	SGD···S	-	2	6.69	−31.4	−7.6	−6.6	22.3	−30.9
4	SGD···SGD	-x+1/2, y+1/2, z	2	5.28	−9.9	−9.6	−37.6	32.4	−30.3
5	SGD···S	-	2	6.41	−29.5	−8.4	−8.5	31.5	−25.3
6	SGD···S	-	2	5.03	−40.7	−12	−12.8	61.1	−25.3
7	SGD···S	-	2	6.1	−8.2	−3.2	−6.5	8.5	−11.4
8	SGD···S	-	2	6.24	−4.8	−2.0	−4.5	3.2	−8.5
9	SGD···S	-	2	8.43	−1.4	−1.4	−4.2	2.6	−4.5
10	S···S	-x, -y, -z	1	4.21	−1.3	−0.2	−4.0	1.6	−4.1
11	SGD···S	-	2	6.86	0.7	−1.4	−5.7	3.8	−2.9
12	S···S	-x+1/2, y+1/2, z	2	4.16	0.1	−0.3	−3.0	1.4	−1.9
13	SGD···SGD	x+1/2, y, -z+1/2	2	7.64	8.3	−2.2	−14.2	6.0	−1.5
14	SGD···SGD	-x+1/2, y+1/2, z	2	8.77	6.0	−1.7	−2.4	0	3.0

[a] electrostatic (E_E), polarization (E_P), dispersion (E_D), and exchange-repulsion (E_R). $E_{tot} = k_E E_E + k_P E_P + k_D E_D + k_R E_R$, with *k* being scale factors.[12]

4.2.6. *t*-BuOH solvate

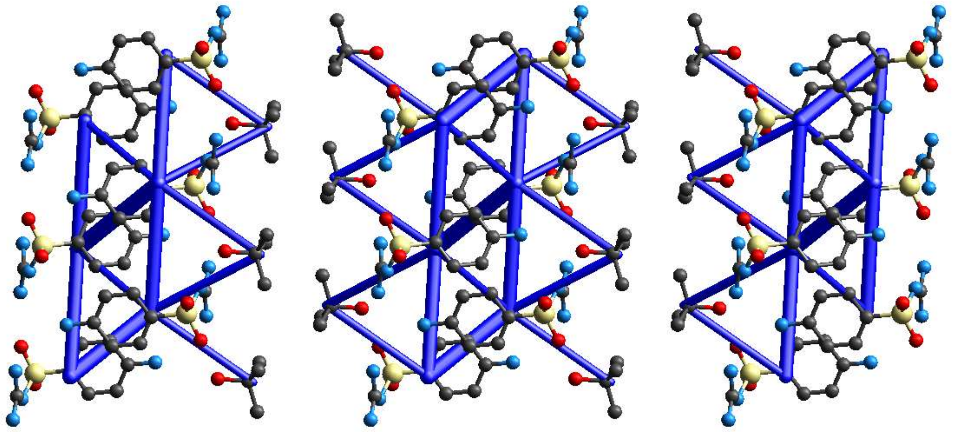

Figure S25. Energy framework diagram (total energy) for **S-*t*BuOH**. The energy scale factor is 60. Stabilizing contacts are shown in blue and the thickness corresponds to the strength. Pairwise interaction energies <20 kJ mol^{-1} are omitted.

Table S17. Pairwise intermolecular interactions[a] seen in **S-*t*BuOH**. SGD – sulfaguanidine, S – solvent.

No.	Interaction	Symop.	*n*	R<br>Å	E_E	E_P	E_R<br>kJ mol^{-1}	E_D	E_{tot}
1	SGD···SGD	-x, -y, -z	1	6.48	−38.5	−9.1	−36.7	21.7	−66
2	SGD···SGD	x, -y+1/2, z+1/2	2	6.08	−56.6	−12.1	−24.7	56.1	−55.6
3	SGD···SGD	x, y, z	2	6.76	−59	−15.3	−11.2	48.5	−53.4
4	SGD···S	-	2	7.12	−33.7	−10.4	−15.9	34.3	−36
5	SGD···S	-	2	5.7	−22.5	−6.5	−18.6	19.2	−33
6	SGD···S	-	2	5.6	−36.1	−13.5	−20.7	57.9	−30.5
7	SGD···SGD	-x, y+1/2, -z+1/2	2	6.33	−10.8	−8.3	−28.7	21.1	−29.5
8	SGD···SGD	-x, -y, -z	1	6.69	−1.7	−5.7	−39.9	36.2	−18.4
9	SGD···S	-	2	9	−10.1	−2	−5.3	3.9	−14.4
10	S···S	-x, -y, -z	1	5.41	−4	−0.1	−15.1	16.7	−7.1
11	S···S	-x, y+1/2, -z+1/2	2	5.58	−2.6	−0.1	−12.6	12.8	−6
12	S···S	x, -y+1/2, z+1/2	2	6.62	−0.5	0	−4.5	3	−2.6
13	S···S	x, y, z	2	6.76	−0.4	0	−3.3	1.3	−2.5
14	S···S	x, -y+1/2, z+1/2	2	7.33	−0.2	0	−1.4	0.1	−1.4
15	SGD···SGD	x, -y+1/2, z+1/2	2	9.24	4.3	−0.8	−1.6	0	2.5
16	SGD···S	-	2	7	8.4	−2.5	−3.1	0.3	4.5

[a] electrostatic (E_E), polarization (E_P), dispersion (E_D), and exchange-repulsion (E_R). $E_{tot} = k_E E_E + k_P E_P + k_D E_D + k_R E_R$, with *k* being scale factors.[12]

4.2.7. Acetone (ACO) solvate

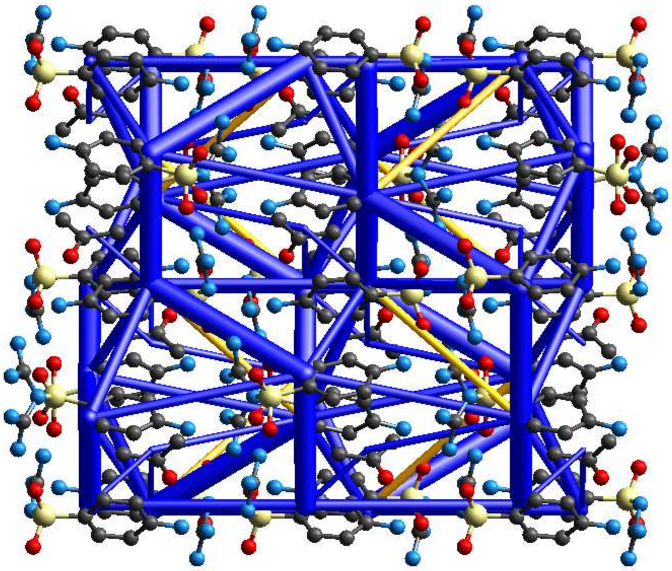

Figure S26. Energy framework diagram (total energy) for **S-ACO**. The energy scale factor is 60. Stabilizing contacts are shown in blue, destabilizing contacts in yellow and the thickness corresponds to the strength. Pairwise interaction energies <20 kJ mol^{-1} are omitted.

Table S18. Pairwise intermolecular interactions[a] seen in **S-ACO**. SGD – sulfaguanidine, S – solvent.

No.	Interaction	Symop.	*n*	R<br>Å	E_E	E_P	E_R<br>kJ mol^{-1}	E_D	E_{tot}
1	SGD···SGD	-	2	7.66	−91.8	−27.6	−19.1	83.5	−82.6
2	SGD···SGD	-	2	6.32	−87.9	−25.6	−20	81.3	−79.1
3	SGD···SGD	-	2	5.19	−56.8	−13.3	−25.3	36.1	−69.7
4	SGD···SGD	x+1/2, y, -z+1/2	2	6.39	−40.5	−10.4	−15	26.1	−47.3
5	SGD···SGD	-	2	6.98	−37.9	−9.4	−20	28.4	−46.8
6	SGD···SGD	-x+1/2, y+1/2, z	2	9.24	−35.9	−8.4	−14.6	29.9	−38.4
7	SGD···SGD	-	2	6.4	−29.2	−12.9	−35.8	54.5	−37.9
8	SGD···SGD	-x, y+1/2, -z+1/2	2	9.33	−28.8	−6.5	−17	22.2	−36.4
9	SGD···SGD	x+1/2, -y+1/2, -z	2	8.36	−26.3	−6.9	−9.5	11	−34.4
10	SGD···SGD	-x, -y, -z	1	7.48	−20.4	−2.8	−11.7	4.5	−31.1
11	SGD···S	-	2	6.38	−18.7	−4.2	−10.5	7.5	−27.4
12	SGD···S	-	2	4.38	−5.5	−3.1	−29	19.5	−21.4
13	SGD···S	-	2	4.85	−21.5	−7.2	−15.9	34.4	−20.8
14	SGD···S	-	2	4.9	−6.7	−2.7	−20	13.4	−18.2
15	SGD···S	-	2	6.08	−6.6	−1.4	−13.1	12.8	−11.5
16	S···S	-x, -y, -z	1	5.94	−3.4	−0.5	−1.7	0.2	−5.4
17	SGD···S	-	2	8.47	0.7	−0.8	−3.2	0.8	−2.1
18	SGD···SGD	-x, -y, -z	1	7.08	16.5	−4.6	−14.9	5.1	4.2
19	SGD···S	-	2	6.48	11.5	−2.6	−6.8	3	6.1
20	SGD···SGD	-x+1/2, y+1/2, z	2	11.38	9.7	−1.2	−2.3	0.2	7.4
21	SGD···SGD	-	2	10.42	13.7	−2.4	−7.1	2.5	8
22	SGD···SGD	-	2	10.36	9.5	−1	−1.2	0	8.2
23	SGD···SGD	-	2	8.33	36.1	−4.1	−2.9	0.8	33.1

[a] electrostatic (E_E), polarization (E_P), dispersion (E_D), and exchange-repulsion (E_R). $E_{tot} = k_E E_E + k_P E_P + k_D E_D + k_R E_R$, with *k* being scale factors.[12]

4.2.8. Monohydrate I (Hy1-I)

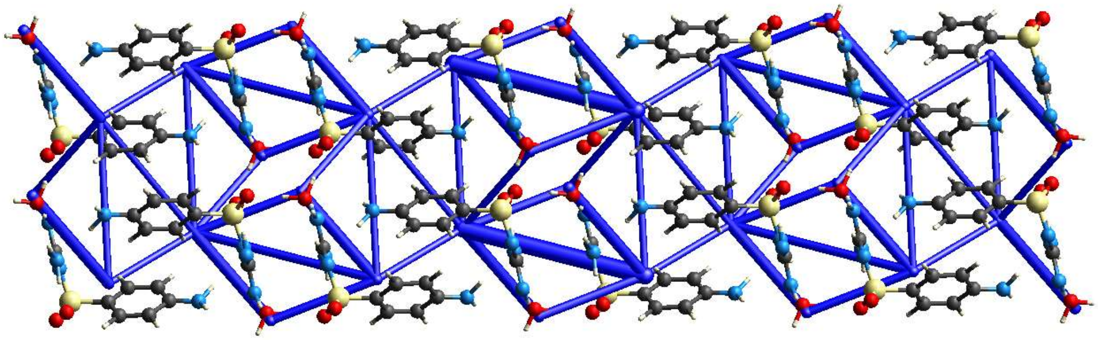

Figure S27. Energy framework diagram (total energy) for **Hy1-I**. The energy scale factor is 60. Stabilizing contacts are shown in blue and the thickness corresponds to the strength. Pairwise interaction energies <20 kJ mol^{-1} are omitted.

Table S19. Pairwise intermolecular interactions[a] seen in **Hy1-I**. SGD – sulfaguanidine, S – solvent.

No.	Interaction	Symop.	*n*	R Å	E_E	E_P	E_R kJ mol^{-1}	E_D	E_{tot}
1	SGD···SGD	-x, -y, -z	1	7.94	−48.5	−8.1	−14.8	4.8	−67.2
2	SGD···W	-	2	5.72	−53.2	−12.3	−7.3	44.1	−44.5
3	SGD···W	-	2	4.83	−53.4	−11	−7	58.4	−34.6
4	SGD···SGD	-x, -y, -z	1	8.84	−23	−4.7	−8.7	1.6	−34.4
5	SGD···SGD	x, y, z	2	5.44	−20	−18.3	−31.8	47.3	−33.1
6	SGD···W	-	2	4.91	−48.2	−10.2	−5.5	55.2	−29.2
7	SGD···SGD	x, y, z	2	7.47	−19.2	−5	−8.4	5.7	−27.8
8	SGD···SGD	-x, y+1/2, -z+1/2	2	6.21	−8.2	−6.4	−36.3	28.4	−27.5
9	SGD···SGD	-x, y+1/2, -z+1/2	2	7.44	−14.1	−4.2	−24	20.3	−26.3
10	SGD···SGD	x, y, z	2	9.24	−17.2	−1.5	−1.3	0	−20.4
11	SGD···W	-	2	5.48	−20.4	−8.5	−6.3	31.2	−14.1
12	SGD···W	-	2	7.72	−10.2	−1.3	−3.8	3	−13.2
13	W···W	-x, -y, -z	1	3.52	−3.3	−0.5	−1.9	0.8	−5.1
14	SGD···W	-	2	6.37	−3.1	−1	−1.1	0.1	−4.9
15	SGD···W	-	2	5.19	6.9	−1.5	−3.1	1.2	4.2
16	SGD···W	-	2	7.95	7	−0.9	−1.4	0.2	5.6

[a] electrostatic (E_E), polarization (E_P), dispersion (E_D), and exchange-repulsion (E_R). $E_{tot} = k_E E_E + k_P E_P + k_D E_D + k_R E_R$, with *k* being scale factors.[12]

4.3. Experimental Sulfaguanidine conformations

The experimentally observed SGD conformations have been compared to the gas phase potential energy surface (PES) scan [PBE0/6-31G(d,p)]. All conformations were identified within an energy range of 24 kJ mol^{-1} relative to the global minimum conformation, and none deviated by less than 8 kJ mol^{-1}. Disregarding the positions of the polar aniline protons, Figure S28 unveils intriguing features; specifically, the ϕ2 dihedral can vary between 5 and 355°, while the ϕ1 values fall within the ranges of 78 ± 15° and 282 ± 15°. This suggests that the formation of strong intermolecular interactions between SGD molecules and SGD + solvent molecules results in preferences for SGD conformations in the crystalline solid state. Additionally, the gas phase minimum conformation is not observed in the experimental structures. The considerable energy barriers (ΔE_{intra}) are compensated by the formation of strong intermolecular interactions in the experimental structures.

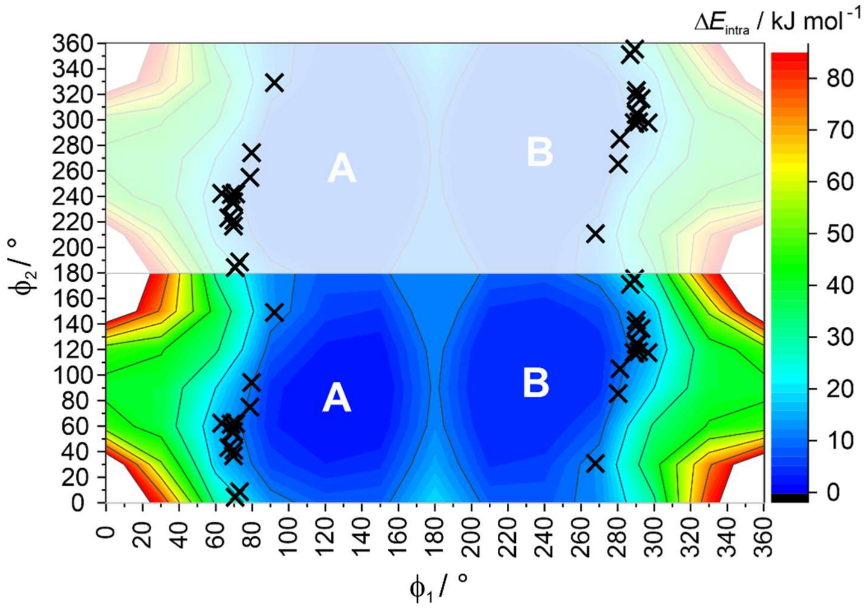

Figure S28. Experimental SGD conformations mapped onto the SGD PES.

5. Characterization of the solid-state forms

5.1. Powder X-ray diffraction

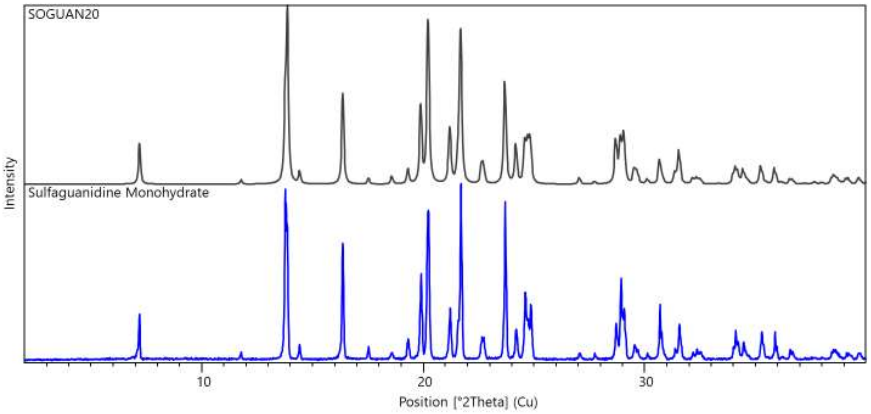

Figure S29. Comparison of the experimental SGD hydrate (**Hy1-I**) PXRD pattern to that from the single crystal structure (SOGUAN20[10]) simulated data.

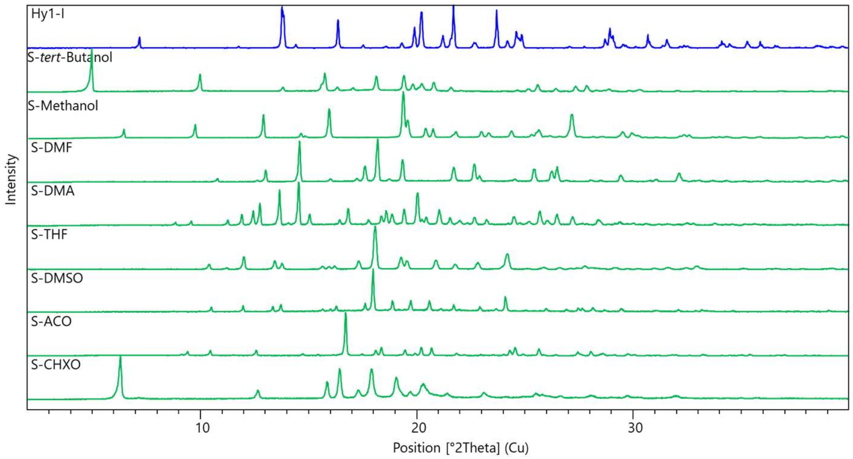

Figure S30. PXRD data comparison of the SGD **solvates** and the monohydrate **Hy1-I**.

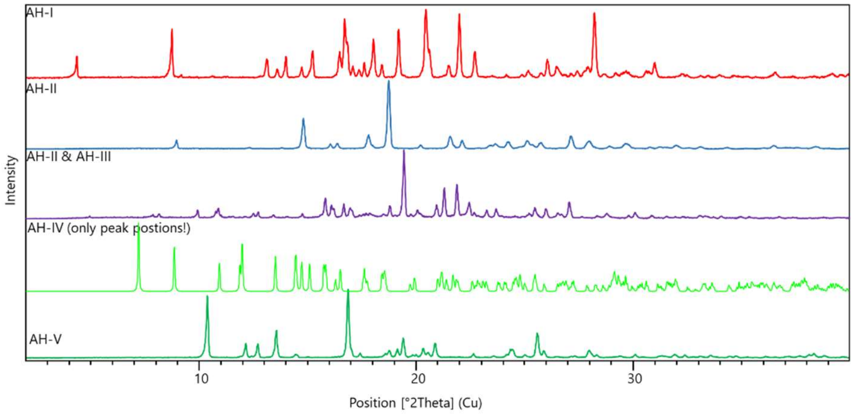

Figure S31. Comparison of the SGD **anhydrate** PXRD patterns. Note that for **AH-IV** only lattice parameters are reported and the PXRD was simulated from cell parameters only.

5.2. Temperature dependent IR spectroscopy

5.2.1. Tetrahydrofuran (THF) solvate

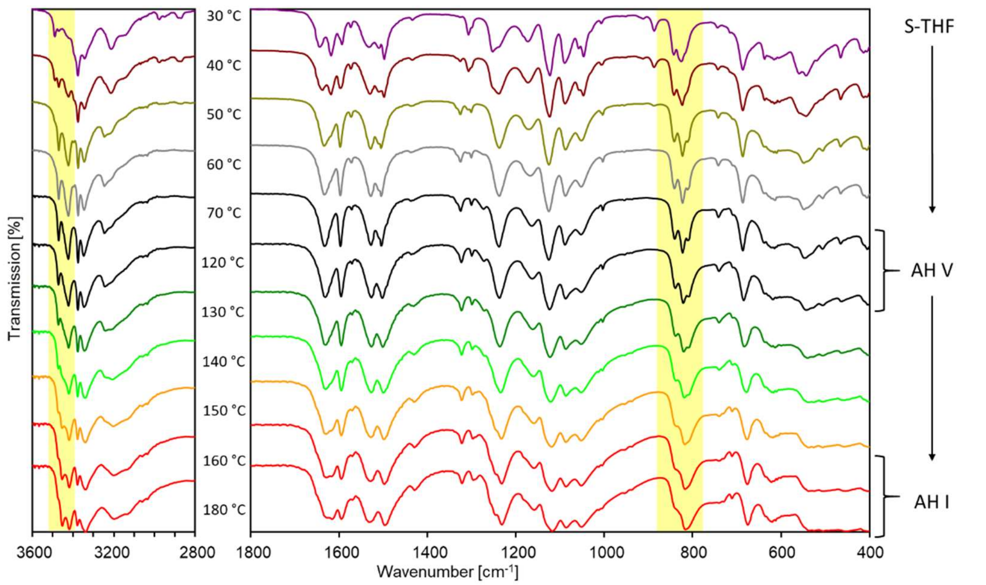

Figure S32. Temperature dependent IR spectroscopy of SGD **S-THF**. Distinctive regions are highlighted.

5.2.2. Dimethyl sulfoxide (DMSO) solvate

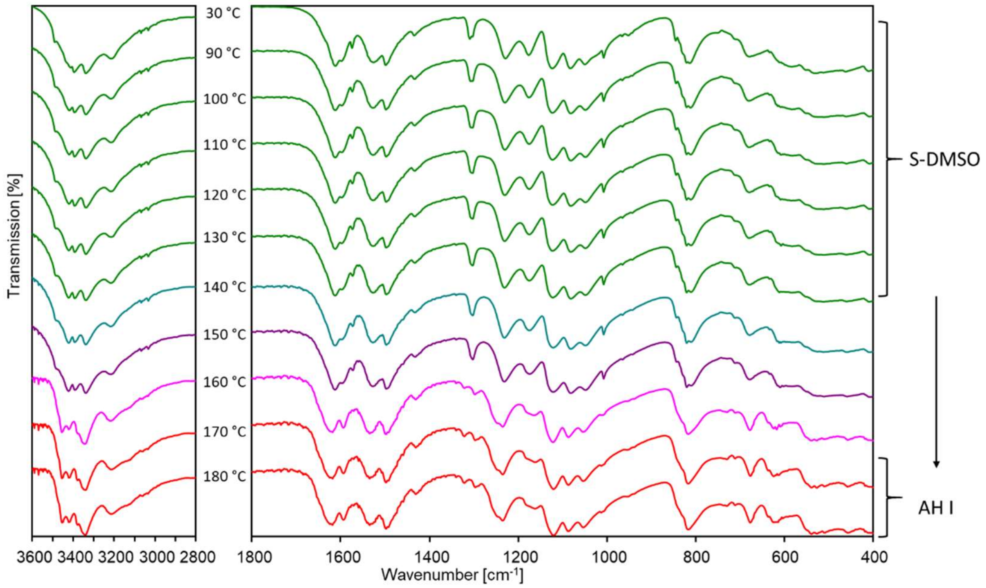

Figure S33. Temperature dependent IR spectroscopy of SGD **S-DMSO**.

5.2.3. Dimethyl acetamide (DMA) solvate

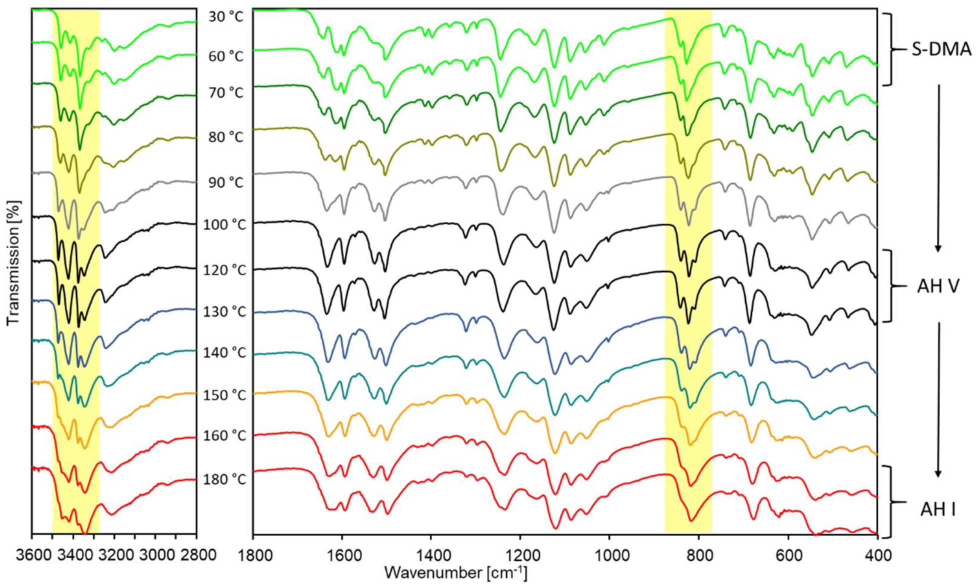

Figure S34. Temperature dependent IR spectroscopy of SGD **S-DMA**. Distinctive regions are highlighted.

5.2.4. Dimethyl formamide (DMF) solvate

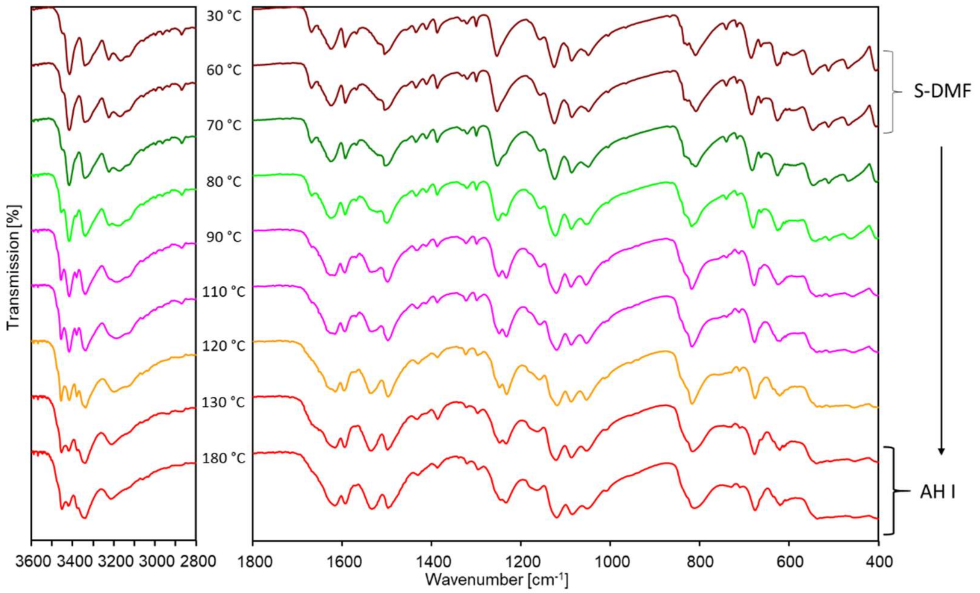

Figure S35. Temperature dependent IR spectroscopy of SGD **S-DMF**.

5.2.5. *t*-BuOH solvate

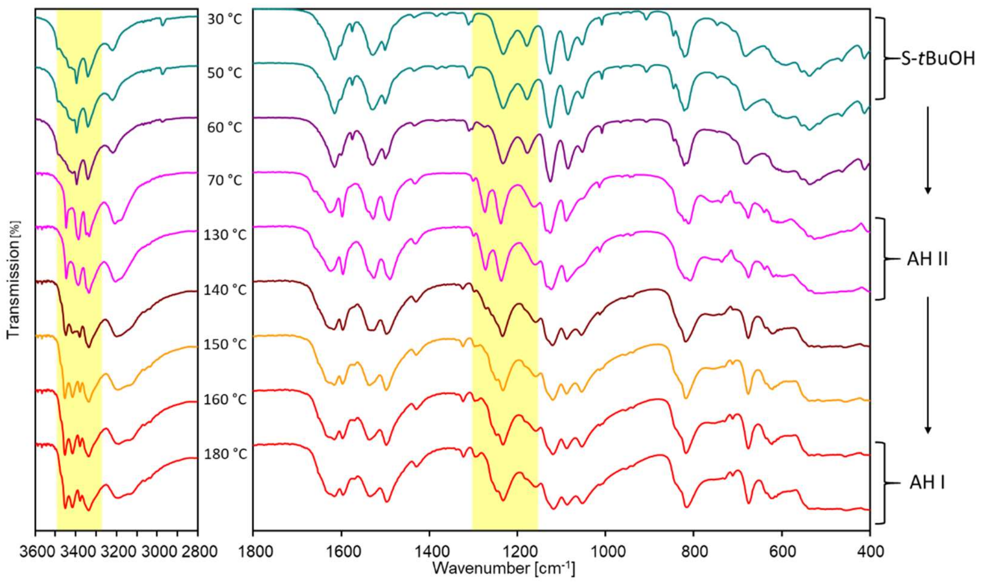

Figure S36. Temperature dependent IR spectroscopy of SGD **S-*t*BuOH**. Distinctive regions are highlighted.

5.2.6. Acetone (ACO) solvate

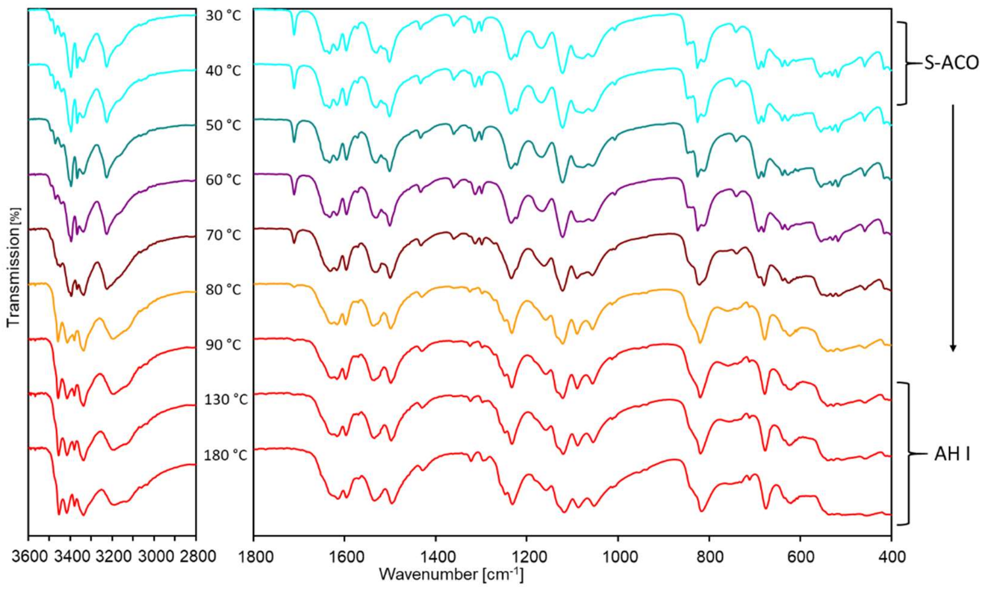

Figure S37. Temperature dependent IR spectroscopy of SGD **S-ACO**.

5.3. Physicochemical data for SGD solid-state forms

Table S20. Physicochemical data for SGD solid-state forms.

Solid-state forms	T_{trs} / °C	$\Delta_{trs}H$ / kJ mol^{-1}	T_{fus} or T_{diss} / °C	$\Delta_{fus}H$ / kJ mol^{-1}	Mass loss / mol S
			Anhydrates		
**AH-I**			188.6 ± 0.3	27.47 ± 0.05	
**AH-II**	>155	0.94 ± 0.07	175 – 176 (HSM)		
**AH-III**			143 – 145 (HSM)		
**AH-V**			187.3 ± 0.4	24.43 ± 0.17	
			Hydrate and Solvates		
**Hy1-I**			143.0 ± 0.1		0.99 ± 0.02
**S-ACO**			123.8 ± 0.9		0.47 ± 0.03
**S-THF**			99.1 ± 0.8		0.95 ± 0.03
**S-DMSO**			95.3 ± 0.9		0.94 ± 0.05
**S-DMA**			118.8 ± 1.1		0.99 ± 0.02
**S-DMF**			109.2 ± 0.8		0.98 ± 0.02
**S-*t*BuOH**			97.2 ± 1.0		0.97 ± 0.02

T_{trs} – transition temperature, $\Delta_{trs}H$ – heat of transition, T_{fus} – melting point, T_{diss} – dissociation temperature (for solvates), $\Delta_{fus}H$ – heat of fusion, *S* – solvate.

References

1. Brandstätter-Kuhnert, M., Polymorphie bei Arzneistoffen. *oesterr. Apoth. Ztg.* **1959,** *13*, 297.
2. Mesley, R. J.; Houghton, E. E., Infrared identification of pharmaceutically important sulfonamides with particular reference to the occurrence of polymorphism. *Journal of Pharmacy and Pharmacology* **1967,** *19* (5), 295-304.
3. Kuhnert-Brandstaetter, M.; Wunsch, S., Polymorphism and solid solution formation in sulfonamides and related compounds. I. *Mikrochimica Acta* **1969,** (6), 1297-1307.
4. Kuhnert-Brandstätter, M.; Bachleitebr-Hofmann, F., I.R.-spektroskopische untersuchungen an enantiotropen kristall-modiflkationen von sulfonamiden. *Spectrochimica Acta Part A: Molecular Spectroscopy* **1971,** *27* (2), 191-198.
5. Yang, S. S.; Guillory, J. K., Polymorphism in sulfonamides. *Journal of Pharmaceutical Sciences* **1972,** *61* (1), 26-40.
6. Alberola, S.; Rambaud, J.; Sabon, F., Study of the polymorphism of sulfaguanidine. *Bulletin de la Societe Chimique de France* **1977,** (3-4), 181-4.
7. Eccles, K. S.; Stokes, S. P.; Daly, C. A.; Barry, N. M.; McSweeney, S. P.; O'Neill, D. J.; Kelly, D. M.; Jennings, W. B.; Ni Dhubhghaill, O. M.; Moynihan, H. A.; Maguire, A. R.; Lawrence, S. E., Evaluation of the Bruker SMART X2S: crystallography for the nonspecialist? *Journal of Applied Crystallography* **2011,** *44* (1), 213-215.
8. Kalman, A.; Czugler, M.; Argay, G., Conformational characteristics of anhydrous sulfaguanidine: computer retrieval and analysis of N-substituted arylsulfonamides. *Acta Crystallographica Section B* **1981,** *37* (4), 868-877.
9. C.H.Koo, H. S. K., W.Shin, C.Choe, *J.Korean Chem.Soc.* **1974,** *18*.
10. Alleaume, M.; Gulko, A.; Herbstein, F. H.; Kapon, M.; Marsh, R. E., Comparison of the dimensions and conformation of the sulfaguanidine moiety in sulfaguanidine monohydrate and trans-dichlorobis(sulfaguanidine)palladium(II). *Acta Crystallographica Section B* **1976,** *32* (3), 669-682.
11. Chisholm, J. A.; Motherwell, S., COMPACK: a program for identifying crystal structure similarity using distances. *Journal of Applied Crystallography* **2005,** *38*, 228-231.
12. Mackenzie, C. F.; Spackman, P. R.; Jayatilaka, D.; Spackman, M. A., CrystalExplorer model energies and energy frameworks: extension to metal coordination compounds, organic salts, solvates and open-shell systems. *IUCrJ* **2017,** *4* (5).
